# CT-Based Peritumoral and Perirenal Fat Radiomics in Renal Cell Carcinoma: A Systematic Review and Meta-Analysis of Grade, Stage, and Adherent Perinephric Fat Prediction

**DOI:** 10.3390/jcm15176720

**Published:** 2026-08-29

**Authors:** Abdulrahman Al Mopti, Ali H. D. Alshehri, Abdulsalam Alqahtani

**Affiliations:** 1 Department of Radiological Sciences, College of Applied Medical Sciences, Najran University, Najran 55461, Saudi Arabia; analmopti@nu.edu.sa (A.A.M.); ahzafer@nu.edu.sa (A.H.D.A.); 2 Health Research Center, Najran University, Najran 55461, Saudi Arabia

**Keywords:** renal cell carcinoma, computed tomography, radiomics, peritumoral fat, perirenal fat, adherent perinephric fat, systematic review, meta-analysis

## Abstract

**Background/Objectives**: Preoperative risk stratification in renal cell carcinoma (RCC) remains challenging for tumor aggressiveness and for adherent perinephric fat (APF) relevant to surgical planning. Although intratumoral radiomics are established, the peritumoral and perirenal fat microenvironment is biologically active and may encode imaging biomarkers. This systematic review and meta-analysis evaluated CT-based peritumoral and perirenal fat radiomics for preoperative grading, staging, and APF prediction and examined whether fat-derived features add incremental value beyond intratumoral models. **Methods**: Following PRISMA 2020 and a registered protocol (PROSPERO CRD420251150155), databases were searched through March 2026, with documented verification searches through August 2026. Eligible studies extracted CT radiomics from peritumoral or perirenal fat in adults with RCC and reported discrimination metrics. Random-effects meta-analyses (restricted maximum likelihood with Hartung–Knapp intervals) were performed; incremental value was estimated from nested within-study AUC differences under predefined rules; and methodological quality was assessed with PROBAST, RQS, METRICS, and TRIPOD, and certainty with an adapted GRADE framework. **Results**: Twenty-five retrospective studies (10,761 patients) were included. Fat radiomics were most consistent for APF prediction (pooled AUC 0.848, 95% CI 0.738–0.917; I^2^ = 0%). Pooled AUCs were 0.772 (0.599–0.884; I^2^ = 93%; 95% prediction interval 0.298–0.964) for grade and 0.813 (0.681–0.899; I^2^ = 72%) for stage. Combined fat-plus-tumor models were numerically higher than tumor-only models in 12 of 15 studies (exploratory *p* = 0.022), but the formally estimable nested increment (stage family, five studies) was small (delta-AUC +0.017; governing modified Hartung–Knapp 95% CI −0.008 to +0.042) and statistically compatible with no true difference; fat-only models showed no evidence of differing from tumor-only models. PROBAST rated 72% of studies at high risk of bias, and 88% originated from China. Certainty ranged from VERY LOW (grade prediction; grade-family fat-versus-tumor comparison) to LOW (all remaining outcomes). **Conclusions**: CT-based peritumoral and perirenal fat radiomics show the most consistent signal for APF prediction, while the measurable increment from adding fat features to tumor models is small and of unestablished clinical utility. Standardized fat segmentation, calibration and decision-curve reporting and prospective multicenter validation are required before clinical translation.

## 1. Introduction

Renal cell carcinoma (RCC) is a heterogeneous malignancy in which preoperative assessment of tumor grade, pathological stage, and operative complexity can influence treatment selection and surgical planning. Globally, RCC accounts for approximately 3% of adult malignancies, with an estimated 434,000 new cases and 155,000 deaths in 2022 [1,2]. Its clinical behavior varies across histological subtypes, and the WHO/ISUP grading system remains central to prognostic evaluation [3,4]. Accurate preoperative characterization is therefore important for distinguishing patients who may be suitable for surveillance or nephron-sparing approaches from those who may require more extensive surgery. Adherent perinephric fat (APF), characterized by inflammatory or fibrotic adherence between the renal capsule and surrounding fat, increases technical difficulty during partial nephrectomy and is associated with more challenging dissection and higher perioperative risk [5]. Although conventional imaging is indispensable for diagnosis and planning, it does not reliably predict tumor aggressiveness or APF before surgery.

Radiomics enables the extraction of large numbers of quantitative imaging features that may capture tissue heterogeneity beyond what is visible to the naked eye [6,7,8]. Standardization efforts such as the Image Biomarker Standardization Initiative (IBSI) have improved the reproducibility of radiomics research across institutions [9]. Most radiomics studies in RCC have focused on intratumoral features [10]. Increasing attention, however, has turned to the fat surrounding the kidney and the tumor. Two anatomically and biologically distinct compartments are relevant here. Perirenal fat, the visceral adipose depot enclosed by Gerota’s fascia, is a metabolically specialized tissue containing brown-like adipocytes with vascular, sympathetic, and endocrine activity, and its adipokine signaling and browning have been described specifically in RCC [11,12]. Peritumoral fat denotes the portion of this depot immediately adjacent to the tumor surface, where the tumor–fat interface concentrates inflammatory, stromal, and metabolic interactions. Tumor-associated inflammation, epithelial–mesenchymal transition, adipocyte remodeling, and desmoplastic change may alter the texture and composition of adjacent fat in ways that are detectable on CT [13]. This has led to growing interest in whether radiomics derived from fat surrounding the tumor carries information beyond that available from the tumor itself.

The potential relevance of adipose tissue radiomics is also supported by studies in other malignancies. Perigastric adipose radiomics has been associated with lymph node metastasis in gastric cancer, mesorectal fat texture has been linked to treatment response in rectal cancer, and peritumoral fat features have improved recurrence prediction in bladder cancer [14,15,16]. These findings suggest that the tumor–fat interface may contain imaging biomarkers of aggressiveness and biological interaction that are not fully captured by intratumoral analysis alone. In RCC, this concept is particularly attractive because the fat surrounding the kidney is not only biologically active but also directly relevant to surgical difficulty, especially in nephron-sparing surgery. Despite this rationale, evidence in RCC remains fragmented, with substantial variation in how fat regions are defined and analyzed.

Two recent systematic reviews have evaluated radiomics for RCC grading, including a meta-analysis of CT-based intratumoral radiomics by Salimi et al. [10] (pooled AUC 0.88) and an MRI-focused review by Broomand Lomer et al. [17]. Neither review specifically addressed radiomics derived from peritumoral or perirenal fat. An important methodological distinction also exists between studies that explicitly isolate adipose tissue using attenuation thresholds or dedicated fat masks and studies that use a peritumoral ring expansion that includes fat together with adjacent non-fat tissues. This distinction, referred to here as Category A (explicit fat segmentation) and Category B (peritumoral ring), has implications for biological interpretation, reproducibility, and potential clinical application. Accordingly, this systematic review and meta-analysis addressed three clinically relevant questions: first, how accurately CT-based radiomics from peritumoral and perirenal fat predict RCC grade, pathological stage, and APF; second, whether fat-derived features provide incremental value beyond intratumoral radiomics; and third, what radiogenomic evidence supports a biological link between fat radiomic features and underlying tumor biology.

## 2. Materials and Methods

### 2.1. Registration and Reporting

The review was registered with PROSPERO (CRD420251150155) and conducted in accordance with PRISMA 2020 guidelines [18]. The PRISMA checklist is provided in Supplementary Table S5, and the TRIPOD-SRMA checklist for systematic reviews of prediction models [19] is provided in Supplementary Table S11.

### 2.2. Search Strategy

Eight electronic databases were searched from inception through March 2026: PubMed, OpenAlex, Semantic Scholar, CrossRef, and Europe PMC, via programmatic interfaces, and Scopus, Web of Science, and IEEE Xplore via institutional exports. Embase could not be searched: a strategy was written, but institutional access was unavailable, and no mediated route could be arranged. This is acknowledged as a limitation, and its likely impact was assessed through the verification exercises described below. Search terms combined concepts of radiomics/texture analysis, peritumoral/perirenal/perinephric fat, renal cell carcinoma, and CT imaging (full strategies and per-database yields in Supplementary Table S1). Backward and forward citation chasing of included studies and relevant reviews was performed. Records were restricted to publication years 2015–2026, reflecting the emergence of feature-based radiomics as a defined methodology in the early 2010s [6]; the earliest study meeting our eligibility criteria was published in 2018. Two verification exercises accompany this restriction: re-running the search strategies from inception through 2014 on all API-accessible databases identified no eligible earlier study, and an indexing check confirmed that all 38 studies assessed at full text are indexed in the accessible sources (Supplementary Table S12); such checks partially probe, but cannot fully quantify, the risk from records indexed only in inaccessible databases. A search update through August 2026 was also run; eligible studies published after the search date were not incorporated into the synthesis and are identified in the Discussion. No language restrictions were applied.

### 2.3. Eligibility Criteria and Fat ROI Classification

Studies were eligible if they: (a) included adults with renal masses or histologically confirmed RCC; (b) extracted CT-based radiomics explicitly from peritumoral fat (PTF) or perirenal fat (PRF) regions; (c) reported discrimination metrics (AUC and sensitivity/specificity) for grade, stage, or APF classification, for radiogenomic associations, or for secondary RCC endpoints (metastasis, recurrence, capsule invasion, Ki-67 expression, and histologic subtype) synthesized descriptively; and (d) were original research with sufficient data for analysis. Reviews, conference abstracts, case reports, non-CT imaging studies, and studies using perilesional rings without fat tissue involvement were excluded.

A classification of fat ROI methodology was applied with the following operational rules:

Category A (explicit fat segmentation): the pipeline contains a documented adipose-isolation step: attenuation thresholding (study-specific windows, tabulated per study in Supplementary Table S9; a uniform window was not required), a dedicated fat-segmentation algorithm, or manual fat-only delineation.

Category B (peritumoral ring): a fixed-distance dilation without an adipose-isolation step, capturing a ring that includes fat together with renal parenchyma and other tissues. Such rings were retained when they plausibly sampled perinephric or peritumoral fat; rings with no fat involvement were excluded (code E7; three studies, Supplementary Table S2).

Two further rules govern model eligibility for the fat-specific analyses. An ROI that contains the entire tumor volume is treated as a combined tumor-plus-fat construct and never as a fat or peritumoral estimate. A model whose feature set is documented to combine intratumoral with peritumoral features is likewise treated as combined, whatever its label. This classification was developed post hoc during data extraction in response to heterogeneity identified at that stage. All 25 assignments were verified against these operational rules in two independent blinded passes with consensus adjudication and verbatim source quotations; per-study definitions and classification rationales are given in Supplementary Table S9.

### 2.4. Study Selection and Data Extraction

Two reviewers independently performed title-and-abstract screening and full-text assessment; a third reviewer resolved disagreements. One reviewer extracted the data, and a second verified all extracted values; discrepancies were adjudicated by consensus. When a study reported multiple estimates, selection followed a prespecified hierarchy: external validation over internal hold-out, internal over cross-validation, cross-validation over apparent performance; within a tier, the authors’ designated primary model, CT phase, and shell; and ties resolved by first-reported estimate, never by the largest AUC. Within-study comparisons were admissible only when both models shared the endpoint, evaluation cohort, validation tier, and CT phase; for combined models, the classifier had to match as well. Every value entering an analysis was verified against the primary full text in two independent passes, with published Supplementary Materials consulted where the deciding detail resided there. The complete extracted dataset is provided in Supplementary Table S6.

### 2.5. Quality Assessment

Quality assessment was performed independently by two reviewers, with disagreements resolved by a third. Four complementary tools were applied:

PROBAST (Prediction model Risk Of Bias ASsessment Tool) [20] for risk of bias across four domains, as specified in the protocol for prediction model studies: participants, predictors, outcome, and analysis.

Radiomics Quality Score (RQS) [8] for radiomics-specific methodology (16 items, maximum 36 points).

METRICS (METhodological RadiomICs Score) [21], developed as a complement to the CLEAR reporting guideline [22] for methodological quality, using 30 weighted items across 9 domains.

TRIPOD (Transparent Reporting of a multivariable prediction model for Individual Prognosis or Diagnosis) [23] for reporting adherence (12 key applicable items). A self-appraisal against AMSTAR 2 is provided in Supplementary Table S8.

### 2.6. Statistical Analysis

For the discrimination pools, AUC values were logit-transformed and standard errors derived from reported 95% CIs using the logit-scale interval width so that asymmetric and boundary-truncated intervals are handled without symmetric-Wald assumptions; no standard error was approximated from sample size alone. Random-effects models were fitted with the restricted maximum likelihood (REML) heterogeneity estimator, recommended for meta-analysis of prediction model performance [24], with Hartung–Knapp confidence intervals [25]; for pools of five or fewer studies, the modified Hartung–Knapp interval (the Hartung–Knapp standard error truncated below at the corresponding standard-normal model’s standard error, with a t quantile) was computed alongside and governs interpretation, guarding against spuriously narrow intervals when estimated heterogeneity is low. DerSimonian–Laird [26] and Paule–Mandel estimators were examined in sensitivity analyses. Heterogeneity was quantified using I^2^, τ^2^, Cochran’s Q, and 95% prediction intervals [27], and prediction intervals were treated as the primary statement of expected performance in a new setting. Subgroup and sensitivity analyses included Category A versus B, external-validation-only, PROBAST low-risk-only, sample size ≥ 200, alternate external cohorts where studies were validated in more than one, leave-one-out influence, and omission of studies sharing a public source cohort (TCGA-KIRC) within the same pool. Formal small-study-effect tests were not used inferentially because every pool contained fewer than ten studies; reporting bias was assessed qualitatively, with exploratory Egger regression [28,29] and funnel plots confined to the supplement.

Incremental value was estimated from within-study AUC differences on the raw AUC scale, restricted to nested pairs: models evaluated on the same patients at the same validation tier, where the combined model unites the tumor and fat feature sets without adding clinical or other predictors. Pairs failing these rules (single merged tumor-plus-ring masks without a separable fat feature set, or combined models incorporating clinical variables, kidney features, or reader input) were excluded from weighted pooling and are displayed descriptively. Variances used, in order of preference, were exact paired statistics reported by the primary study (DeLong differences or exact two-sided *p*-values) or per-model confidence intervals combined as Var(d) = SE1^2^ + SE2^2^ − 2r × SE1 × SE2 with the within-study correlation r assumed over a predefined grid (0, 0.25, 0.50, 0.75, 0.90); the modified Hartung–Knapp interval governs these small pools, a logit-scale contrast was run as a sensitivity analysis, and a conclusion is claimed only if direction and inference are invariant across the whole grid. Pools were computed per endpoint family (grade; stage), with a stopping rule of no pooled estimate from fewer than 3 complete-case pairs; a cross-family pool is exploratory only. Wilcoxon signed-rank comparisons of the same within-study differences are reported as exploratory, unweighted descriptive tests and are labeled as such throughout. Evidence certainty was graded using an adapted GRADE framework for prognosis and prediction research [30,31,32], with the target certainty stated for each outcome. For the incremental analyses, imprecision was assessed by whether the governing interval excluded no difference and indirectness and applicability by the absence of calibration and decision-curve evidence from which a clinically important difference in discrimination could be defined; published guidance cautions against interpreting AUC differences as measures of clinical value [33]. All analyses were performed using R version 4.3.3 (R Foundation for Statistical Computing) with the metafor package version 4.8.0.

### 2.7. Deviations from Protocol

Several prespecified analyses were not feasible: the bivariate random-effects model for sensitivity/specificity (no 2 × 2 tables available); meta-regression (fewer than 10 studies per covariate); and the CLAIM checklist (no end-to-end deep-learning primary models). Calibration, NRI/IDI, and decision-curve outcomes could not be meta-analyzed because no primary study reported them in poolable form. Four elements of the analysis were not specified in the registered protocol and were fixed before the corresponding analyses were run: the fat ROI classification and model-composition eligibility rules (Section 2.3), developed during extraction to handle the heterogeneity of fat definitions found there; the REML primary estimator with modified Hartung–Knapp intervals; the nested delta-AUC framework for incremental value; and the graded reporting of calibration. Their specifications are documented in Supplementary Table S10.

## 3. Results

### 3.1. Study Selection

**Figure 1:**
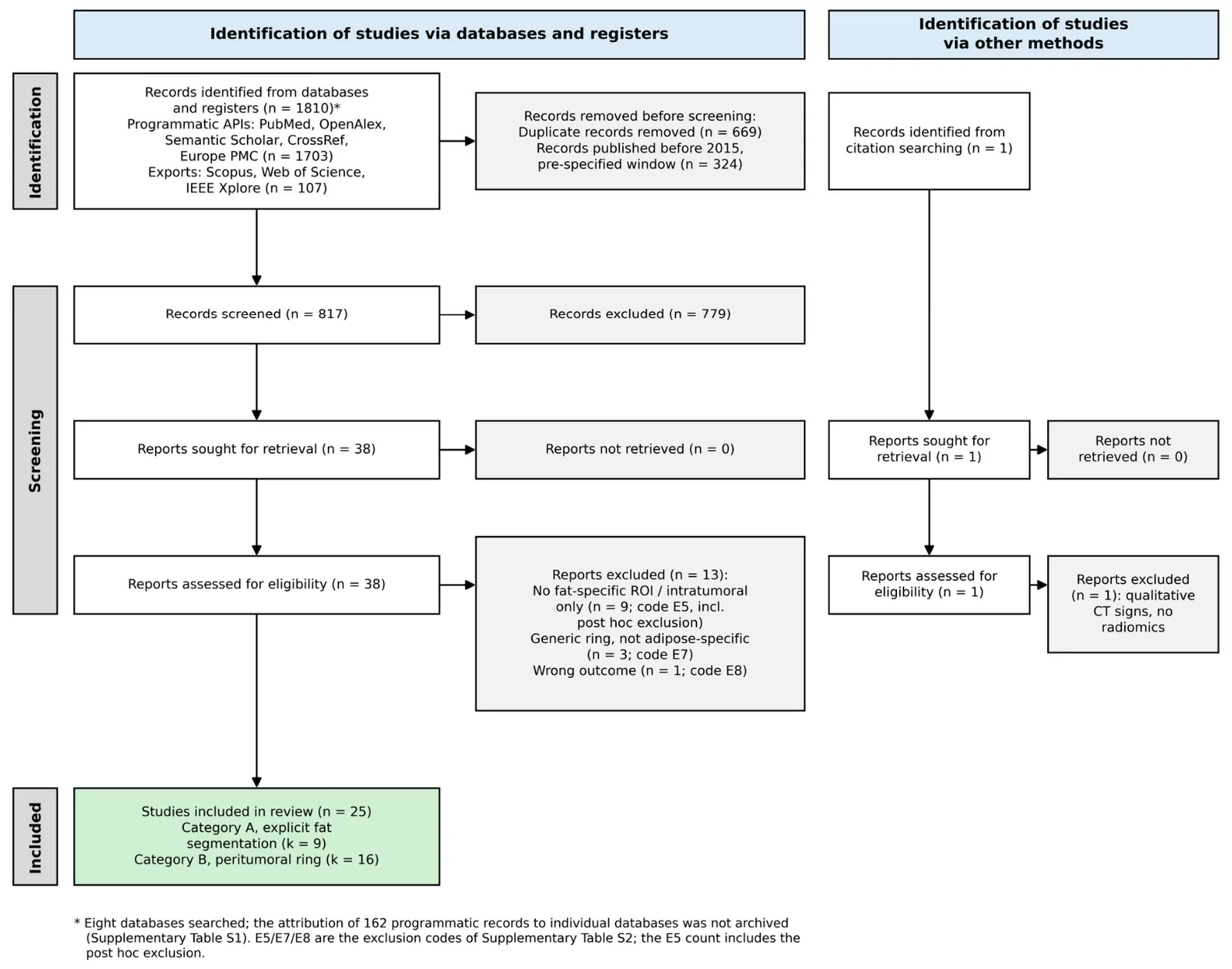
Study selection according to the PRISMA 2020 statement. The databases-and-registers arm comprises 1810 records; the asterisk indicates that eight databases were searched and that 162 programmatic records could not be attributed to an individual database (per-database yields in Supplementary Table S1). The other-methods arm comprises one citation-chasing record, assessed and excluded. E5, E7, and E8 are the exclusion codes of Supplementary Table S2. Included studies were classified by fat region-of-interest (ROI) methodology as Category A (explicit adipose segmentation; k = 9) or Category B (peritumoral ring dilation; k = 16). Abbreviations: n, number of records; PRISMA, Preferred Reporting Items for Systematic Reviews and Meta-Analyses; ROI, region of interest.

Database searches identified 1810 records: 1703 through programmatic interfaces (PubMed, OpenAlex, Semantic Scholar, CrossRef, and Europe PMC) and 107 through Scopus, Web of Science, and IEEE Xplore exports; one additional record was identified through citation chasing. After removal of 669 duplicates and 324 records published before 2015, 817 records were screened and 779 excluded, leaving 38 reports for full-text assessment. Thirteen studies were excluded at the full-text stage (reasons in Supplementary Table S2). The citation-chasing record was assessed and excluded (qualitative CT signs without radiomic feature extraction). Twenty-five studies were included in the final synthesis (Figure 1).

### 3.2. Study Characteristics

The 25 included studies (2018–2026) encompassed 10,761 patients (range 70–1661 per study) [34,35,36,37,38,39,40,41,42,43,44,45,46,47,48,49,50,51,52,53,54,55,56,57,58]. Twenty-two studies (88%) originated from China, with one each from the USA, France, and Greece. All were retrospective. Sixteen studies (64%) enrolled only clear cell RCC; the remainder analyzed mixed or unselected histologies (Table 1). Against the operational rules of Section 2.3, nine studies (36%) were classified as Category A (explicit fat segmentation) and 16 (64%) as Category B (peritumoral ring). Category assignment followed the documented segmentation pipeline rather than the label used by the primary study: for example, Ding et al. [35] define a geometric 5 mm band around the renal contour with manual removal of gross non-adipose structures, which meets the Category B definition despite describing a perirenal fat region.

Predicted outcomes (some studies addressed multiple endpoints) included WHO/ISUP or Fuhrman grade (nine studies addressed; six contributed a fat-only estimate to the pool), pathological T-stage, perirenal fat invasion, pT3a upstaging, or surrounding-tissue invasion (seven addressed; six poolable), APF (four), and additional endpoints (metastasis, recurrence, capsule invasion, Ki-67 expression, radiogenomic associations, and histologic subtype). For the fat-specific model tier, eleven studies reported external validation, six internal hold-out, two cross-validation, two only apparent performance, and four study-level validation without a separately validated fat-model tier. PyRadiomics was the most common feature-extraction tool (19/25). Ring thicknesses ranged from 1 to 10 mm in Category B studies (Table 1; full ROI definitions per study in Supplementary Table S9, and a graphical summary of study characteristics in Supplementary Figure S16). 

### 3.3. Grade Prediction

**Figure 2:**
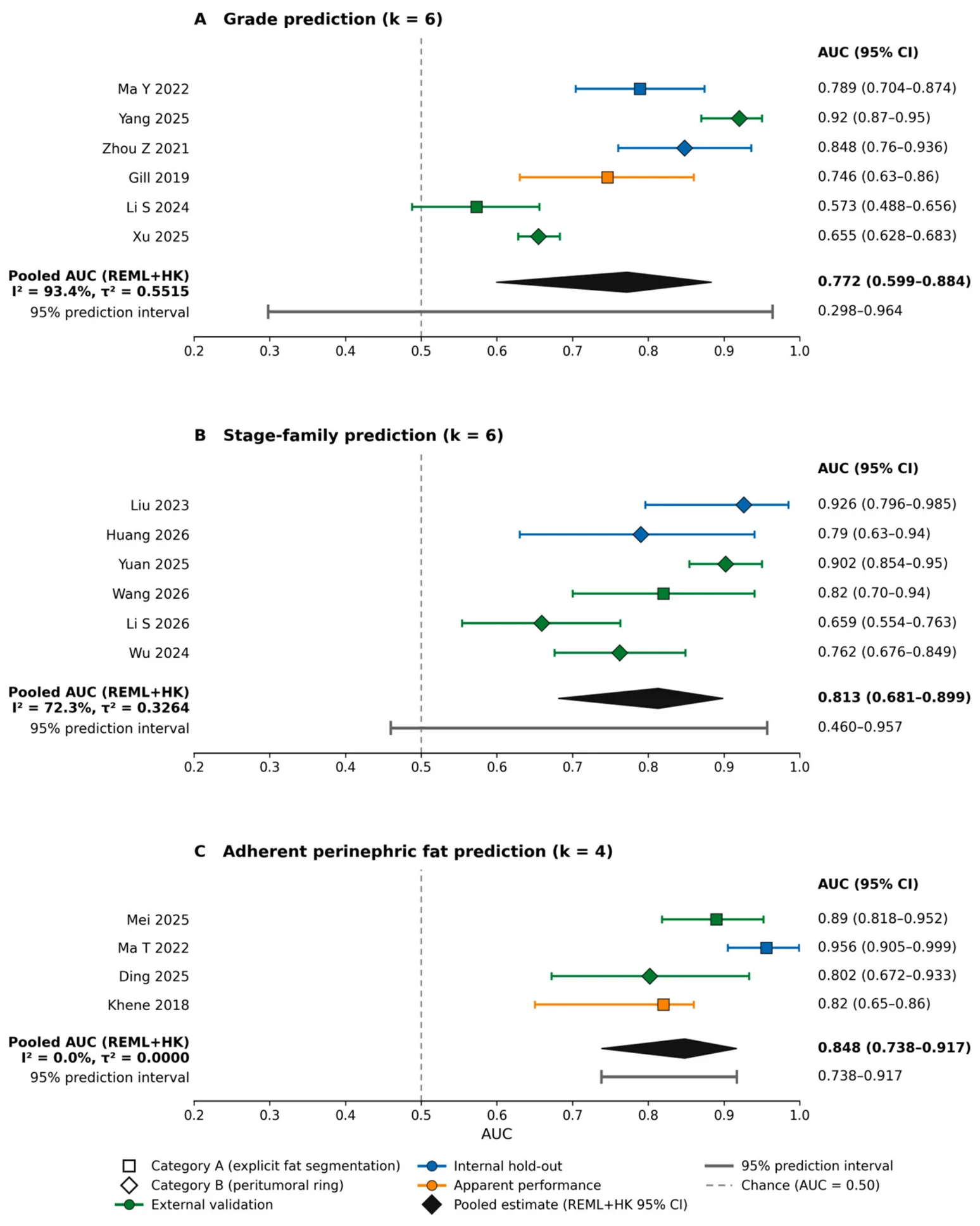
Forest plots of random-effects meta-analyses (REML with Hartung–Knapp intervals) for CT-based peritumoral and perirenal fat radiomics. Panel (**A**) grade prediction (k = 6) [36,40,47,53,54,58]; panel (**B**) stage-family prediction (k = 6) [37,41,44,50,51,56]; panel (**C**) adherent perinephric fat prediction (k = 4) [35,38,46,48]. Marker shape encodes fat ROI methodology (square, Category A; diamond, Category B); color encodes the fat-model validation tier. Pooled estimates were computed on the logit-AUC scale and back-transformed. The dotted line at 0.50 denotes chance performance. Prediction intervals are displayed beneath each pooled diamond and are the primary statement of expected performance in a new setting. Abbreviations: AUC, area under the receiver operating characteristic curve; CI, confidence interval; k, number of studies; PI, prediction interval; REML, restricted maximum likelihood; ROI, region of interest.

Six studies met the model-composition criteria of Section 2.3 for the fat-only grade pool (Figure 2A; enlarged in Supplementary Figure S1). Studies whose fat-related models contain the entire tumor volume (Luo et al. [45]) or combine intratumoral with peritumoral features by design (Chen et al. [34]) do not yield a fat-only estimate and are synthesized as combined constructs (Table 1, footnote e). The pooled AUC was 0.772 (95% CI 0.599–0.884), with substantial heterogeneity (I^2^ = 93%, τ^2^ = 0.55). The 95% prediction interval spanned 0.298 to 0.964: in a new setting, expected fat-only performance for grade is compatible with anything from clearly worse than chance to excellent, and the pooled point estimate should not be read as a generalizable performance figure.

Sensitivity analyses were consistent in direction but uniformly imprecise (governing modified Hartung–Knapp intervals at k ≤ 5): external validation only (k = 3) 0.752 (0.150–0.981; I^2^ = 98%); PROBAST low-risk only (k = 3) 0.809 (0.303–0.976); n ≥ 200 (k = 5) 0.778 (0.543–0.911); omission of either study drawing on the shared TCGA-KIRC source (Zhou Z 2021, Yang 2025) shifted the pooled estimate to 0.757 and 0.718, respectively. Leave-one-out analysis identified Yang 2025 as the most influential: omitting it yielded a pooled AUC of 0.718 (I^2^ = 86%). Influence and subset estimates for all three pools are plotted in Supplementary Figures S6 and S7. The Category A versus B comparison is exploratory at three studies per arm (Supplementary Figure S4): Category A (explicit fat) 0.703 (0.365–0.907; I^2^ = 80%) and Category B (ring) 0.828 (0.314–0.981; I^2^ = 94%), between-subgroup *p* = 0.34; with intervals this wide, no conclusion about equivalence or difference is warranted. Formal small-study-effect testing was not undertaken (k < 10); the exploratory funnel plot and Egger regression appear in Supplementary Figure S5. GRADE certainty was VERY LOW, downgrading for risk of bias (3/6 HIGH, including one apparent-performance study), inconsistency (I^2^ = 93%; prediction interval spanning chance), and imprecision (Table 2).

### 3.4. Stage Prediction

Six studies contributed to the stage-family pool (pathological T-stage, pT3a upstaging, perirenal fat invasion, or surrounding-tissue invasion; Figure 2B, enlarged in Supplementary Figure S2), including Wu et al. [51], for which the externally validated peritumoral estimate and its interval are reported in the study’s electronic Supplementary Material (Table 1, footnote i). The pooled AUC was 0.813 (95% CI 0.681–0.899; I^2^ = 72%), with a 95% prediction interval of 0.460–0.957. The outcome family mixes related but non-identical constructs, which is disclosed as a source of conceptual heterogeneity.

The two largest studies (Yuan 2025, n = 1516; Li S 2026, n = 1661) reported divergent results (0.902 versus 0.659), contributing substantially to heterogeneity. Leave-one-out analysis identified Yuan 2025 as the most influential: omitting it yielded a pooled AUC of 0.771 (I^2^ = 51%). Sensitivity analyses (governing modified Hartung–Knapp intervals k ≤ 5) were as follows: external-only (k = 4) 0.797 (0.565–0.922); PROBAST low-risk only (k = 3) 0.810 (0.346–0.972); n ≥ 200 (k = 5) 0.795 (0.633–0.897); substituting Yuan’s second external cohort (TCIA) 0.777 (0.676–0.854; I^2^ = 49%); and omitting the studies sharing TCGA-derived patients changed the estimate by 0.006 or less. A Category-A-only stage analysis is not estimable (one study): Wang 2026 reported 0.82 (0.70–0.94). The Category-B-only pool (k = 5) was 0.814 (0.629–0.919). Exploratory Egger regression and the funnel plot are reported in Supplementary Figure S5, with reported AUC against cohort size across all studies in Supplementary Figure S10. GRADE certainty was LOW, downgrading for risk of bias (3/6 HIGH) and inconsistency (Table 2).

### 3.5. Adherent Perinephric Fat Prediction

Four studies formed the APF pool (Figure 2C; enlarged in Supplementary Figure S3). Three used explicit adipose segmentation (Category A) and one a geometric perirenal band (Category B); all four sample the perirenal fat compartment. The pooled AUC was 0.848 (95% CI 0.738–0.917) with no detected heterogeneity (I^2^ = 0%, τ^2^ = 0). The reference standard differed: surgically confirmed adhesion in Khene 2018, Ma T 2022, and Ding 2025, versus a radiologist-assigned MAP score ≥ 3 across all Mei 2025 cohorts, an imaging surrogate that also shares its source images with the model features. Restricting to the three surgical-reference studies left the estimate materially unchanged (0.826, governing modified Hartung–Knapp interval 0.615–0.934; I^2^ = 0%); substituting Mei’s second external cohort (KiTS23) lowered it to 0.781 (0.556–0.910; I^2^ = 58%). Leave-one-out confirmed no single-study dependence beyond a 0.022 shift (omitting Mei: 0.826). A post hoc sensitivity analysis restricted to the three Category A studies using explicit adipose segmentation yielded a pooled AUC of 0.863 (governing modified Hartung–Knapp 95% CI 0.643–0.956; I^2^ = 22.8%; τ^2^ = 0.0623; k = 3; Supplementary Figure S17). Excluding the single Category B study therefore did not materially alter the pooled estimate, although the wider interval and the availability of only one Category B study preclude any inference about superiority or equivalence between ROI constructions.

This consistency may reflect the methodological alignment between the segmented tissue (perirenal fat) and the predicted outcome (adherence of that same tissue to the renal capsule), thereby reducing the noise introduced when fat features must predict outcomes that arise primarily from tumor biology, such as grade or stage.

The low statistical heterogeneity indicates consistent discrimination estimates, but all four studies carried HIGH PROBAST risk of bias and two lacked any independent validation. GRADE certainty was LOW, downgrading two levels for risk of bias; consistency and precision were insufficient to offset design-level limitations, and the reference-standard difference is disclosed as indirectness (Table 2).

### 3.6. Incremental Value

Incremental value was assessed from within-study AUC differences on the raw scale, restricted to nested pairs at matched validation tiers and pooled per endpoint family (Section 2.6). Six studies reported exact paired statistics for the relevant contrast (Liu [44], Zhou Z [58], Yuan [56], Li S 2026 [41]; Xu [53], Zhou J [57]), which were used in preference to interval-derived variances. Pairs drawn from different validation cohorts, different validation tiers, or different classifiers were not admissible, and the full pair inventory with each admissibility verdict is given in Supplementary Table S10.

**Figure 3:**
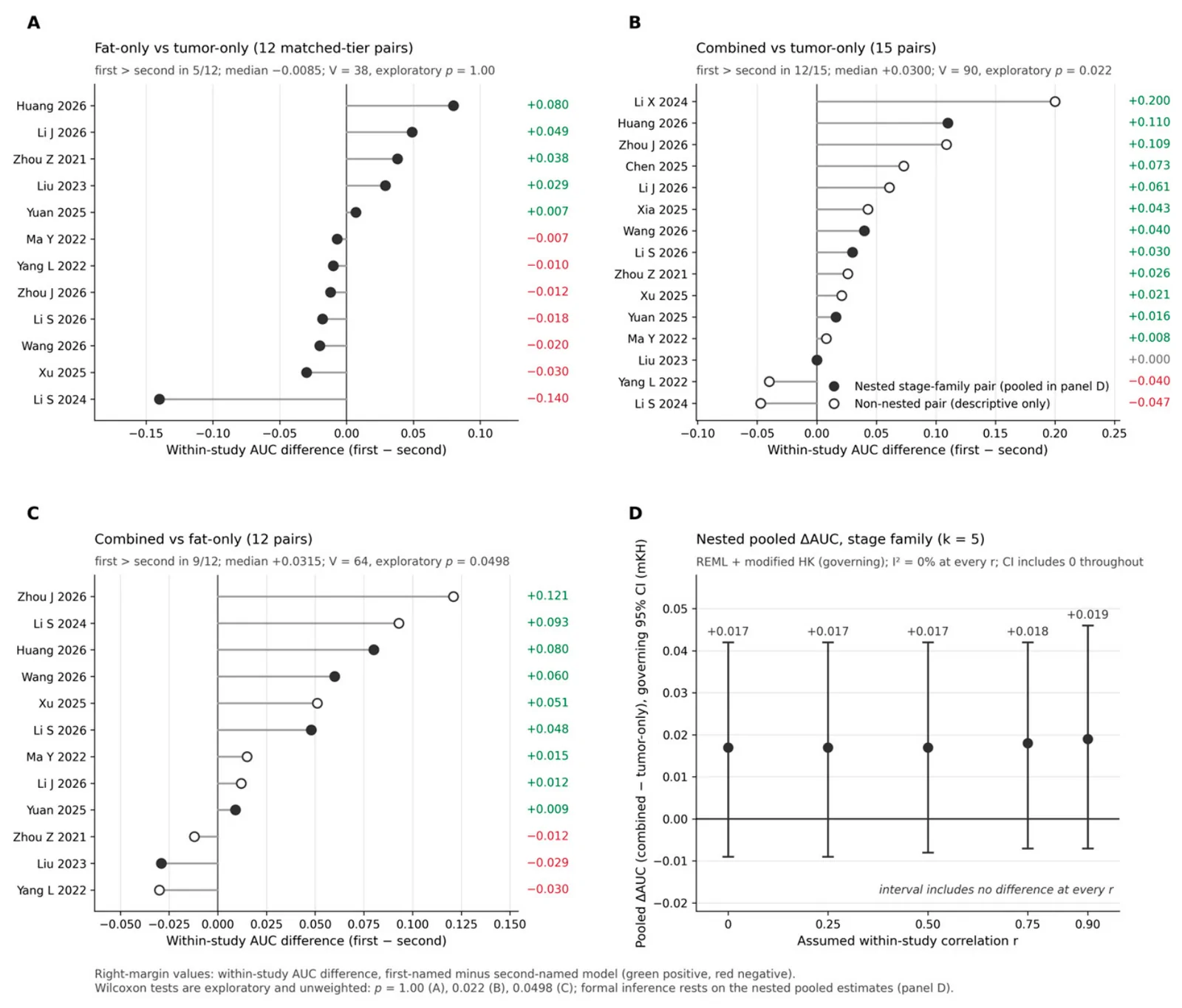
Within-study model comparisons. Panel (**A**) shows fat-only versus tumor-only (12 matched-tier pairs) [37,39,40,41,44,47,50,53,55,56,57,58]; panel (**B**) shows combined versus tumor-only (15 pairs, nested and non-nested distinguished) [34,37,39,40,41,42,44,47,50,52,53,55,56,57,58]; panel (**C**) shows combined versus fat-only (12 pairs) [37,39,40,41,44,47,50,53,55,56,57,58]. Right margin: within-study difference, first-named minus second-named model. Panel (**D**) shows pooled nested delta-AUC for the stage family across the correlation grid r = 0–0.90. Wilcoxon tests are exploratory and unweighted; formal inference rests on the nested pooled estimates. Abbreviations: AUC, area under the curve; r, assumed within-study correlation between paired AUC estimates.

Descriptively, combined fat-plus-tumor models showed numerically higher discrimination than tumor-only models in 12 of 15 studies reporting both at a matched tier (median difference +0.030; exploratory Wilcoxon *p* = 0.022) and than fat-only models in nine of 12 (median +0.032; exploratory *p* = 0.0498); fat-only and tumor-only models did not differ (five of 12; median −0.009; exploratory *p* = 1.00) (Figure 3).

The weighted analysis tempers the descriptive picture. In the stage family (five nested pairs), the pooled increment of combined over tumor-only models was +0.017 at r = 0.5, with I^2^ = 0% and stable leave-one-out behavior; the governing modified Hartung–Knapp interval was −0.008 to +0.042 (Hartung–Knapp +0.003 to +0.031) and included zero at every correlation value. The combined-versus-fat increment was +0.014 (governing CI −0.026 to +0.054), also including zero throughout. Fat-only versus tumor-only differences pooled to +0.007 (−0.056 to +0.070; I^2^ = 0%) in the stage family (five pairs) and −0.030 (−0.152 to +0.091; I^2^ = 80%) in the grade family (four pairs) and were null in both. Logit-scale sensitivity contrasts agreed with the raw-scale results across all families and all intervals that include zero. In the grade family, only two nested combined-model pairs were available (Ma Y [47] +0.008; Zhou Z +0.026, exact p = 0.200), below the pooling threshold of three. Certainty is LOW for the incremental outcomes, except the grade-family fat-versus-tumor comparison, which carries three serious downgrade domains and is rated VERY LOW. Taken together, the combined models showed numerically higher discrimination in most reporting studies, while the estimable pooled increment is small and statistically compatible with no true difference, and fat-only models show no evidence of replacing tumor models.

### 3.7. Quality Assessment Results

**Figure 4:**
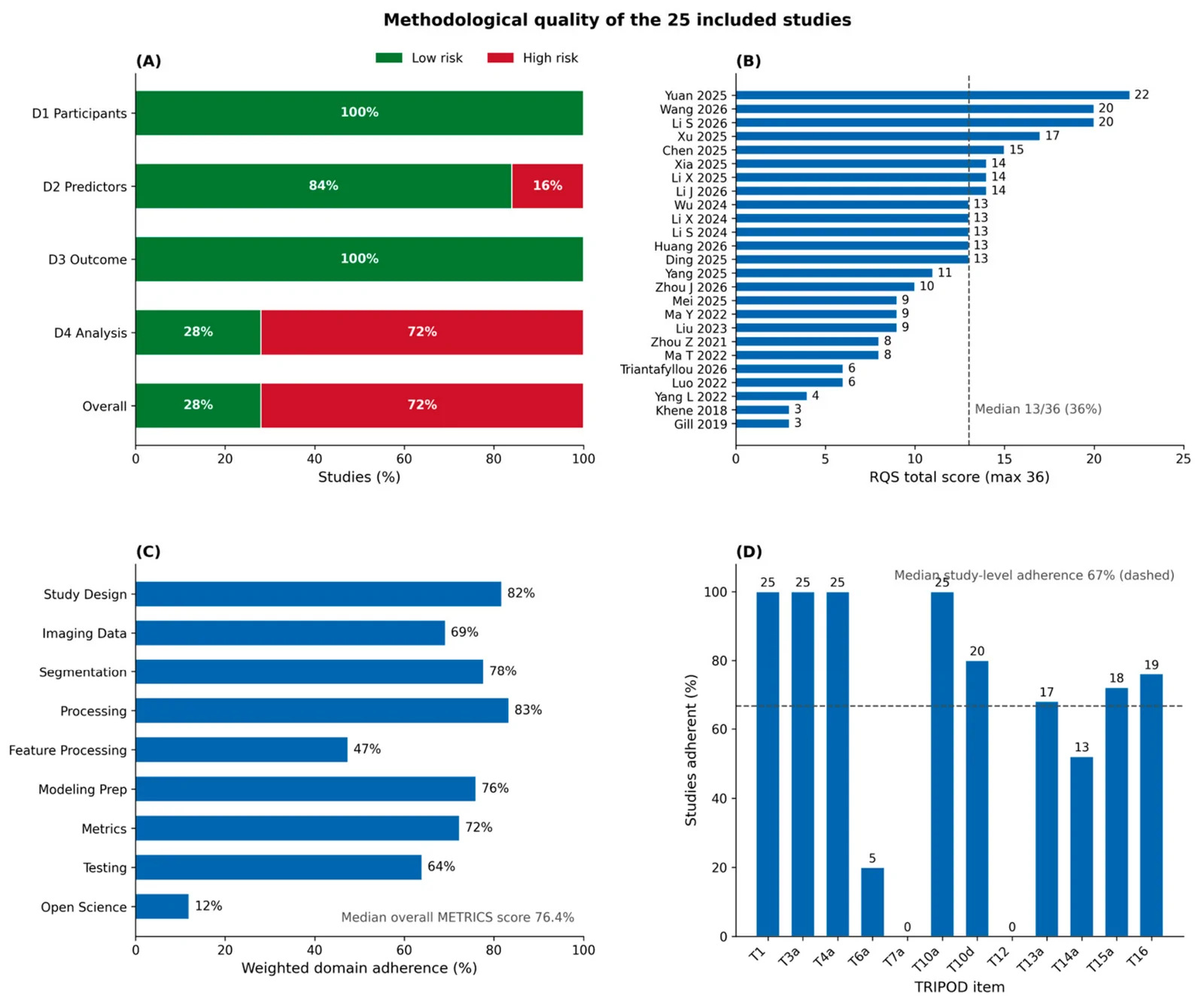
Methodological quality of the 25 included studies: (**A**) PROBAST domain and overall judgments; (**B**) per-study RQS totals [34,35,36,37,38,39,40,41,42,43,44,45,46,47,48,49,50,51,52,53,54,55,56,57,58] (median 13/36, 36%); (**C**) METRICS scores (median 76.4%); (**D**) TRIPOD item adherence (median 67%). Abbreviations as previously defined.

PROBAST PROBAST identified 18 of 25 studies (72%) as overall HIGH risk of bias (Figure 4A). Domain 4 (Analysis) drove this result (18/25 HIGH), predominantly due to insufficient events-per-variable ratios and the absence of external validation of the fat-specific model. Domains 1, 2, and 3 showed HIGH-risk rates of 0%, 16%, and 0%. Per-study domain judgments are plotted in Supplementary Figure S11.

The median RQS was 13/36 (36%; range 3–22), indicating low radiomics-specific methodological quality (Figure 4B; per-study totals in Supplementary Figure S12). No study included phantom calibration or prospective design, and only two performed imaging at multiple time points. Calibration reporting was graded at item level in three tiers: ten studies (40%) displayed calibration curves; three (12%) reported numeric calibration statistics (Brier scores in one; Hosmer–Lemeshow statistics in two, one of which does not state the metric’s scale unambiguously); one further study reported Hosmer–Lemeshow results only as a threshold statement, with values in a supplement that could not be retrieved; and none reported calibration in a poolable form (slope, intercept, or observed-to-expected ratio).

**Figure 5:**
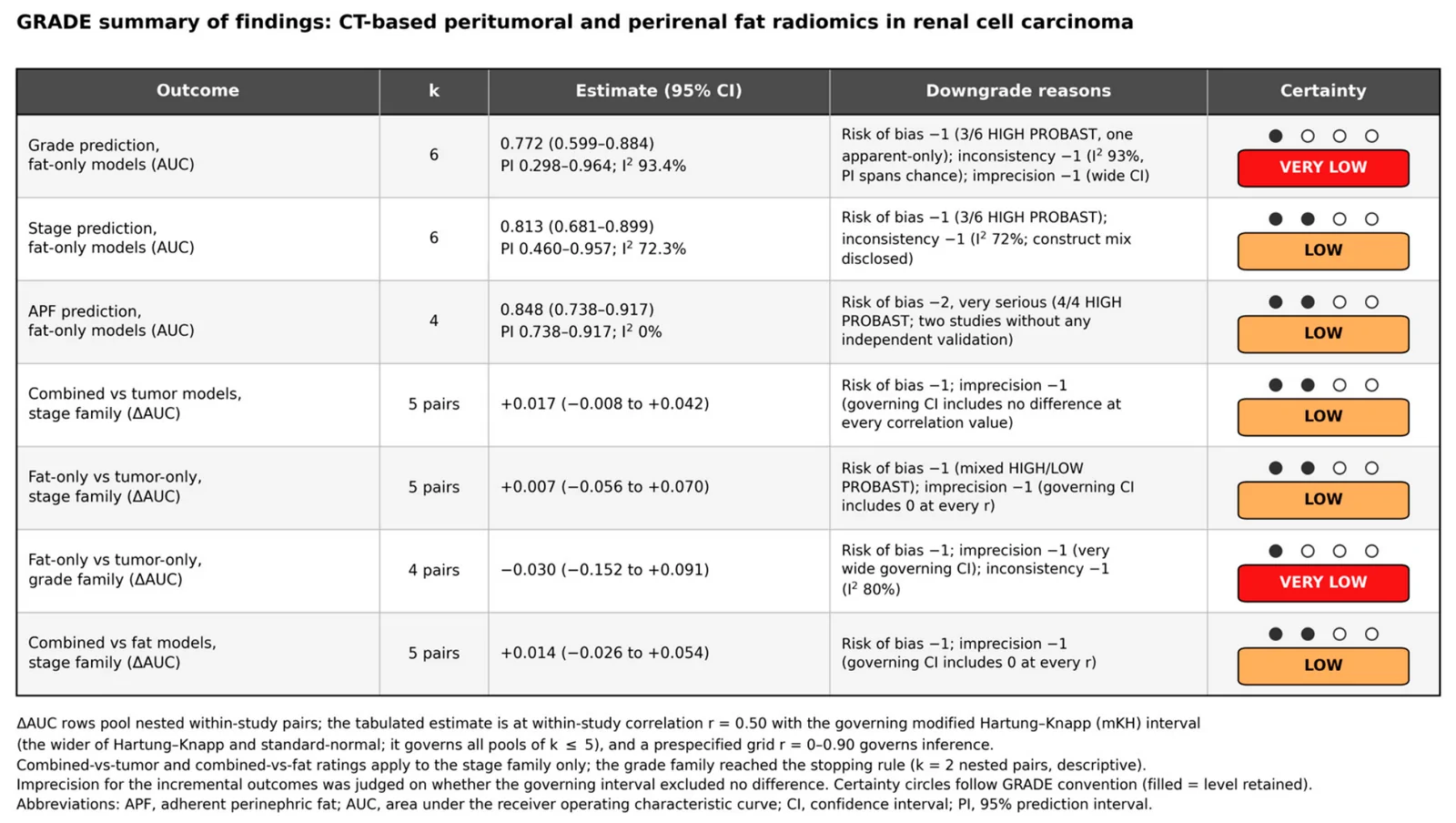
GRADE summary of findings. Certainty for each review outcome under the adapted GRADE framework for prediction research, with the target of certainty stated per outcome and domain-level judgments as reported in Section 3.3, Section 3.4, Section 3.5 and Section 3.6: grade prediction VERY LOW; stage prediction LOW; APF prediction LOW; combined-versus-tumor, fat-versus-tumor (stage family), and combined-versus-fat LOW; fat-versus-tumor (grade family) VERY LOW. Abbreviations: APF, adherent perinephric fat; GRADE, Grading of Recommendations Assessment, Development and Evaluation.

The median METRICS score was 76.4% (range 38.9–90.4%), with six studies rated excellent, 15 good, three moderate, and one low (Figure 4C; per-study scores in Supplementary Figure S13). Weighted domain-level adherence identified Open Science (12%), Feature Processing (47%), and Testing (64%) as the weakest domains and Processing (83%), Study Design (82%), and Segmentation (78%) as the strongest. The Spearman correlation between overall RQS and METRICS scores was 0.895 (*p* < 0.001; Supplementary Figure S15). Median TRIPOD reporting adherence was 67% (Figure 4D; item-level adherence in Supplementary Figure S14): four of the 12 key applicable items were reported by all 25 studies, while three items were reported by five or fewer, and two of them by none. Complete study-level results are in Table 3, with item-level scoring in Supplementary Table S7. The certainty of evidence for each review outcome is summarized in Figure 5.

### 3.8. Feature Convergence and Radiogenomics

Across 18 studies reporting named features, Busyness (NGTDM) appeared in 7 (39%), followed by SizeZoneNonUniformity (5), ZoneEntropy (5), Kurtosis (5), and Correlation (5); the full study-by-feature matrix is in Supplementary Figure S8. Texture-class features accounted for 69% of selected features, first-order 19%, and shape 12% (Supplementary Figure S9). Six studies reported SHAP-based interpretability analyses, with Busyness and ZoneEntropy among the highest-ranked features in each; feature-importance rank within a fitted model does not identify a biological determinant.

Four studies performed exploratory radiogenomic analyses using TCGA-KIRC data (Supplementary Table S3); all are retrospective correlation analyses and are presented as hypothesis-generating. Pathways recurring in at least two studies were epithelial–mesenchymal transition and immune or inflammatory signaling (IL6-JAK-STAT3 and lymphocyte-mediated immunity); extracellular matrix remodeling (collagen binding and Hippo signaling) was reported in one study. Wang 2026 reported a correlation between perirenal adipose browning markers and angiogenesis-related gene sets, and Li S 2026 a correlation between geometric features and epithelial–mesenchymal transition gene sets. These correlations offer preliminary biological plausibility for fat-derived signals; no causal inference is possible.

## 4. Discussion

This synthesis covers 25 studies and 10,761 patients and evaluates CT-based radiomics from peritumoral and perirenal fat in RCC across three endpoints; we identified no previous review restricted to these compartments. Its main findings are three. First, the most consistent evidence concerns APF prediction: a pooled AUC of 0.848 (0.738–0.917) with no detected heterogeneity, robust to restriction to surgically confirmed reference standards (0.826; I^2^ = 0%). Second, there is no evidence that fat-only models outperform intratumoral models for tumor-intrinsic endpoints, although the intervals are too wide to exclude a difference in either direction: the pooled fat-versus-tumor difference is compatible with zero in both endpoint families, and the grade pool’s prediction interval (0.298–0.964) spans chance to excellence. Third, combined fat-plus-tumor models showed numerically higher discrimination than tumor-only models in 12 of 15 exploratory comparisons, yet the estimable nested increment in the stage family was +0.017 with a governing interval of −0.008 to +0.042: small and statistically compatible with no true difference. Certainty ranges from VERY LOW to LOW across outcomes, precluding clinical adoption at this stage. The pooled fat-only estimate for grade (0.772) is lower and less certain than the 0.88 reported for intratumoral grading radiomics in a separate review [10]; the two syntheses differ in study sets, endpoints, and methods, so this is a comparison between reviews rather than a head-to-head result. Any value fat radiomics carries appears to lie alongside rather than instead of tumor analysis, and the measurable size of that contribution is currently small and uncertain.

Peritumoral and perirenal fat are not interchangeable labels, and the distinction carries both biological and clinical weight. Perirenal fat is the visceral depot enclosed by Gerota’s fascia: a specialized tissue with brown-like adipocytes, dense vascular and sympathetic supply, and endocrine activity, whose adipokine dysregulation and browning have been documented in RCC specifically [11,12]. Peritumoral fat is the subregion of this depot at the tumor surface, where invasion fronts, desmoplasia, and immune infiltration concentrate. The whole perirenal compartment is the tissue at issue in surgical-plane questions such as APF, whereas grade is a property of the tumor and is addressed by fat features only indirectly; Supplementary Table S9 tabulates which compartment each included study sampled. The chain from this biology to CT texture is plausible but indirect: peritumoral inflammation and edema are associated with increased fat attenuation and stranding, the visual findings the MAP score grades [5]; desmoplastic fibrosis introduces linear soft-tissue septa that increase local gray-level variation, to which texture measures such as NGTDM Busyness are sensitive; heterogeneous adipocyte shrinkage and browning shift the attenuation histogram; and neovascularization adds enhancement heterogeneity, to which zone-based features such as ZoneEntropy respond [12,13]. Each link is biologically plausible and supported by observational correlations rather than experimental demonstration, and no radiomic feature reported here is a direct measure of a histological state.

The Category A/B distinction is the field’s central methodological variable, and applying it as an operational rule to the segmentation pipeline described in each primary Methods section, rather than to the label an author uses, changes which studies can contribute a fat-specific estimate: two studies whose fat-related models enclose the whole tumor yield no fat-only value, and one perirenal study that isolates fat geometrically rather than by attenuation falls into Category B. The APF pool, comprising three explicitly segmented studies and one geometric band, retains its defining property: when the tissue analyzed is the tissue whose behavior is predicted, discrimination estimates agree across studies (I^2^ = 0%), in sharp contrast to the grade pool (I^2^ = 93%), where fat features must reflect tumor-intrinsic biology at one remove and where ring constructs of varying thickness sample varying mixtures of fat, parenchyma, and interface. Segmentation variation is thus not a nuisance covariate but a candidate explanation for the field’s heterogeneity, and the per-study ROI inventory of Supplementary Table S9 is intended as a foundation for the uniform reporting the field needs. We would, however, resist reading the exploratory Category subgroup comparison (three studies per arm, *p* = 0.34) as evidence for or against either approach. For APF, the pooled estimate was also materially unchanged in the post hoc analysis restricted to the three Category A studies, although the wider interval and absence of a poolable Category B comparator prevent comparative inference between ROI approaches.

Why is APF prediction the consistent signal? Three candidate explanations, offered as preliminary hypotheses rather than established mechanisms, converge. The target-tissue alignment hypothesis is as follows: APF is a property of the fat itself, an inflammatory–fibrotic state of the perirenal compartment, so its imaging correlates may need no inferential detour through tumor biology. The methodological hypothesis is that all four studies sample the perirenal compartment using comparatively homogeneous pipelines and mostly logistic models. The outcome hypothesis is as follows: the adhesion phenotype has a macroscopic imaging substrate (stranding and thickening) already exploited by the MAP score, so radiomics may be quantifying a signal whose visual correlate is established rather than searching for a subtle one. Against these stand equally concrete cautions: four studies, uniformly HIGH risk of bias; two without independent validation; and one whose reference standard is itself an imaging score, with the circularity that implies. The certainty rating of LOW reflects exactly this balance.

The incremental-value finding deserves the most careful statement, because both its direction and its size matter. Unweighted rank tests ignore sample size and within-study correlation, and a pair assembled across different validation cohorts, tiers, or classifiers does not estimate an incremental effect at all; the analysis reported here is therefore restricted to nested pairs, with exact paired statistics used wherever the primary studies provide them. It yields a positive, direction-stable, but small pooled increment whose governing interval includes zero. An improvement of this size and precision does not establish clinical utility: discrimination gains translate into better decisions only through calibrated risk estimates evaluated for net benefit at clinically chosen thresholds [59,60], and AUC differences in particular are an insensitive and indirect summary of clinical value [33]. No included study reported decision-curve analysis, and only three reported any numeric calibration statistic. A miscalibrated model deployed on discrimination evidence alone could push patients toward radical rather than nephron-sparing surgery, which is the central clinical risk for this application. The combined-model finding is therefore best read as a descriptive pattern consistent with some non-redundant information in the fat compartment of these datasets, echoed in other malignancies where peritumoral adipose features improved prediction [14,15,16,61,62], while the pooled estimate itself remains consistent with no true increment and provides no grounds for deploying combined models. Commentators have argued that image-derived models could eventually serve as a substitute for molecular profiling when tissue is unavailable [63]. The four radiogenomic analyses synthesized here are retrospective correlations within a single public cohort and do not support any inference about substituting imaging for molecular profiling; they are hypothesis-generating only.

Where could these findings meet clinical practice? Current preoperative assessment relies on conventional CT staging, nephrometry scores for surgical complexity (RENAL, PADUA) [64,65], and, specifically for adherent fat, the MAP score [5], which has been validated across surgical approaches and institutions [66,67]. APF is the endpoint with the most consistent estimates, and fat radiomics requires no additional image acquisition, which makes it the most plausible candidate for prospective evaluation within that pathway. Whether such a model would add anything to the MAP score is untested: no included study compared a fat radiomics model with the MAP score in the same patients, three of the four APF studies predict surgically confirmed adhesion, and the fourth predicts the MAP score itself. No model synthesized here should currently guide treatment decisions. This evidence defines a prospective evaluation pathway alongside the MAP score, with grade and stage models remaining adjuncts to, not substitutes for, biopsy and conventional staging.

Translation faces practical obstacles that this literature has yet to address. No study included phantom calibration; only two acquired imaging at multiple time points; no harmonization method such as ComBat was applied across scanners; fat-specific ROI definitions varied from 1 mm rings to whole-compartment masks, with study-specific attenuation windows including −150 to −50 HU, −190 to −10 HU, and −500 to −20 HU; and 88% of cohorts came from one country, leaving generalizability to other populations, scanners, and protocols unknown. The requirements for clinical readiness follow directly: consensus fat ROI definitions with reported attenuation windows, IBSI-compliant pipelines [9], external validation outside the originating region, and calibration plus net-benefit reporting alongside discrimination.

Several limitations warrant emphasis. All 25 studies were retrospective surgical cohorts, a design that both invites selection bias (patients selected for surgery differ systematically from the full spectrum of renal masses) and precludes any prospective assessment of the models’ behavior in intended-use populations. Seventy-two percent carried HIGH PROBAST risk, driven by analysis-domain concerns. Outcome definitions varied within families (WHO/ISUP versus Fuhrman grading; pT stage versus pT3a upstaging versus fat invasion), and histology mixes varied across studies (16 of 25 ccRCC-only), both contributing to heterogeneity and indirectness. Several pools share source patients via public datasets (e.g., TCGA-KIRC in the grade pool), and although omission sensitivity analyses were stable, patient-level overlap cannot be ruled out (Supplementary Table S12). The Category classification and the nested delta-AUC framework were not specified in the registered protocol; each was fixed before the analyses it governs but after descriptive results had been seen. The search could not include Embase, and the resulting risk is quantified rather than dismissed: searches from inception through 2014 identified no eligible earlier study, all 38 full-text-assessed studies were indexed in at least three of the accessible sources, and multi-database searching with citation chasing achieves high recall in empirical studies [68,69], although no combination guarantees completeness and Embase remains a genuine gap. Finally, the search update through August 2026 identified two eligible studies published after the search date: a peritumoral-fat radiomics model predicting stage and grade across three cohorts, from our own group [70], and an intratumoral-plus-peritumoral ensemble for pathological upstaging [71]. Both fall outside the registered search window and are not pooled here; they are identified so that readers can locate them and would be assessed on the same criteria in any update of this review.

Despite these constraints, this review establishes an evidence-based baseline for fat radiomics in RCC: a field-wide map of ROI constructs, evidence consistent with the most reproducible signal lying where fat itself is the pathology, a quantification of how small and uncertain the currently estimable incremental value is, and a concrete methodological agenda. For researchers, that agenda is explicit fat segmentation with reported attenuation windows, event-sufficient samples, independent external validation, calibration and decision-curve reporting, and nested model comparisons with paired statistics so that future incremental claims can be estimated rather than assumed.

## 5. Conclusions

CT-based radiomics of peritumoral and perirenal fat in renal cell carcinoma has been studied in 25 retrospective cohorts, and none of these studies support current clinical use. The most consistent evidence concerns preoperative prediction of adherent perinephric fat, where discrimination estimates agree across studies and remain valid after restricting to surgically confirmed reference standards, at LOW certainty owing to uniformly high risk of bias. Combined fat-plus-tumor models were numerically higher than tumor-only models in most exploratory comparisons, but the formally estimable increment is small, statistically indistinguishable from zero under the governing interval, assumption-dependent, and of unestablished clinical utility; fat-only models showed no evidence of outperforming or differing from intratumoral models for tumor-intrinsic endpoints, and grade prediction in particular remains too heterogeneous to support any generalizable performance claim. Whether fat radiomics adds to existing preoperative assessment is not established by these data, and no included study compared it with the MAP score, nephrometry scoring, or conventional CT staging in the same patients. Surgical planning for adherent fat is the setting closest to evaluation, and the field must first deliver standardized fat segmentation, calibration, and net-benefit evidence and prospective multicenter validation.

## Figures and Tables

**Table 1:** Characteristics of the 25 included studies on CT-based peritumoral and perirenal fat radiomics in renal cell carcinoma (n = 10,761 patients).

Author, Year	Country	Design	n	Subtype	Outcome	Cat.	Fat ROI	Seg.	ML	Fat-Model Val.	Fat AUC (95% CI)	Combined AUC (95% CI)
Chen 2025 [34]	CHN	MC	513	ccRCC	Grade	B	1–5 mm tumor-inclusive dilated masks + habitat subregions e	Manual	LightGBM/SVM	Extern	-NA e	0.852 (0.725–0.979) c
Ding 2025 [35]	CHN	MC	460	Renal neoplasm	APF	B f	5 mm band around renal contour (kidney U-Net + manual gross cleanup)	Semi-auto	Logistic regression	Extern	0.802 (0.672–0.933)	0.805 (0.676–0.935) b
Gill 2019 [36]	USA	SC	83	ccRCC	Grade	A	Juxtatumoral perinephric fat, dilation with fat-only cleanup	Manual	Univariate	Appar	0.746 (0.63–0.86)	NR
Huang 2026 [37]	CHN	MC	323	ccRCC	Stage + Radiogenomics	B	3 mm VOI_peri	Manual	SVM/MLP	Intern	0.79 (0.63–0.94)	0.80 (0.71–0.88)
Khene 2018 [38]	FRA	SC	70	Mixed	APF	A	Perinephric fat manual contour, 3 slices	Manual	Logistic regression	Appar	0.82 (0.65–0.86) d	NR
Li J 2026 [39]	CHN	MC	627	ccRCC	Ki-67	B	1–5 mm ring-only shells	Manual	EXT-stacking	Extern	0.742 (0.676–0.808)	0.756 (0.692–0.821) c
Li S 2024 [40]	CHN	MC	614	ccRCC	Grade	A	Whole-kidney PRAT, deep-learning segmentation (6 voxels)	Auto	SVM ensemble	Extern	0.573 (0.488–0.656)	0.666 (0.583–0.743) g
Li S 2026 [41]	CHN	MC	1661	ccRCC	Stage + Radiogenomics	B	5 mm dilation ring	Semi-auto	LR/SVM/RF + CNN	Extern	0.659 (0.554–0.763)	0.707 (0.588–0.826)
Li X 2024 [42]	CHN	MC	395	ccRCC	Grade/Survival	B	3–5 mm tumor-inclusive merged ROI	Manual	LASSO LR	Extern	NA e	0.90 (0.80–0.99) e
Li X 2025 [43]	CHN	MC	447	ccRCC	Recurrence	B	3–5 mm tumor-inclusive merged ROI	Manual	LASSO Cox	Extern	NA e	C-index 0.952 (0.887–1.000) d,e
Liu 2023 [44]	CHN	SC	131	RCC all	Stage	B	3 mm outer half-ring into perirenal fat space	Semi-auto	CatBoost	Intern	0.926 (0.796–0.985)	0.897 (0.758–0.971)
Luo 2022 [45]	CHN	SC	177	ccRCC	Grade	A	Tumor + 2–6 mm fat-isolated rim (tumor-inclusive union) e	Manual	RF	CV	NA e	0.86 (CI NR) a,e
Ma T 2022 [46]	CHN	SC	220	RCC mixed	APF	A	Manual perinephric fat area, 3 slices	Manual	Logistic regression	Intern	0.956 (0.905–0.999) d	0.949 (0.901–0.997) b
Ma Y 2022 [47]	CHN	SC	370	ccRCC	Grade	A	Fat-facing sector of 2 mm peritumoral shell	Manual	Logistic regression	Intern	0.789 (0.704–0.874)	0.804 (0.720–0.888)
Mei 2025 [48]	CHN	MC	596 h	Renal neoplasm	APF	A	Perirenal fat, HU threshold (−500 to −20) within kidney dilation	Auto	LASSO	Extern h	0.890 (0.818–0.952) h	NR
Triantafyllou 2026 [49]	GRC	MC	354	RCC mixed	Subtype	B	0–9 mm concentric zones	Pre-existing	SVM	NR	NA e	NR
Wang 2026 [50]	CHN	SC	251	ccRCC	Stage + Radiogenomics	A	Whole PRAT within Gerota’s fascia (−150 to −50 HU)	Manual	Logistic regression	Extern	0.82 (0.70–0.94)	0.88 (0.79–0.97)
Wu 2024 [51]	CHN	MC	248	ccRCC	Stage (STI)	B	2–6 mm peritumoral rings (4 mm selected)	Manual	Logistic regression	Extern	0.762 (0.676–0.849) i	0.826 (0.733–0.919) b,i
Xia 2025 [52]	CHN	MC	250	ccRCC	Stage	B	5/10 mm tumor-inclusive merged ROI	Manual	MLP	Extern	NA e	0.873 (0.761–0.984) e
Xu 2025 [53]	CHN	MC	503	ccRCC	Grade	B	10 mm peritumoral shell	Manual	Logistic regression	Extern	0.655 (0.628–0.683)	0.706 (0.675–0.737) b
Yang 2025 [54]	CHN	MC	503	ccRCC	Grade + Radiogenomics	B	5 mm PAT expansion	Manual	XGBoost	Extern	0.92 (0.87–0.95)	NR
Yang L 2022 [55]	CHN	SC	126	RCC all	Capsule invasion	B	3 mm 2D marginal band	Manual	FNN	CV	0.80 (CI NR) a	0.77 (CI NR) a
Yuan 2025 [56]	CHN	MC	1516	RCC all	Stage	B	5 mm peritumoral dilation	Manual	Logistic regression	Extern	0.902 (0.854–0.950) j	0.911 (0.867–0.956) j
Zhou J 2026 [57]	CHN	SC	120	RCC all	Metastasis	A	5 mm perirenal fat band, HU threshold (−190 to −10)	Auto	SVM	Intern	0.851 (0.700–1.000) d	0.958 (0.905–0.986) b,c
Zhou Z 2021 [58]	CHN	MC	203	ccRCC	Grade	B	Peritumoral ring straddling the tumor border (5 mm out + 5 mm in) k	Manual	LASSO	Intern	0.848 (0.760–0.936)	0.836 (0.744–0.928)

Footnotes: a No confidence interval reported in the primary study; value shown descriptively and excluded from weighted pooling. b Combined model incorporates clinical variables (fat + clinical or fat + tumor + clinical); excluded from the nested fat-plus-tumor increment analyses. c Combined model incorporates clinical variables and/or ensemble stacking. d Published interval asymmetric or reaching the AUC boundary; where the published upper bound was 1.000, it is shown as 0.999, the value carried into the logit transformation, and logit-scale standard errors were used. e ROI contains the entire tumor, or the feature set combines intratumoral with peritumoral features; no fat-only estimate exists, and such models are treated as combined constructs (Section 2.3). f Category assigned from the segmentation pipeline documented in the study’s supplementary methods. g Combined model also incorporates whole-kidney features. h Reference standard is a radiologist-assigned MAP score ≥ 3 in all cohorts (imaging surrogate, not surgical adhesion); analyzed n = 92 kidneys per external model (596 enrolled across cohorts); first-reported external cohort shown, second external cohort in sensitivity analyses. i Values taken from the study’s electronic Supplementary Material, which reports the peritumoral signature separately from the combined model. j First-reported external cohort; second external cohort in sensitivity analyses. k Ring includes a 5 mm intratumoral rim inside the tumor border. Abbreviations: APF, adherent perinephric fat; Appar, apparent; AUC, area under the receiver operating characteristic curve; Auto, automatic; Cat, category; ccRCC, clear cell renal cell carcinoma; CHN, China; CI, confidence interval; CT, computed tomography; CV, cross-validation; Extern, external; FNN, forward neural network; FRA, France; GRC, Greece; Intern, internal; IPAT, intratumoral and peritumoral adipose tissue; LASSO, Least Absolute Shrinkage and Selection Operator; LightGBM, Light Gradient Boosting Machine; LR, logistic regression; MC, multicenter; ML, machine learning; MLP, multilayer perceptron; NA e, not applicable; NR, not reported; PAT, peritumoral adipose tissue; PRAT, perirenal adipose tissue; PRF, perirenal fat; PTV, peritumoral volume; RCC, renal cell carcinoma; RF, random forest; ROI, region of interest; SC, single-center; Seg, segmentation; Semi, semi-automatic; SVM, support vector machine; TotalSeg, TotalSegmentator; USA, United States of America; Val, validation; VOI, volume of interest; WHO/ISUP, World Health Organization/International Society of Urological Pathology; XGBoost, Extreme Gradient Boosting.

**Table 2:** Pooled meta-analytic results, sensitivity analyses, and GRADE certainty (REML with Hartung–Knapp intervals).

Outcome	k	Pooled Estimate (95% CI)	95% PI	I^2^ (%)	External-Only	Low-Risk-Only	GRADE Certainty
Grade prediction (fat-only AUC)	6	0.772 (0.599–0.884)	0.298–0.964	93%	0.752 (0.150–0.981), k = 3	0.809 (0.303–0.976), k = 3	VERY LOW
Stage prediction (fat-only AUC)	6	0.813 (0.681–0.899)	0.460–0.957	72%	0.797 (0.565–0.922), k = 4	0.810 (0.346–0.972), k = 3	LOW
APF prediction (fat-only AUC)	4	0.848 (0.738–0.917)	0.738–0.917	0%	n/a (k < 3)	n/a (all HIGH RoB); surgical-reference-only 0.826 (0.615–0.934), k = 3	LOW
Combined vs tumor, stage family (nested delta-AUC)	5	+0.017 (−0.008 to +0.042) at r = 0.5	n/a (τ^2^ = 0)	0%	-	-	LOW
Combined vs fat, stage family (nested delta-AUC)	5	+0.014 (−0.026 to +0.054) at r = 0.5	n/a (τ^2^ = 0)	0%	-	-	LOW
Fat vs tumor, stage family (delta-AUC)	5	+0.007 (−0.056 to +0.070) at r = 0.5	n/a (τ^2^ = 0)	0%	-	-	LOW
Fat vs tumor, grade family (delta-AUC)	4	−0.030 (−0.152 to +0.091) at r = 0.5	−0.276 to +0.215	80%	-	-	VERY LOW

Footnotes: delta-AUC pools use nested within-study pairs on the raw AUC scale, with the within-study correlation r varied over 0–0.90; estimates are shown at r = 0.5, with the governing modified Hartung–Knapp interval, and inference is judged across the whole grid (Section 3.6). Prediction intervals are reported only where between-study variance was estimated to be nonzero; in the three pools with τ^2^ = 0, the prediction interval is indistinguishable from the confidence interval and is not reported. For the AUC pools, modified Hartung–Knapp intervals govern all subsets with k ≤ 5 and are the values shown in the sensitivity columns. The grade-family combined-versus-tumor and combined-versus-fat sets contained two nested pairs each, below the prespecified pooling threshold, and are reported descriptively. Sensitivity columns for the incremental rows do not apply. Egger tests are exploratory and confined to the supplement (all pools k < 10). Abbreviations: APF, adherent perinephric fat; AUC, area under the curve; CI, confidence interval; GRADE, Grading of Recommendations Assessment, Development and Evaluation; k, number of studies (pairs for delta-AUC rows); PI, prediction interval; REML, restricted maximum likelihood; RoB, risk of bias.

**Table 3:** Methodological quality assessment of the 25 included studies using PROBAST, RQS, METRICS, and TRIPOD.

Study	D1	D2	D3	D4	Overall	RQS (/36)	RQS (%)	METRICS (%)	METRICS Category	TRIPOD (%)
Chen 2025 [34]	L	L	L	H	H	15a	41.7	84.4	Excellent	75.0
Ding 2025 [35]	L	L	L	H	H	13	36.1	79.4	Good	66.7
Gill 2019 [36]	L	H	L	H	H	3	8.3	38.9	Low	50.0
Huang 2026 [37]	L	L	L	H	H	13	36.1	85.4	Excellent	66.7
Khene 2018 [38]	L	H	L	H	H	3	8.3	41.0	Moderate	50.0
Li J 2026 [39]	L	L	L	L	L	14	38.9	86.5	Excellent	75.0
Li S 2024 [40]	L	L	L	H	H	13	36.1	77.1	Good	75.0
Li S 2026 [41]	L	L	L	L	L	20	55.6	86.9	Excellent	66.7
Li X 2024 [42]	L	L	L	H	H	13	36.1	70.2	Good	41.7
Li X 2025 [43]	L	L	L	H	H	14	38.9	79.6	Good	58.3
Liu 2023 [44]	L	L	L	H	H	9	25.0	64.8	Good	66.7
Luo 2022 [45]	L	L	L	H	H	6	16.7	56.5	Moderate	58.3
Ma T 2022 [46]	L	H	L	H	H	8	22.2	61.0	Good	66.7
Ma Y 2022 [47]	L	L	L	L	L	9	25.0	68.7	Good	83.3
Mei 2025 [48]	L	H	L	H	H	9	25.0	75.8	Good	58.3
Triantafyllou 2026 [49]	L	L	L	H	H	6	16.7	54.2	Moderate	41.7
Wang 2026 [50]	L	L	L	L	L	20	55.6	82.7	Excellent	75.0
Wu 2024 [51]	L	L	L	H	H	13	36.1	76.4	Good	50.0
Xia 2025 [52]	L	L	L	H	H	14	38.9	76.4	Good	50.0
Xu 2025 [53]	L	L	L	L	L	17a	47.2	76.7	Good	83.3
Yang 2025 [54]	L	L	L	L	L	11	30.6	79.3	Good	66.7
Yang L 2022 [55]	L	L	L	H	H	4	11.1	61.4	Good	58.3
Yuan 2025 [56]	L	L	L	L	L	22	61.1	90.4	Excellent	83.3
Zhou J 2026 [57]	L	L	L	H	H	10	27.8	69.1	Good	66.7
Zhou Z 2021 [58]	L	L	L	H	H	8	22.2	72.6	Good	66.7

Footnote: an RQS calibration item scored on the numeric calibration statistics reported in the primary text. Abbreviations: D1, PROBAST Domain 1 (participants); D2, PROBAST Domain 2 (predictors); D3, PROBAST Domain 3 (outcome); D4, PROBAST Domain 4 (analysis); H, high risk of bias; L, low risk of bias; METRICS, METhodological RadiomICs Score; PROBAST, Prediction model Risk Of Bias ASsessment Tool; RQS, Radiomics Quality Score; TRIPOD, Transparent Reporting of a multivariable prediction model for Individual Prognosis Or Diagnosis.

## Data Availability

All data generated and analyzed during this systematic review are included in the article and its Supplementary Materials. Extracted study-level data and meta-analytic datasets are provided in the Supplementary Materials. Analysis scripts are available from the corresponding author upon reasonable request.

## Supplementary Materials

The following supporting information can be downloaded at https://www.mdpi.com/article/10.3390/jcm15176720/s1, Table S1: Search strategies, per-database yields, and verification searches; Table S2: Studies excluded at full text with reasons; Table S3: Radiogenomic pathway synthesis; Table S4: Other outcomes not poolable in meta-analysis; Table S5: PRISMA 2020 checklist; Table S6: Complete extraction dataset; Table S7: METRICS item-level scoring; Table S8: AMSTAR 2 self-appraisal; Table S9: Per-study fat ROI operational definitions and classification rationale; Table S10: Analysis specifications, data-verification record, nested-pair inventory, delta-AUC results, and sensitivity analyses; Table S11: TRIPOD-SRMA checklist; Table S12: Search verification, relative recall, update sweep, and cohort-overlap audit; Figure S1: Forest plot of grade prediction, fat-only pool; Figure S2: Forest plot of stage-family prediction, fat-only pool; Figure S3: Forest plot of adherent perinephric fat prediction; Figure S4: Exploratory Category A versus Category B subgroup analysis, grade pool; Figure S5: Exploratory funnel plots for the fat-only pools; Figure S6: Leave-one-out sensitivity analysis for the fat-only pools; Figure S7: Pooled AUCs across the prespecified sensitivity subsets; Figure S8: Feature convergence heatmap; Figure S9: Feature class distribution; Figure S10: Reported AUC against cohort size; Figure S11: PROBAST domain judgments per study; Figure S12: Per-study Radiomics Quality Score totals; Figure S13: Per-study METRICS scores; Figure S14: TRIPOD item-level adherence; Figure S15: Agreement between RQS and METRICS; Figure S16: Study characteristics summary; Figure S17: Forest plot of adherent perinephric fat prediction restricted to Category A studies.

## Abbreviations

The following abbreviations are used in this manuscript:

APF	Adherent perinephric fat
AUC	Area under the receiver operating characteristic curve
ccRCC	Clear cell renal cell carcinoma
CI	Confidence interval
CT	Computed tomography
ECM	Extracellular matrix
EMT	Epithelial–mesenchymal transition
GRADE	Grading of Recommendations Assessment, Development and Evaluation
HU	Hounsfield unit
IBSI	Image Biomarker Standardization Initiative
MAP	Mayo Adhesive Probability
METRICS	METhodological RadiomICs Score
NGTDM	Neighborhood gray-tone difference matrix
PRF	Perirenal fat
PRISMA	Preferred Reporting Items for Systematic Reviews and Meta-Analyses
PROBAST	Prediction model Risk Of Bias ASsessment Tool
PTF	Peritumoral fat
RCC	Renal cell carcinoma
REML	Restricted maximum likelihood
ROI	Region of interest
RQS	Radiomics Quality Score
SHAP	SHapley Additive exPlanations
TCGA-KIRC	The Cancer Genome Atlas Kidney Renal Clear Cell Carcinoma cohort
TRIPOD	Transparent Reporting of a multivariable prediction model for Individual Prognosis Or Diagnosis
