# Supplementary material for: CT-Based Peritumoral and Perirenal Fat Radiomics in Renal Cell Carcinoma: A Systematic Review and Meta-Analysis of Grade, Stage, and Adherent Perinephric Fat Prediction
Source: J Clin Med. 2026 Aug 29;15(17):6720. doi: 10.3390/jcm15176720 (PMC13566006; doi:10.3390/jcm15176720)

# Supplementary Materials

## CT-Based Peritumoral and Perirenal Fat Radiomics in Renal Cell Carcinoma: A Systematic Review and Meta-Analysis of Grade, Stage, and Adherent Perinephric Fat Prediction

Abdulrahman Al Mopti, Ali H. D. Alshehri and Abdulsalam Alqahtani

Supplementary Materials for manuscript jcm-4512392. All meta-analytic values in this document derive from a single archived analysis run (R 4.3.3, metafor 4.8.0; 2026-08-19) and from the manuscript; the analysis specifications and the data-verification record are given in Table S10. For every pool of five or fewer studies or pairs, the governing 95% CI is the modified Hartung-Knapp (mKH) interval, in which the Hartung-Knapp standard error is truncated below at the standard error of the corresponding standard-normal model and a t quantile with k - 1 degrees of freedom is applied (Table S10.3).

# Table of Contents

**Supplementary Tables**

Table S1. Search strategies, per-database yields, and verification searches

Table S2. Studies excluded at full-text stage with reasons

Table S3. Radiogenomic pathway synthesis

Table S4. Other outcomes not poolable in meta-analysis

Table S5. PRISMA 2020 checklist (provided as a separate Word file: Table_S5_PRISMA_2020_checklist.docx)

Table S6. Complete extraction dataset (provided as a separate Excel file: Table_S6_Full_Extraction.xlsx)

Table S7. METRICS item-level scoring (provided as a separate Excel file: Table_S7_METRICS_Itemlevel.xlsx)

Table S8. AMSTAR 2 critical appraisal

Table S9. Per-study fat ROI operational definitions and classification rationale

Table S10. Analysis specifications, data-verification record, nested-pair inventory, delta-AUC results, and sensitivity analyses

Table S11. TRIPOD-SRMA checklist

Table S12. Search verification, relative recall, update sweep, and cohort-overlap audit

**Supplementary Figures**

Figure S1. Forest plot: grade prediction (fat-only pool, k = 6)

Figure S2. Forest plot: stage-family prediction (fat-only pool, k = 6)

Figure S3. Forest plot: APF prediction (k = 4)

Figure S4. Exploratory subgroup: Category A versus B (grade)

Figure S5. Exploratory funnel plots and Egger regression

Figure S6. Leave-one-out sensitivity
Figure S7. Sensitivity comparison across subsets
Figure S8. Feature convergence heatmap
Figure S9. Feature class distribution
Figure S10. AUC versus sample size (bubble plot)
Figures S11-S15. Quality assessment details (PROBAST, RQS, METRICS, TRIPOD, RQS-METRICS concordance)
Figure S16. Study characteristics summary (nine panels)
Figure S17. APF ROI-construction sensitivity analysis (Category A only)

## Table S1. Search Strategies for Each Database

*Full search strategies across eight databases (PubMed, Scopus, Web of Science, OpenAlex, Semantic Scholar, CrossRef, Europe PMC, IEEE Xplore), executed 2026-03-29. An Embase strategy was written at the search stage but could not be executed (no institutional access). Multiple search strategies were executed per database to maximize sensitivity, combining a focused primary search with broader sensitivity searches. The yield column reports the executed per-database counts taken from the archived search-execution records; the derivation is documented in Table S10. Backward and forward citation chasing of included studies and relevant reviews was also performed and is reported in the other-methods arm of the PRISMA flow (Figure 1 of the main text).*

| Database | Executed records | Search strategy |
|---|---|---|
| **PubMed (MEDLINE)** | 165 | Search 1: (renal cell carcinoma OR RCC OR kidney cancer OR renal neoplasm) AND (radiomics OR radiomic OR texture analysis OR feature extraction OR machine learning OR deep learning) AND (peritumoral fat OR peri-tumoral fat OR perirenal fat OR perinephric fat OR adipose OR fat stranding) AND (CT OR computed tomography)Search 2: (renal cell carcinoma OR RCC OR kidney cancer) AND (radiomics OR radiomic OR texture analysis OR machine learning OR deep learning) AND (peritumoral OR perirenal OR perinephric)Search 3: (renal cell carcinoma OR kidney cancer) AND (perirenal fat OR perinephric fat) AND (CT OR computed tomography) AND (grade OR stage OR invasion) |
| **Scopus** | 50 | TITLE-ABS-KEY((“renal cell carcinoma” OR “RCC” OR “kidney cancer” OR “renal neoplasm*”) AND (radiomic* OR”texture analys*” OR “feature extract*” OR ”machine learning” OR ”deep learning”) AND (”peritumoral fat” OR ”perirenal fat” OR ”perinephric fat” OR adipose OR ”fat stranding”) AND (”CT” OR ”computed tomography”))Sensitivity: TITLE-ABS-KEY((”renal cell carcinoma” OR ”RCC” OR ”kidney cancer”) AND (radiomic OR”texture analys*” OR “machine learning” OR “deep learning”) AND (peritumoral OR perirenal OR perinephric)) |
| **Web of Science** | 56 | TS=((“renal cell carcinoma” OR “RCC” OR “kidney cancer” OR “renal neoplasm*”) AND (radiomic* OR”texture analys*” OR “feature extract*” OR ”machine learning” OR ”deep learning”) AND (”peritumoral fat” OR ”perirenal fat” OR ”perinephric fat” OR adipose OR ”fat stranding”) AND (”CT” OR ”computed tomography”))Sensitivity: TS=((”renal cell carcinoma” OR ”RCC” OR ”kidney cancer”) AND (radiomic OR”texture analys*” OR “machine learning” OR “deep learning”) AND (peritumoral OR perirenal OR perinephric)) |
| **OpenAlex** | 17 | renal cell carcinoma radiomics peritumoral perirenal fat CT computed tomography |
| **Semantic Scholar** | 59 | renal cell carcinoma radiomics peritumoral perirenal fat CT grading staging |
| **CrossRef** | 1,000 | renal cell carcinoma radiomics peritumoral perirenal fat CT grading stagingAlternate: RCC perinephric fat texture analysis computed tomography grade stage adherent |
| **Europe PMC** | 300 | renal cell carcinoma radiomics peritumoral perirenal fat CTBroader: RCC fat radiomics computed tomography grade stageOutcome-specific: renal carcinoma perirenal fat grade stage CT radiomics |
| **IEEE Xplore** | 1 | (“renal cell carcinoma” OR “RCC”) AND (radiomic* OR “texture analysis” OR “machine learning” OR “deep learning”) AND (peritumoral OR perirenal OR perinephric) AND (“computed tomography” OR “CT”) |
| **Programmatic result batches not attributable to a single database** | 162 a | Result batches ingested during the programmatic compilation whose per-database attribution was not archived (footnote a). |
| **TOTAL, databases-and-registers arm** | **1,810** | Before deduplication (1,703 programmatic records + 107 export records). After removal of 669 duplicates (565 exact duplicates at compilation, 95 at the export merge, 6 automatic fuzzy matches, 3 at manual review) and 324 records published before 2015 (the prespecified window), 817 database records underwent title-and-abstract screening. |

| Database | Executed records | Search strategy |
| --- | --- | --- |
| **Citation chasing (other-methods arm)** | 1 | Backward and forward citation chasing of included studies and relevant reviews (Salimi et al. 2026; Broomand Lomer et al. 2025). The single record identified was assessed and excluded (qualitative CT signs without radiomic feature extraction); with it, 818 records were screened in total. |

Footnote a: The programmatic compilation ingested result batches whose source database was not recorded at the time of execution; 162 of the 1,703 imported programmatic records came from batches not itemized in the archived execution record. The programmatic total of 1,703 is doubly corroborated (the archived execution record, and 1,138 unique records plus 565 removed duplicates), but its full per-database split is not recoverable. PRISMA 2020 marks per-database counts as report-if-feasible; the unattributable batch is disclosed rather than redistributed.

**Verification searches.** Two verification exercises accompany the executed strategies: an inception-through-2014 re-run of the strategies on every API-accessible database, which identified no eligible earlier study, and a relative-recall indexing check confirming that all 38 studies assessed at full text were indexed in at least three of the accessible sources. Together with the documented update sweep through August 2026 and the cohort-overlap audit, these are reported in full in Table S12.

## Table S2. Studies Excluded at Full-Text Stage

*Thirteen studies were excluded after full-text assessment. The most common reason was tumor-only radiomics without a fat-specific ROI (nine studies, code E5, including Muellner 2025, excluded post hoc after verification that fat was explicitly removed from the peritumoral ROI using a -30 HU threshold). Three studies used perilesional rings or bounding-box ROIs not representing adipose tissue (E7), and one addressed the wrong outcome (E8). Exclusion reason codes: E5 = no peritumoral or fat-specific radiomics; E7 = peritumoral ROI without fat-specific segmentation; E8 = wrong outcome (no grade/stage/APF/radiogenomic endpoint).*

| Study | Reason for exclusion | Exclusion code | Stage |
|---|---|---|---|
| **Jiao 2025** | Intratumoral radiomics only; no peritumoral or perirenal fat ROI | E5 | Full-text |
| **Xv 2024** | Intratumoral radiomics only; peritumoral ring not analyzed separately | E5 | Full-text |
| **Alhussaini 2024** | Intratumoral subregion heterogeneity; no fat-specific features | E5 | Full-text |
| **Feng 2024** | Peritumoral ROI is generic ring without fat tissue involvement | E7 | Full-text |
| **Lu 2024** | Intratumoral radiomics only; no peritumoral component | E5 | Full-text |
| **Yang H 2024** | Automatic texture analysis of tumor only; no fat segmentation | E5 | Full-text |
| **Demirjian 2022** | Intratumoral radiomics only; no peritumoral or fat analysis | E5 | Full-text |
| **Yin 2022** | Peritumoral ROI is generic ring; fat not specifically targeted | E7 | Full-text |
| **Shu 2019** | Intratumoral radiomics only; no fat or peritumoral features | E5 | Full-text |
| **Tao 2026** | Intratumoral habitat radiomics (K-means clustering within tumor); no fat | E5 | Full-text |
| **Wang R 2021** | Bounding box approach; ROI does not represent adipose tissue | E7 | Full-text |
| **Moldovanu 2021** | Wrong outcome; no grade/stage/APF/radiogenomic endpoint reported | E8 | Full-text |
| **Muellner 2025** | Fat explicitly excluded from peritumoral ROI using -30 HU threshold | E5 | Post-hoc |

## Table S3. Radiogenomic Pathway Synthesis

*Four studies correlated peritumoral/perirenal fat radiomic features with molecular pathways using TCGA-KIRC data; all are exploratory, retrospective correlation analyses and are presented as hypothesis-generating. Pathways recurring in at least two studies were epithelial-mesenchymal transition (EMT, 2/4 studies) and immune/inflammatory signaling (2/4; IL6-JAK-STAT3, lymphocyte-mediated immunity). Extracellular matrix remodeling (collagen binding, Hippo pathway) was reported by one study (Huang 2026). Wang 2026 linked PRAT browning to angiogenesis; Li S 2026 related geometric features to EMT-driven invasion.*

| study | method | database | fat_roi | key_pathways | key_genes | direction | biological_interpretation |
|---|---|---|---|---|---|---|---|
| **Yang 2025** | TCGA correlation analysis | TCGA-KIRC | 5mm PAT expansion | Lymphocyte-mediated immunity, MHC class II, adaptive immune response | DPEP3, THRSP | Peritumoral features correlate with immune microenvironment | Stromal immune infiltration detectable via fat texture; DPEP3 (renal peptidase) and THRSP (lipogenic) link fat metabolism to tumor grade |
| **Huang 2026** | GSEA/KEGG pathway enrichment | TCGA-KIRC | 3mm VOI_peri | ECM-receptor interaction, Hippo pathway, collagen binding, focal adhesion | Collagen genes, YAP/TAZ pathway | Peritumoral features associated with ECM remodeling and mechanotransduction | Peritumoral fat texture reflects desmoplastic reaction and matrix stiffness changes associated with tumor invasion |
| **Wang 2026** | WGCNA + ssGSEA | TCGA-KIRC | PRAT along Gerota fascia | EMT, hedgehog signaling, cAMP signaling, glycolysis | EMT markers, SHH pathway | PRAT radiomic features correlate with mesenchymal transition and metabolic reprogramming | PRAT browning and angiogenesis create detectable texture changes; EMT drives invasion into peritumoral space |
| **Li S 2026** | ssGSEA + pathway analysis | TCGA-KIRC | 5mm dilation + nnU-Net PRF | EMT, IL6-JAK-STAT3, PI3K-Akt, allograft rejection | EMT markers, inflammatory cytokines | Peritumoral+PRF features correlate with inflammatory and invasion pathways | Geometric invasion patterns (Max3DDiameter) reflect EMT-driven tumor extension; inflammatory fat microenvironment detectable via texture |

## Table S4. Other Outcomes Not Poolable in Meta-Analysis

*Per-study detail for the five unique endpoints summarized in Section 3.9 of the manuscript. Each contributes k = 1 and none is poolable; the table records the outcome definition, the fat classification, the reported performance and its validation tier, and the reason the estimate is not carried into any pool.*

| study | outcome | outcome_definition | fat_classification | fat_auc | tumor_auc | combined_auc | validation | n | key_finding | clinical_relevance |
|---|---|---|---|---|---|---|---|---|---|---|
| **Zhou J 2026** | Metastasis | Postoperative metastasis prediction | A (dedicated PRF) | 0.851 (internal test) | 0.849 (entire dataset) | 0.958 (0.905-0.986) b | Internal test (7:3) for single-ROI models | 120 | Combined clinical+PRF+tumor stack exceeds either single-ROI model on the entire dataset (delta +0.109, resubstitution tier); 5 PRF features selected from 1,680 | PRF remodeling may signal systemic spread potential; combined-model claims await held-out evaluation |
| **Li X 2025** | Recurrence | Recurrence-free survival (LASSO Cox) | B (tumor-inclusive merged ROI) | No separable fat model; intratumoral plus peritumoral single-ROI radiomics (region-level combined; 6 features from IPAT) | C-index 0.920 | C-index 0.952 (0.887-1.000) a | External (n = 52; five recurrence events) | 447 | Combined tumor-plus-peritumoral single-ROI features improve survival prediction; Busyness and RunVariance among top features | Peritumoral fat texture may add prognostic value for recurrence beyond tumor morphology, but the external set carries only five events |
| **Yang L 2022** | Capsule invasion | Renal capsule invasion prediction | B (3mm shell) | 0.80 | 0.81 | 0.77 | 5-fold CV | 126 | Unusual: combined model (0.77) WORSE than fat-alone (0.80) or tumor-alone (0.81); possibly noise from feature mixing; no intervals reported | Negative finding: combining fat and tumor features may not always help; capsule invasion may not benefit from radiomics approach |
| **Li J 2026** | Radiogenomics/ Ki-67 | Ki-67 expression level prediction | B (1-5mm shells) | 0.742 (0.676-0.808) | 0.787 c | 0.756 (0.692-0.821) | External (n = 212) | 627 | Peritumoral ring features predict Ki-67; EXT-stacking with habitat subregions; the same-tier internal comparison favors the peritumoral shell (Table S10, item 9) | Fat/peritumoral features contain proliferation-related information via immune microenvironment and neoangiogenesis signals |
| **Triantafyllou 2026** | Histologic subtype | 5-class RCC subtype classification | B (0-9mm zones) | NR (spatial gradient analysis) | 0.67-0.78 (5-class) | NR | Stratified 70:30 + bootstrap | 354 | Proof-of-concept: peritumoral texture gradients differ by histologic subtype; no predictive model built | Spatial gradient analysis shows fat/peritumoral regions carry subtype-specific information; future work needed for clinical translation |

Footnotes: a Upper confidence limit truncated at the 1.000 boundary (the primary study printed 1.017, which exceeds the C-index bound); logit-scale handling per Methods 2.6 of the main text. b Entire-dataset resubstitution including clinical variables; no held-out combined-model evaluation exists (Table S10). c Internal hold-out estimate (the tumor model has no reported external-tier value); the corrected same-tier internal pair is PERI1mm 0.836

(0.767-0.904) versus INTRA 0.787 (0.701-0.864) (Table S10, item 9). Abbreviations: APF, adherent perinephric fat; AUC, area under the curve; CV, cross-validation; EVS, external validation set; IPAT, internal and peritumoral area of the tumor; NR, not reported; PRF, perirenal fat.

## Tables S5-S7 (separate files)

*Table S5 (PRISMA 2020 Checklist): provided as a separate Word file (Table_S5_PRISMA_2020_checklist.docx) preserving the official PRISMA 2020 template format with all items completed with their section locations in the manuscript.*

Table S6 (Complete Extraction Dataset): provided as a separate Excel file (Table_S6_Full_Extraction.xlsx). Contains all extracted fields for every included study, incorporating the two-pass verification corrections enumerated in Table S10.

Table S7 (METRICS Item-Level Scoring): provided as a separate Excel file (Table_S7_METRICS_Itemlevel.xlsx) containing scores for all 30 METRICS items across the 25 studies with weighted percentage totals and quality category assignments.

---

# Table S8. AMSTAR 2 Critical Appraisal

*Self-appraisal of this systematic review against the 16 items of AMSTAR 2 (Shea BJ et al., BMJ 2017;358:j4008). Seven items are designated critical domains (items 2, 4, 7, 9, 11, 13, 15) and nine are non-critical. Ratings follow standard AMSTAR 2 conventions: Yes = criterion fully met; Partial Yes = most but not all elements present; No = criterion not met. Overall confidence in the results is rated MODERATE on the basis of zero critical flaws, one critical item with a Partial Yes, and two non-critical weaknesses.*

| # | AMSTAR 2 item | Domain | Rating | Evidence / location in this review |
|---|---|---|---|---|
| **1** | Did the research questions and inclusion criteria include the components of PICO? | Non-critical | **Yes** | Population (adults with RCC), Index test (CT radiomics of peritumoral/perirenal fat), Comparator (intratumoral radiomics), and Outcomes (grade, stage, APF) are all specified in Methods 2.3. |
| **2** | Was the protocol registered before commencement, and were significant deviations justified? | Critical | **Yes** | Prospectively registered with PROSPERO (CRD420251150155; Methods 2.1). Deviations from protocol are transparently reported in Methods 2.7 (bivariate model, meta-regression, and CLAIM checklist not feasible; post-hoc Category A/B classification justified in Methods 2.3); protocol amendments were prespecified before the corresponding analyses were run and are documented with their prespecification records (Methods 2.7; Table S10). |
| **3** | Did the review authors explain their selection of study designs for inclusion? | Non-critical | **Yes** | Methods 2.3 restricts inclusion to original research reporting discrimination metrics for grade, stage, APF, or radiogenomic endpoints, which corresponds to the prediction-model study design appropriate to the research question. |
| **4** | Did the review authors use a comprehensive literature search strategy? | Critical | **Partial Yes** | Eight databases searched with full Boolean strategies and executed per-database yields (Table S1); forward and backward citation chasing performed; the 2015 lower date bound justified and tested empirically (an inception-through-2014 re-run of the strategies identified no eligible earlier study); no language restrictions. An Embase strategy was written at the search stage but could not be executed (no institutional access and no mediated route); the resulting risk is quantified rather than dismissed through the verification searches and the relative-recall indexing check (all 38 full-text-assessed studies indexed in at least three accessible sources; Table S12). Grey literature and clinical trial registries were not searched. |
| **5** | Was study selection performed in | Non-critical | **Yes** | Two reviewers independently screened |

| # | AMSTAR 2 item | Domain | Rating | Evidence / location in this review |
|---|---|---|---|---|
| | duplicate? | | | titles/abstracts and full texts, with a third reviewer resolving disagreements (Methods 2.4). |
| **6** | Was data extraction performed in duplicate? | Non-critical | **Yes** | One reviewer extracted data and a second independently verified all extracted values, with discrepancies adjudicated by consensus (Methods 2.4); every value entering an analysis was verified in two independent passes against the primary full texts (Table S10). |
| **7** | Did the review authors provide a list of excluded studies and justify the exclusions? | Critical | **Yes** | All 13 full-text exclusions are listed with specific reasons and exclusion codes in Table S2. |
| **8** | Did the review authors describe the included studies in adequate detail? | Non-critical | **Partial Yes** | Table 1 reports country, design, sample size, histological subtype, outcome, fat ROI classification, shell thickness, segmentation method, ML algorithm, validation strategy, and discrimination metrics for all 25 studies; per-study ROI operational definitions are tabulated in Table S9. Granular population characteristics of primary studies (age, sex distribution, inclusion/exclusion criteria) are not uniformly tabulated. |
| **9** | Did the review authors use a satisfactory technique for assessing risk of bias in individual studies? | Critical | **Yes** | PROBAST was applied as the primary tool for prediction-model studies and complemented by RQS, METRICS, and TRIPOD (Methods 2.5); item-level results are provided in Table 3 and Figure 4. |
| **10** | Did the review authors report on the sources of funding of the studies included in the review? | Non-critical | **No** | Funding sources of the individual included studies were not extracted or tabulated. TRIPOD reporting of funding/COI in primary studies was assessed but per-study funding sources are not reported. |
| **11** | Were appropriate statistical methods used for the meta-analysis? | Critical | **Yes** | Random-effects meta-analysis on the logit-AUC scale with the REML heterogeneity estimator and Hartung-Knapp intervals as the primary model, prespecified in the protocol amendment before the analysis run; DerSimonian-Laird and Paule-Mandel estimators reported as sensitivity analyses. Heterogeneity quantified with $I^2$, $\tau^2$, Cochran's Q, and 95% prediction intervals; for pools of five or fewer studies the modified Hartung-Knapp interval governs, in which the Hartung-Knapp standard error is truncated below at the standard error of the corresponding standard-normal model and a t quantile with k - 1 degrees of freedom is applied. Incremental value estimated under the prespecified nested delta-AUC framework restricted to nested within-study pairs at matched validation tiers, with exact paired statistics where the |

| # | AMSTAR 2 item | Domain | Rating | Evidence / location in this review |
|---|---|---|---|---|
| | | | | primary studies report them and a within-study correlation grid otherwise (Methods 2.6; prespecification records and full pair inventory in Table S10). |
| **12** | Did the review authors assess the potential impact of risk of bias on meta-analytic results? | Non-critical | **Yes** | Prespecified sensitivity analyses restricted the pools to PROBAST low-risk studies, externally validated studies, and studies with n >= 200 (Sections 3.3-3.5; Table 2). Leave-one-out influence analysis was also performed. |
| **13** | Did the review authors account for risk of bias when interpreting the review findings? | Critical | **Yes** | GRADE certainty was explicitly downgraded for risk of bias in all three outcome pools (Figure 5); limitations of retrospective design, geographic concentration, and PROBAST high-risk ratings are discussed in Section 4. |
| **14** | Did the review authors provide a satisfactory explanation for heterogeneity observed? | Non-critical | **Yes** | Heterogeneity is quantified per outcome ($I^2$ 93%, 72%, and 0% for grade, stage, and APF respectively) and mechanistically explained through the Category A/B ROI classification; leave-one-out influence identified drivers (Yang 2025 for grade; Yuan 2025 for stage). |
| **15** | Was publication bias investigated and its likely impact discussed? | Critical | **Yes** | No inferential small-study-effect tests were used because every pool contained fewer than ten studies; reporting bias was assessed qualitatively per the prespecified policy (Methods 2.6). Exploratory Egger regression (grade $p = 0.169$; stage $p = 0.332$; APF $p = 0.409$) and funnel plots are confined to the supplement (Figure S5) and carry no inferential claim; the likely impact of reporting bias is discussed alongside the certainty ratings. |
| **16** | Did the review authors report potential sources of conflict of interest and funding for the review? | Non-critical | **Yes** | Funding source declared (Najran University Deanship of Graduate Studies and Scientific Research, Growth Funding Program, grant NU/GP/MRC/14/2384-1) with explicit statement of no funder involvement; authors declared no conflicts of interest (Disclosure paragraph). |

**Overall confidence: MODERATE.** Zero critical flaws; one critical item rated Partial Yes (item 4: Embase strategy written but not executable, grey literature not searched, both examined empirically in Table S12); two non-critical weaknesses (items 8, 10). The review is otherwise methodologically complete against the AMSTAR 2 framework.

## Table S9. Per-study fat region-of-interest (ROI) operational definitions and classification rationale

*Operational definitions applied (Section 2.3 of the main text).* ***Category A (explicit fat segmentation):*** *the pipeline contains a documented adipose-isolation step: attenuation thresholding, a dedicated fat-segmentation algorithm, or manual fat-only delineation.* ***Category B (peritumoral ring):*** *a fixed-distance dilation without an adipose-isolation step, capturing a ring that includes fat together with renal parenchyma and other tissues. An ROI that contains the entire tumor volume is treated as a combined tumor-plus-fat construct, never as a fat or peritumoral estimate; a model whose feature set combines intratumoral with peritumoral features is likewise treated as combined. Summaries below derive from verbatim Methods quotations collected in two independent blinded extraction passes with consensus adjudication; three classifications requiring adjudication are marked (footnotes a, b, c).*

| Study | Region sampled | ROI construction | Adipose-isolation step | Shell or band thickness | Includes parenchyma or tumor? | Cat. | Rationale |
|---|---|---|---|---|---|---|---|
| **Chen 2025** | Peritumoral interface | Two radiologists manually delineated the entire tumor in ITK-SNAP (contour excludes perirenal and sinus fat); the original ROI mask was then systematically expanded at radial distances of 1, 3, and 5 mm using a mask-filling toolkit. | None (window width 300 / level 25 is display preprocessing, not adipose segmentation) | 1, 3, and 5 mm expansions; 1 mm performed best | Yes; the dilated masks are tumor-inclusive, and the peritumoral signatures combine intratumoral with peritumoral features by design | B a | Fixed-distance dilation without adipose isolation; tumor-inclusive masks with combined intra- plus peritumoral features; no fat-only model; classification determined by the operational criteria in Section 2.3. |
| **Ding 2025** | Whole perirenal compartment | Kidney segmented by 3D U-Net deep learning; perirenal fat obtained as a band extending 5 mm outward from the renal contour on non-contrast CT, with manual modifications (gross cleanup); construction detailed in the retrieved supplementary methods. | None; geometric band with manual gross cleanup, no HU threshold and no voxel-level adipose isolation (per retrieved supplement) | 5 mm outward from the renal contour | Kidney excluded by construction; non-fat tissue within the band (vessels, fascia) not removed voxel-wise | B b | Category assigned from the documented segmentation pipeline: the study's supplementary methods document a geometric band, not voxel-level adipose isolation. |
| **Gill 2019** | Peritumoral interface (juxtatumoral perinephric fat, JPF) | Tumor-ROI dilation and subtraction in Synapse 3D on the nephrographic phase: dilation of 5 pixels on average from the tumor minus a 1-pixel dilation; all non-perinephric-fat voxels (renal parenchyma, bowel, liver, vasculature, lymph nodes, visceral and muscular tissue) manually deleted so the ROI contained only the JPF. | Manual; explicit deletion of every non-adipose voxel (no HU threshold) | Approximately 4-pixel shell (5-pixel minus 1-pixel dilation); millimetre value not stated | No; parenchyma and tumor removed by the manual cleanup | A | Explicit manual adipose isolation follows the geometric dilation. |
| **Huang 2026** | Peritumoral interface | Whole tumor manually delineated slice by slice by Radiologist A and validated by Radiologist B; peritumoral region (VOI_peri) generated automatically by the software as a 3-mm margin around the tumor (3D Slicer). | None | 3 mm | Tumor excluded (VOI_peri is separate); no tissue-exclusion step described, so parenchyma and sinus tissue enter the shell | B | Fixed 3-mm software dilation of the tumor contour with no adipose-isolation step. |

| Study | Region sampled | ROI construction | Adipose-isolation step | Shell or band thickness | Includes parenchyma or tumor? | Cat. | Rationale |
|---|---|---|---|---|---|---|---|
| **Khene 2018** | Whole perirenal compartment (2D; 3 axial slices, ipsilateral kidney) | Perinephric fat area manually contoured on three axial images (renal hilum, first and last sections with visible kidney tissue); contours placed slightly within the fat borders to exclude volume averaging from adjacent parenchyma. | Manual fat-only contouring (no HU threshold; intensity normalization to mu +/- 3 SD is not a fat cut-off) | None; direct 2D contour of the fat compartment | No; contours drawn inside the fat borders specifically to exclude parenchyma | A | Direct fat-only delineation that explicitly excludes renal parenchyma. |
| **Li J 2026** | Peritumoral interface | Manual tumor segmentation (ITK-SNAP); the original mask was expanded into three concentric ring-only peritumoral shells at radial distances of 1, 3, and 5 mm from the tumor margin, each modeled separately (PERI1mm/PERI3mm/PERI5mm). | None (window width 300 / level 40 is image standardization) | 1, 3, and 5 mm concentric shells; 1 mm best-performing | Tumor excluded from the shells; adjacent parenchyma and sinus tissue included, no tissue-type exclusion | B | Concentric fixed-distance shells around the tumor with no adipose-isolation step. |
| **Li S 2024** | Whole perirenal compartment | TransUNet deep-learning segmentation of tumor, kidney, and visceral adipose tissue (VAT; train-while-annotation, radiologist-corrected); PRAT defined by an expansion-erosion algorithm as the VAT voxels within 6 voxels around the kidney, eliminating the VOI outside this range. | Dedicated deep-learning VAT segmentation (no HU window stated in the main text) | 6 voxels around the kidney; millimetre equivalent not reported | No; ROI restricted to segmented VAT voxels | A | Adipose isolation by dedicated VAT segmentation precedes the fixed-distance band. |
| **Li S 2026** | Peritumoral interface | Kidneys, tumors, and perirenal adipose tissue segmented with a KiTS23-pretrained nnU-Net and manually refined; the peritumoral radiomics region defined solely as a three-dimensional 5-mm morphological dilation of the tumor mask (3-, 5-, and 7-mm rings compared; 5 mm selected). | None for the peritumoral ROI; no stated intersection with the perirenal fat mask (the 40/400 HU window is display input for the deep-learning classifier) | 5 mm (3 and 7 mm compared) | Parenchyma and sinus structures within the ring; no exclusion stated | B | Fixed 5-mm dilation of the tumor mask; the separately available fat mask was not used to constrain the ring. |
| **Li X 2024** | Peritumoral interface | Manually delineated internal tumor area (IAT) automatically extended by 3 mm and 5 mm; the PAT ROIs retain the entire internal tumor mask plus the surrounding rim (segmentation protocol in Appendix S3, not available for verification). | None | 3 and 5 mm; ROI = tumor plus rim | Yes; entire tumor plus adjacent parenchyma, sinus, and fat | B | Tumor-inclusive merged ROI without adipose isolation; no fat-only estimate exists. |
| **Li X 2025** | Peritumoral interface | Automated segmentation extending from the tumor core to the peritumoral region; IPAT ROIs comprise the internal tumor volume plus 3-mm or 5-mm expansions (segmentation protocol in Appendix S2, not available for verification). | None described in the accessible text | 3 and 5 mm expansions | Yes; whole tumor plus whatever tissue lies within 3/5 mm of the border | B | Automated fixed-distance expansion never separated from the intratumoral volume. |
| **Liu 2023** | Peritumoral interface | Kidney and mass semi-automatically segmented (U-Net with ITK-SNAP | None; rings generated purely geometrically from | 3 mm (Model 4) and 5 mm | Parenchyma largely excluded geometrically | B | Geometric half-ring dilation into the perirenal fat space with |

| Study | Region sampled | ROI construction | Adipose-isolation step | Shell or band thickness | Includes parenchyma or tumor? | Cat. | Rationale |
|---|---|---|---|---|---|---|---|
| | | revision); the outer half-ring generated automatically along the mass edge at the outer edge of the kidney, extending into the perirenal fat space; inner (intratumoral) rings and combined models also constructed. | the kidney and mass masks | (Model 6) outer half-rings | (half-ring starts at the outer kidney edge), but ring contents are not adipose-verified; combined Models 7/8 add intratumoral rings | | no adipose-isolation step. |
| **Luo 2022** | Peritumoral interface | Tumor VOI dilated by ~1 cm (anisotropic; dilation stops at bowel, liver, spleen, adrenal, vessels, nodes, viscera, muscle); the loop VOI automatically split at -20 HU into perirenal fat (VOI_loop_fat, < -20 HU) and parenchyma (> -20 HU) with morphological post-processing; fat-bearing analysis ROIs (Fat_d2/d4/d6) unite the entire tumor VOI with the fat rim at 2, 4, or 6 mm. | Automatic HU-threshold split at -20 HU plus post-processing (isolated parts removed, cavities filled) | 2, 4, and 6 mm fat dilations within the ~10 mm loop | Yes for tumor: every fat-bearing analysis ROI contains the entire tumor volume; the fat rim itself excludes parenchyma by threshold | A c | Fat-isolated rim (explicit -20 HU split), but no fat-only model exists; all fat-bearing ROIs are tumor-inclusive unions, so the models are treated as combined constructs because the ROI includes the tumor volume. |
| **Ma T 2022** | Whole perirenal compartment (3 axial slices) | Two radiologists jointly and manually delineated the perinephric fat area on plain CT at the widest renal-vein layer of the affected kidney plus the two adjacent layers (3 slices total), confirmed by consensus. | Manual fat-only contouring; no HU window | None; direct contour on 3 consecutive slices | No by design; the contoured target is the perinephric fat itself | A | Direct manual contouring of the perinephric fat, not a dilation construct. |
| **Ma Y 2022** | Peritumoral interface | Peritumor VOIs automatically expanded 2, 5, and 10 mm outside the lesion contour (AK software); the 2-mm shell manually divided at the neighboring tissue interfaces into peritumor-fat (fat-facing sector) and peritumor-kid (parenchyma-facing sector). | Manual interface subdivision separating the fat-facing sector; no HU threshold | 2 mm for the fat/kid subdivision (whole shells at 2, 5, and 10 mm) | Fat sector excludes the kidney-facing sector by design; adipose purity not threshold-verified; the undivided whole-shell models mix fat and parenchyma | A | Explicit manual fat-directed subdivision of the shell goes beyond a bare fixed-distance dilation (borderline A). |
| **Mei 2025** | Whole perirenal compartment (left and right kidneys modeled separately) | Adipose mask obtained by CT range -500 to -20 HU; nnU-Net v2 kidney masks expanded outward by distance D (derived from the MAP-score L length or taken as a fixed gradient distance; scipy binary_dilation); adipose mask intersected with the perirenal region; connected components < 100 voxels removed, Laplacian smoothing. | HU threshold -500 to -20 within the kidney-dilation region | Not fixed in millimetres; D is patient-variable (MAP L length) or a fixed gradient distance | No; the HU criterion excludes parenchyma and other non-fat tissue | A d | Only threshold-defined adipose voxels enter feature extraction; note the MAP-score reference standard (footnote d). |
| **Triantafyllou 2026** | Peritumoral interface | Primary tumor masks processed in 3D Slicer to generate concentric peritumoral expansions at 3, 6, and 9 mm from the tumor boundary; each zone (0-3, 3-6, 6-9 mm) divided into an intra-renal portion (kidney | None; the extra-renal portions are generic surrounding tissues, never adipose-verified | Three concentric zones: 0-3, 3-6, and 6-9 mm | Yes; the intra-renal zones are renal parenchyma by definition | B | Concentric geometric shells with no adipose-specific step. |

| Study | Region sampled | ROI construction | Adipose-isolation step | Shell or band thickness | Includes parenchyma or tumor? | Cat. | Rationale |
|---|---|---|---|---|---|---|---|
| | | parenchyma) and an extra-renal portion (surrounding tissues). | | | | | |
| **Wang 2026** | Whole perirenal compartment (within Gerota's fascia) | PRAT manually delineated slice by slice along Gerota's fascia on corticomedullary axial images (3D Slicer) with the slide bar set to -150 to -50 HU; sinus fat and peri-adrenal adipose excluded; all VOIs manually modified to remove undesired structures. | HU window -150 to -50 plus manual fat-only contouring | None; whole compartment, no dilation | No; parenchyma excluded by the HU window, sinus and adrenal fat excluded, residual non-fat removed manually | A | Exact adipose HU window combined with fascia-bounded fat-only delineation. |
| **Wu 2024** | Peritumoral interface | Manual tumor ROI automatically indented 2 mm inside the tumor and enlarged 2, 4, 5, and 6 mm outside, yielding four annular peritumoral masks (tumor excluded) and one intratumoral mask; the 4-mm ring fed the selected signature. | None (25 HU is the voxel-intensity bin width for feature discretization, not fat masking) | 2, 4, 5, and 6 mm rings; 4 mm optimal | Parenchyma yes, and at larger dilations adjacent organs (liver, spleen, muscle per the Discussion); tumor excluded from the rings | B | Geometric annular dilations of the tumor contour; fat included only incidentally. |
| **Xia 2025** | Peritumoral interface | Tumor region expanded by 5 mm and 10 mm; manual removal of non-parenchymal areas outside the kidney while retaining the peritumoral fat; the resulting TP5mm/TP10mm ROIs merge the whole tumor with the ring. | None; fat deliberately retained but never isolated | 5 and 10 mm | Yes; entire tumor plus renal parenchyma within the ring (the authors note the 10-mm region includes a large amount of normal renal tissue) | B | Tumor-inclusive merged ROIs without adipose isolation; no fat-only estimate exists. |
| **Xu 2025** | Peritumoral interface | Tumor ROI drawn slice by slice on corticomedullary 5-mm images; the 3D Slicer margin module dilated the contour by 10 mm; non-renal-parenchymal tissue outside the kidney manually erased, retaining only perirenal fat; the peritumoral ROI is the combined ROI minus the tumor ROI. | Partial and manual, extra-renal only; the intra-renal parenchyma is retained; no HU threshold | 10 mm | Parenchyma yes (the intra-renal portion of the shell); tumor excluded by subtraction | B | Fixed 10-mm shell that knowingly retains renal parenchyma; fat is isolated only outside the kidney. |
| **Yang 2025** | Peritumoral interface | Intratumoral region manually segmented as the entire tumor volume (necrotic, cystic, hemorrhagic components included; parenchyma, perirenal fat, and sinus fat excluded); the PAT defined as a uniform 5-mm expansion beyond the tumor boundary capturing transitional tissue. | None | 5 mm uniform expansion | Yes; the shell contains adjacent parenchyma, perirenal fat, and sinus fat (exactly the tissues excluded from the intratumoral ROI) | B | Uniform geometric expansion with explicitly mixed transitional-tissue contents. |
| **Yang L 2022** | Peritumoral interface (2D) | Tumor manually segmented on the single largest cross-sectional slice (Philips Radiomics Tool); an extended area of 3 mm adjacent to the tumor boundary automatically generated by image-processing algorithms for the peritumor features. | None | 3 mm, 2D (single largest axial slice) | Yes; parenchyma and other non-fat tissue by construction, no exclusion step | B | Geometric 3-mm marginal band with no adipose step. |

| Study | Region sampled | ROI construction | Adipose-isolation step | Shell or band thickness | Includes parenchyma or tumor? | Cat. | Rationale |
| --- | --- | --- | --- | --- | --- | --- | --- |
| **Yuan 2025** | Peritumoral interface | Tumor mask dilated by 5 mm (Segment Editor module, 3D Slicer); the peritumor mask is the subtraction of the original mask from the dilated mask. | None | 5 mm | Yes; parenchyma, perinephric fat, sinus fat, or collecting system depending on tumor location; tumor excluded by subtraction | B | Uniform 5-mm dilation that the authors themselves flag as unable to target perinephric-fat signs. |
| **Zhou J 2026** | Whole perirenal compartment (affected side, within 5 mm of the renal boundary) | TotalSegmentator (nnU-Net) kidney segmentation with morphological optimization; Euclidean distance transform from the kidney boundary; fat identified at the standard adipose threshold within 1-, 3-, and 5-mm shells; the affected-side 5-mm PRF ROI used for radiomics. | HU threshold -190 to -10 within the distance shells | 5 mm from the renal boundary (1- and 3-mm masks also generated) | No; only threshold-passing fat voxels enter the ROI | A | Voxel-level adipose thresholding inside the geometric shell. |
| **Zhou Z 2021** | Peritumoral interface | PTV created automatically by a morphological method expanding and shrinking the tumor border by 5 mm in three dimensions uniformly; the segmentation was noninteractive, so the PTV contains normal renal tissue, perinephric fat, sinus fat, or microvessels. | None | 5 mm outward plus 5 mm inward (ring straddles the tumor border) | Yes; parenchyma, fat, and vessels, and the ring includes a 5-mm intratumoral rim inside the tumor border | B e | Morphological ring with no adipose step; the inner 5 mm of the ring is tumor tissue (footnote e). |

**Footnotes.** a. Model composition: Chen 2025's dilated masks contain the entire tumor and its peritumoral signatures combine intratumoral with peritumoral features by explicit design; no fat-only estimate exists, and the models are treated as combined constructs (removed from the fat-only grade pool; Table 1, footnote e of the main text). b. Category assignment: Ding 2025 is classified as Category B on the segmentation pipeline documented in the study's supplementary methods, which describe a geometric 5-mm band around the renal contour with gross manual cleanup rather than voxel-level adipose isolation. c. Model composition: Luo 2022 performs explicit adipose isolation of the rim (automatic -20 HU split), but every fat-bearing analysis ROI unites the entire tumor volume with the fat rim; no fat-only model exists, and the models are treated as combined constructs (removed from the fat-only grade pool; Table 1, footnote e of the main text). d. Mei 2025's reference standard is a radiologist-assigned MAP score of 3 or higher in all cohorts (an imaging surrogate, not surgical adhesion); see Table 1, footnote h of the main text. e. Zhou Z 2021's peritumoral ring includes a 5-mm intratumoral rim inside the tumor border; see Table 1, footnote k of the main text.

Abbreviations: APF, adherent perinephric fat; Cat., category; HU, Hounsfield units; IAT, internal area of the tumor; IPAT, internal and peritumoral area of the tumor; JPF, juxtatumoral perinephric fat; MAP, Mayo Adhesive Probability; PAT, peritumoral area; PRAT, perirenal adipose tissue; PRF, perirenal fat; PTV, peritumoral volume; ROI, region of interest; VAT, visceral adipose tissue; VOI, volume of interest.

## Table S10. Analysis specifications, data-verification record, nested-pair inventory, delta-AUC results, and sensitivity analyses

*This table gives, in full, (1) the analysis specifications fixed before the analysis run; (2) the data-verification record, listing every numerical and classification value corrected against the primary publications, with the submitted value, the verified value, and the primary-source basis; (3) the complete inventory of candidate within-study model pairs with their admissibility verdicts under the prespecified nesting taxonomy; and (4) the pooled delta-AUC results across the prespecified within-study correlation grid.*

**Analysis specifications.** The analysis plan amendment (v1.1) was fixed on 2026-08-17 before the analysis run, and the taxonomy interpretation note (v1.2) was fixed after the nesting review and before the run; both records are archived with the registered protocol. All values reported below derive from a single archived analysis run (2026-08-19; R 4.3.3, metafor 4.8.0). Every value entering an analysis was verified in two independent passes against the primary publications of all 25 included studies (zero inter-pass category disagreements), with targeted retrieval of published supplementary materials. A delta-AUC difference of 0.02 was fixed before analysis as a descriptive reference only. It is not an established minimally important clinical difference, was not used to judge clinical relevance, and the certainty ratings rest on whether the governing interval excluded no difference.

### S10.1. Data-verification record: values corrected against the primary publications

| # | Item | Submitted version (old) | Verified value (new) | Basis |
|---|---|---|---|---|
| **1** | Luo 2022: grade fat-only pool membership | Included in the grade fat-only pool (fat AUC 0.86, cross-validation, no CI reported; pool k = 8) | Removed from the fat-only pool. Every “Fat_dX” ROI is the tumor VOI plus a fat rim, i.e., the entire tumor volume is inside the ROI; the study contains no fat-only model and is treated as a combined/region-union construct | Both verification passes; Luo 2022 Table 3; bright-line rule added to Methods 2.3 (an ROI containing the entire tumor volume is never a fat/peritumoral estimate) |
| **2** | Chen 2025: grade fat-only pool membership | Included in the grade fat-only pool (peritumoral signature, external validation) | Removed from the fat-only pool. Four convergent lines of evidence: Methods verbatim “We combine intra- and peri-tumor features”; no ring-subtraction step described; Figure 2 renders each PeriXmm mask as tumor + margin; feature count 4,424 = one tumor-inclusive mask, not 8,848 = two concatenated sets. Contributes tumor-only and combined models only | Extraction adjudication record; Chen 2025 Methods and Figure 2 |
| **3** | Grade fat-only pool (net effect of 1-2) | k = 8; pooled AUC 0.783 (0.666-0.867), I² = 92% (DerSimonian-Laird) | k = 6 (Ma Y, Yang, Zhou Z, Gill, Li S 2024, Xu); pooled AUC 0.772 (0.599-0.884), I² = 93.4%, 95% PI 0.298-0.964 (REML + Hartung-Knapp) | Submitted Table 2; archived analysis output, section 1 |
| **4** | Ding 2025: fat ROI category | Category A (explicit fat segmentation) | Category B (peritumoral/perirenal ring). The retrieved supplement (Springer MOESM1) documents a geometric 5-mm outward dilation of the renal contour with manual gross cleanup, no HU thresholding and no dedicated fat segmentation. Consequences: study counts | Ding 2025 Supplementary Digital Material 2 (retrieved at revision); extraction adjudication record |

| # | Item | Submitted version (old) | Verified value (new) | Basis |
|---|---|---|---|---|
| | | | become 9 A / 16 B; "all four APF studies used Category A" corrected throughout (APF pool = 3 A + 1 B) | |
| **5** | Wu 2024: combined-vs-tumor pair | Pair "combined 0.762 vs tumor 0.653" in the incremental set | Pair removed as invalid twice over: the tumor value 0.653 does not exist anywhere in the publication (exhaustive text search plus visual reading of the full text), and 0.762 is the ROI4-NP peritumoral-signature external AUC, not the combined model. The true combined model (0.826 external) is peritumoral radscore + clinical, hence non-nested for the fat increment | Extraction adjudication record; Wu 2024 full text and electronic supplementary material |
| **6** | Wu 2024: stage fat-only pool membership | Not in the stage fat-only pool (peritumoral estimate mislabeled as a combined model; no CI in main text) | Added to the stage fat-only pool (k = 5 to 6): peritumoral signature 0.762 (0.676-0.849), external validation; CI recovered from the study's electronic supplement | Archived analysis dataset (stage pool); manuscript Table 1 footnote i; extraction adjudication record |
| **7** | Stage fat-only pool (net effect of 6) | k = 5; pooled AUC 0.830 (0.658-0.925), I² = 79% (DerSimonian-Laird) | k = 6; pooled AUC 0.813 (0.681-0.899), I² = 72.3%, 95% PI 0.460-0.957 (REML + Hartung-Knapp) | Submitted Table 2; archived analysis output, section 1 |
| **8** | Li S 2026: nested-pair cohort composition | Pair assembled from different validation cohorts: fat 0.659 (Cohort I) against tumor 0.664 and combined 0.751 (Cohort IV) | Corrected to the same-cohort trio, Validation Cohort I: fat 0.659, tumor 0.677, combined 0.707; combined-vs-tumor d = +0.030 (exact DeLong p = 0.729) | Extraction adjudication record; archived analysis dataset (combined-versus-tumor, stage) |
| **9** | Li J 2026: fat-model value | Fat external AUC recorded as 0.735 | Corrected to 0.742 (0.676-0.808), the PERI1mm ring-only external estimate; 0.735 is the habitat (tumor-subregion) model's external AUC, not a fat model. Additionally, the submitted fat-vs-tumor pair mixed tiers (fat 0.735 external vs tumor 0.787 internal); corrected to the same-tier internal pair PERI1mm 0.836 (0.767-0.904) vs INTRA 0.787 (0.701-0.864) | Two-pass verification record (model inventory); manuscript Table 1; extraction adjudication record |
| **10** | Huang 2026: nested-pair tier and classifier | Pair mixed both tier and classifier: combined 0.80 external (SVM) vs tumor 0.74 internal (NaiveBayes) | Corrected to the same-tier, same-classifier pair (SVM internal, the authors' designated combined classifier): VOI_mix 0.82 (0.68-0.96) vs fusion base VOI_sub1 0.71 (0.53-0.89), d = +0.11; the base is a habitat subregion (disclosed; sensitivity excluding Huang reported); whole-tumor SVM comparator (VOI_whole 0.68, 0.51-0.85) reported descriptively | Extraction adjudication record (six rows per model, one per classifier); archived analysis dataset (combined-versus-tumor, stage) |
| **11** | Yuan 2025: nested comparator | Combined M-PIT compared against IT 0.887, which overstated the increment | Corrected to the nested comparator M: M-PIT 0.911 vs M 0.895, d = +0.016 (exact DeLong p = 0.09); TCIA cohort (d = -0.013, p = 0.18) in sensitivity analysis | Extraction adjudication record; archived analysis dataset (combined-versus-tumor, stage) |
| **12** | Zhou J 2026: fat-vs-tumor tier | Pair mixed tiers: fat 0.851 (test set) vs tumor 0.849 (entire dataset) | Corrected to the entire-dataset tier carrying the exact DeLong statistic: fat 0.837 vs tumor 0.849 (difference -0.0112, SE 0.0521, p = 0.829); fat-model CI recovered from Table 4 of that study (0.7004-1.0000), removing the last n-only SE approximation | Extraction adjudication record |
| **13** | Two omitted nested pairs restored | Liu 2023 and Zhou Z 2021 combined-vs-tumor pairs absent from the submitted incremental set | Pair-completeness correction: Liu 2023 d = +0.000 (0.897 vs 0.897, exact p = 1.000; internal) and Zhou | Extraction adjudication record; archived analysis dataset (combined-versus-tumor, stage); |

| # | Item | Submitted version (old) | Verified value (new) | Basis |
|---|---|---|---|---|
| | | | Z 2021 sTM-PTV vs TMV d = +0.026 (exact DeLong p = 0.200; internal) added | archived analysis output, section 4 |
| **14** | Fat-vs-tumor median difference | -0.033 reported in the submitted text (the archived submitted analysis output shows -0.009; a transcription-level inconsistency) | -0.009 on the corrected 12-pair descriptive set (median -0.0085; 5/12 fat-better; exploratory Wilcoxon V = 38, p = 1.00) | Submitted text and archived submitted analysis output; archived analysis output, section 5 |
| **15** | Stage Egger p value | 0.613 in the submitted Table 2 | Corrected recomputation on the submitted pool gave 0.432; superseded by the revision k = 6 pool: z = 1.103, p = 0.332. All Egger tests are exploratory and confined to the supplement (every pool k < 10) | Submitted Table 2; archived submitted analysis output; the prespecified Egger policy in the analysis plan amendment; archived analysis output, section 3 |
| **16** | Calibration reporting count | "1/25 (4%)" reported numeric calibration | Item-level recount in three tiers: Tier 1 (curve shown) 10/25 (Zhou J, Ma T, Ding, Li S 2024, Li X 2025, Xia, Chen, Li J, Xu, Yuan); Tier 2 (numeric statistic) 3/25 (Li S 2024 Brier 0.186/0.178; Xu HL chi-square 3.042, P = .932 and 8.304, P = .404; Chen HL 0.920/0.824/0.812, metric-ambiguous); Tier 2 (threshold-only) 1/25 (Wang: HL all p > 0.05, values in an unretrieved ESM); Tier 3 (poolable) 0/25 | Archived analysis output, calibration-tier block; extraction adjudication record |
| **17** | RQS calibration item re-scores | Xu 2025 RQS 16/36; Chen 2025 RQS 14/36 | Xu 2025 17/36; Chen 2025 15/36, after item-level verification of numeric calibration statistics in the primary texts; the median RQS (13/36) is unchanged | Manuscript Table 3 footnote a; archived quality-appraisal dataset |
| **18** | APF pool estimator restatement (no value change) | Pooled AUC 0.849 (0.737-0.919), $I^2$ = 5% (DerSimonian-Laird); GRADE MODERATE | Pooled AUC 0.848 (0.738-0.917), $I^2$ = 0% (REML + Hartung-Knapp); GRADE LOW (prespecified risk-of-bias -2: 4/4 HIGH PROBAST, two studies without independent validation). Values of the four member studies unchanged; Mei reference standard (radiologist-assigned MAP >= 3, an imaging surrogate) newly disclosed as indirectness | Submitted Table 2; archived analysis output, section 1; archived certainty-assessment record |

Notes. The estimator change (DerSimonian-Laird primary to REML primary with Hartung-Knapp intervals) is a declared protocol amendment, prespecified in the amendment before the analysis run; DerSimonian-Laird and Paule-Mandel results are reported as sensitivity analyses (archived analysis output, section 1: grade DL 0.771, PM 0.772; stage DL 0.814, PM 0.812; APF DL 0.849, PM 0.849). The descriptive Wilcoxon pair sets changed from the submitted 14/14/11 to the corrected 12 (fat vs tumor), 15 (combined vs tumor), and 12 (combined vs fat) after the corrections above.

## S10.2. Nested-pair inventory (all candidate within-study pairs, with admissibility verdicts)

Taxonomy (fixed before the analysis run; amendment interpretation note v1.2): **nested-refit** = separate tumor and fat/peritumoral feature sets, combined model built on the union of the two pools, same cohort, tier, and algorithm family, nothing else added (admissible for weighted pooling); **region-union** = a single merged tumor-plus-ring ROI with no separable fat feature set (non-nested, descriptive only); **clinical/other-added** = combined model includes clinical variables, kidney features, or reader input (non-nested for the fat increment, descriptive only); **no-CI** = no confidence interval and no exact paired statistic recoverable (excluded from weighted pooling, retained

descriptively). Variance source: **exact** = study-reported paired DeLong statistic or exact two-sided p; **CI-based** = per-model 95% CIs combined as Var(d) = SE1^2 + SE2^2 - 2r SE1 SE2 over the correlation grid r = 0, 0.25, 0.50, 0.75, 0.90.

### *S10.2a. Combined vs tumor (15 descriptive pairs; 5 nested stage pairs pooled; 2 nested grade pairs below the k >= 3 stopping rule)*

| Study | Family | d | Tier | Verdict | Variance source | Note |
|---|---|---|---|---|---|---|
| **Wang 2026** | Stage | +0.040 | External | Nested-refit (pooled) | CI-based | 0.88 (0.79-0.97) vs 0.84 (0.72-0.95) |
| **Yuan 2025** | Stage | +0.016 | Bi-center external | Nested-refit (pooled) | Exact DeLong (p = 0.09) | M-PIT 0.911 vs M 0.895; comparator corrected (item 11); TCIA in sensitivity |
| **Li S 2026** | Stage | +0.030 | External (Cohort I) | Nested-refit (pooled) | Exact DeLong (p = 0.729) | 0.707 vs 0.677; cohort-mix corrected (item 8) |
| **Liu 2023** | Stage | +0.000 | Internal | Nested-refit (pooled) | CI-based (d = 0; exact p = 1.000 carries no SE) | 0.897 vs 0.897; pair restored (item 13) |
| **Huang 2026** | Stage | +0.110 | Internal (SVM) | Nested-refit (pooled) | CI-based | VOI_mix 0.82 vs habitat base VOI_sub1 0.71; base disclosed; tier/classifier corrected (item 10); sensitivity excluding Huang reported |
| **Ma Y 2022** | Grade | +0.008 | Internal | Nested (descriptive; family k = 2 < 3) | CI-based | Borderline composition (peritumoral pool is the mixed fat-facing shell) |
| **Zhou Z 2021** | Grade | +0.026 | Internal | Nested (descriptive; family k = 2 < 3) | Exact DeLong (p = 0.200) | sTM-PTV vs TMV; pair restored (item 13) |
| **Li X 2024** | Grade | +0.200 | External | Region-union (descriptive) | - | RM3/RM5 merged tumor-plus-ring ROI; no separable fat feature set |
| **Xia 2025** | Stage | +0.043 | External | Region-union (descriptive) | - | RM5/RM10 merged ROI |
| **Zhou J 2026** | Metastasis | +0.109 | Entire dataset | Clinical-added (descriptive) | - | Combined = clinical + fat + tumor stack, no held-out combined evaluation |
| **Chen 2025** | Grade | +0.073 | External | Clinical-added (descriptive) | - | Combined = habitat + peritumoral + clinical |
| **Li J 2026** | Ki-67 | +0.061 | Internal | Clinical-added (descriptive) | - | EXT-stacking fusion includes the clinical model |
| **Xu 2025** | Grade | +0.021 | External | Clinical-added (descriptive) | - | Combined adds age |
| **Yang L 2022** | Capsule invasion | -0.040 | Cross-validation | No-CI (descriptive) | - | No interval reported |
| **Li S 2024** | Grade | -0.047 | External | Other-added (descriptive) | - | Combined adds whole-kidney features |
| **Wu 2024** | Stage (STI) | - | - | No admissible pair | - | Submitted pair invalid (item 5): tumor value nonexistent; combined is peritumoral + clinical |
| **Luo 2022** | Grade | - | - | No admissible pair (region- | - | All Fat_dX ROIs tumor-inclusive; no fat-only model (item 1) |

| Study | Family | d | Tier | Verdict | Variance source | Note |
|---|---|---|---|---|---|---|
| | | | | union) | | |
| **Li X 2025** | Recurrence | - | - | No admissible pair (region-union) | - | Single IPAT merged ROI; C-index outcome |

### *S10.2b. Combined vs fat (12 descriptive pairs; 5 nested stage pairs pooled; 2 nested grade pairs below the stopping rule)*

| Study | Family | d | Tier | Verdict | Variance source | Note |
|---|---|---|---|---|---|---|
| **Wang 2026** | Stage | +0.060 | External | Nested-refit (pooled) | CI-based | 0.88 (0.79-0.97) vs 0.82 (0.70-0.94) |
| **Yuan 2025** | Stage | +0.009 | Bi-center external | Nested-refit (pooled) | Exact DeLong (p = 0.57) | M-PIT vs PIT |
| **Li S 2026** | Stage | +0.048 | External (Cohort I) | Nested-refit (pooled) | CI-based | 0.707 (0.588-0.826) vs 0.659 (0.554-0.763) |
| **Liu 2023** | Stage | -0.029 | Internal | Nested-refit (pooled) | Exact DeLong (p = 0.713) | 0.897 vs 0.926 |
| **Huang 2026** | Stage | +0.080 | Internal (SVM) | Nested-refit (pooled) | CI-based | Fat comparator = VOI_peri SVM per the same-classifier rule; the authors' designated fat classifier (MLP, 0.79) in sensitivity |
| **Ma Y 2022** | Grade | +0.015 | Internal | Nested (descriptive; family k = 2 < 3) | CI-based | 0.804 vs 0.789 |
| **Zhou Z 2021** | Grade | -0.012 | Internal | Nested (descriptive; family k = 2 < 3) | Exact DeLong (p = 0.538) | sTM-PTV vs PTV |
| **Zhou J 2026** | Metastasis | +0.121 | Entire dataset | Clinical-added (descriptive) | - | Clinical in stack, no held-out combined evaluation |
| **Li S 2024** | Grade | +0.093 | External | Other-added (descriptive) | - | Kidney features added |
| **Xu 2025** | Grade | +0.051 | External | Clinical-added (descriptive) | - | Age added |
| **Li J 2026** | Ki-67 | +0.012 | Internal | Clinical-added (descriptive) | - | Clinical in stacking fusion |
| **Yang L 2022** | Capsule invasion | -0.030 | Cross-validation | No-CI (descriptive) | - | No interval reported |

### *S10.2c. Fat vs tumor (12 descriptive pairs; 5 stage pairs and 4 grade pairs pooled)*

| Study | Family | d | Tier | Verdict | Variance source | Note |
|---|---|---|---|---|---|---|
| **Liu 2023** | Stage | +0.029 | Internal | Admissible (pooled, stage) | Exact DeLong (p = 0.7082) | 0.926 vs 0.897 |
| **Xu 2025** | Grade | -0.030 | External | Admissible (pooled, grade) | Exact DeLong (p = 0.413) | Fat 0.655 vs tumor 0.685; family assignment corrected in the final run (initially assigned to the stage pool; identified during verification) |
| **Yuan 2025** | Stage | +0.007 | Bi-center external | Admissible (pooled, stage) | CI-based | PIT 0.902 vs M 0.895; TCIA DeLong p = 0.02 (tumor-better) in sensitivity |
| **Wang 2026** | Stage | -0.020 | External | Admissible (pooled, stage) | CI-based | 0.82 vs 0.84 |
| **Li S 2026** | Stage | -0.018 | External | Admissible | CI-based | 0.659 vs 0.677; same-cohort corrected (item 8) |

| Study | Family | d | Tier | Verdict | Variance source | Note |
|---|---|---|---|---|---|---|
| | | | (Cohort I) | (pooled, stage) | | |
| **Huang 2026** | Stage | +0.080 | Internal (MLP/MLP) | Admissible (pooled, stage) | CI-based | 0.79 vs 0.71, same classifier |
| **Ma Y 2022** | Grade | -0.007 | Internal | Admissible (pooled, grade) | CI-based | 0.789 vs 0.796 |
| **Zhou Z 2021** | Grade | +0.038 | Internal | Admissible (pooled, grade) | Exact DeLong (p = 0.061) | PTV 0.848 vs TMV 0.81 |
| **Li S 2024** | Grade | -0.140 | External | Admissible (pooled, grade) | CI-based | 0.573 vs 0.713 |
| **Li J 2026** | Ki-67 | +0.049 | Internal | Descriptive only (endpoint outside the grade/stage families) | Per-model CIs available | PERI1mm 0.836 vs INTRA 0.787; tier corrected (item 9) |
| **Zhou J 2026** | Metastasis | -0.012 | Entire dataset | Descriptive only (endpoint outside the families) | Exact DeLong (p = 0.829) | 0.837 vs 0.849; tier corrected (item 12) |
| **Yang L 2022** | Capsule invasion | -0.010 | Cross-validation | No-CI, descriptive only (endpoint also outside the families) | - | 0.80 vs 0.81, no intervals |

Note on family membership: an earlier run assigned Xu 2025 (grade endpoint) to the stage-family fat-vs-tumor pool; the discrepancy was identified during verification, and the analysis was corrected and re-run before submission. The final pools are stage k = 5 (Liu, Yuan, Wang, Li S 2026, Huang) and grade k = 4 (Ma Y, Zhou Z, Li S 2024, Xu), exactly as reported in the archived analysis output, section 4. Excluded from every fat-vs-tumor set: Luo 2022 (no fat-only model), Chen 2025 (no fat-only model), and studies without any matched-tier tumor comparator.

## S10.3. Pooled delta-AUC across the correlation grid (all values from the archived analysis output, section 4; modified Hartung-Knapp intervals govern)

Every pool in this section contains five or fewer pairs, so the governing 95% CI for each estimate is the modified Hartung-Knapp (mKH) interval, in which the Hartung-Knapp standard error is truncated below at the standard error of the corresponding standard-normal model and a t quantile with k - 1 degrees of freedom is applied; the unmodified Hartung-Knapp (HK) interval is reported alongside for transparency.

Where the between-study variance was estimated at zero, the prediction interval coincides with the confidence interval of the same model and is not reported separately in the main text (Table 2).

### *S10.3a. Combined vs tumor, stage family (k = 5 nested pairs: Wang, Yuan, Li S 2026, Liu, Huang)*

| r | Pooled dAUC | 95% CI (mKH, governing) | 95% CI (HK) | $\tau^2$ | $I^2$ | 95% PI |
|---|---|---|---|---|---|---|
| **0.00** | +0.017 | -0.009 to +0.042 | +0.007 to +0.027 | 0.00000 | 0% | +0.007 to +0.027 |
| **0.25** | +0.017 | -0.009 to +0.042 | +0.005 to +0.028 | 0.00000 | 0% | +0.005 to +0.028 |
| **0.50** | +0.017 | -0.008 to +0.042 | +0.003 to +0.031 | 0.00000 | 0% | +0.003 to +0.031 |
| **0.75** | +0.018 | -0.007 to +0.042 | -0.001 to +0.036 | 0.00000 | 0% | -0.001 to +0.036 |
| **0.90** | +0.019 | -0.007 to +0.046 | -0.007 to +0.046 | 0.00000 | 0% | -0.007 to +0.046 |

Leave-one-out (r = 0.5): maximum shift omitting Yuan 2025, pooled +0.031. Robustness: direction invariant = TRUE; inference invariant = TRUE under the governing mKH interval, which includes no difference at every r. Grade family: k = 2 nested pairs, below the prespecified stopping rule, descriptive only (Ma Y 2022 +0.008; Zhou Z 2021 sTM-PTV +0.026, exact DeLong p = 0.200).

### *S10.3b. Combined vs fat, stage family (k = 5 nested pairs)*

| r | Pooled dAUC | 95% CI (mKH, governing) | 95% CI (HK) | τ² | I² | 95% PI |
|---|---|---|---|---|---|---|
| **0.00** | +0.011 | -0.030 to +0.053 | -0.010 to +0.032 | 0.00000 | 0% | -0.010 to +0.032 |
| **0.25** | +0.012 | -0.029 to +0.053 | -0.011 to +0.035 | 0.00000 | 0% | -0.011 to +0.035 |
| **0.50** | +0.014 | -0.026 to +0.054 | -0.012 to +0.041 | 0.00000 | 0% | -0.012 to +0.041 |
| **0.75** | +0.020 | -0.020 to +0.060 | -0.013 to +0.053 | 0.00004 | 2% | -0.018 to +0.058 |
| **0.90** | +0.035 | -0.010 to +0.080 | -0.006 to +0.075 | 0.00037 | 28% | -0.032 to +0.102 |

Leave-one-out (r = 0.5): maximum shift omitting Yuan 2025, pooled +0.041. Robustness: direction invariant = TRUE; inference invariant = TRUE (the governing mKH interval includes no difference at every r). Grade family: k = 2, descriptive only (Ma Y +0.015; Zhou Z sTM-PTV vs PTV -0.012, exact DeLong p = 0.538).

### *S10.3c. Fat vs tumor, stage family (k = 5 pairs: Liu, Yuan, Wang, Li S 2026, Huang)*

| r | Pooled dAUC | 95% CI (mKH, governing) | 95% CI (HK) | τ² | I² | 95% PI |
|---|---|---|---|---|---|---|
| **0.00** | +0.009 | -0.076 to +0.093 | -0.023 to +0.040 | 0.00000 | 0% | -0.023 to +0.040 |
| **0.25** | +0.008 | -0.067 to +0.083 | -0.024 to +0.039 | 0.00000 | 0% | -0.024 to +0.039 |
| **0.50** | +0.007 | -0.056 to +0.070 | -0.025 to +0.038 | 0.00000 | 0% | -0.025 to +0.038 |
| **0.75** | +0.006 | -0.039 to +0.052 | -0.026 to +0.038 | 0.00000 | 0% | -0.026 to +0.038 |
| **0.90** | +0.006 | -0.026 to +0.038 | -0.026 to +0.038 | 0.00000 | 0% | -0.026 to +0.038 |

Leave-one-out (r = 0.5): maximum shift omitting Huang 2026, pooled +0.002. Robustness: direction invariant = TRUE; inference invariant = TRUE (the governing mKH interval includes no difference at every r).

### *S10.3d. Fat vs tumor, grade family (k = 4 pairs: Ma Y, Zhou Z, Li S 2024, Xu)*

| r | Pooled dAUC | 95% CI (mKH, governing) | 95% CI (HK) | τ² | I² | 95% PI |
|---|---|---|---|---|---|---|
| **0.00** | -0.024 | -0.141 to +0.093 | -0.140 to +0.093 | 0.00347 | 69% | -0.245 to +0.197 |
| **0.25** | -0.027 | -0.147 to +0.092 | -0.146 to +0.092 | 0.00397 | 74% | -0.260 to +0.206 |
| **0.50** | -0.030 | -0.152 to +0.091 | -0.151 to +0.090 | 0.00453 | 80% | -0.276 to +0.215 |
| **0.75** | -0.034 | -0.157 to +0.090 | -0.156 to +0.089 | 0.00516 | 86% | -0.293 to +0.226 |
| **0.90** | -0.035 | -0.160 to +0.090 | -0.158 to +0.087 | 0.00557 | 92% | -0.303 to +0.232 |

Leave-one-out (r = 0.5): maximum shift omitting Li S 2024, pooled +0.010. Robustness: direction invariant = TRUE; inference invariant = TRUE (the governing mKH interval includes no difference at every r).

### *S10.3e. Exploratory Wilcoxon descriptive sets (corrected; unweighted; labeled exploratory throughout)*

| Comparison | Pairs | First-named better | Median difference | Wilcoxon V | p |
|---|---|---|---|---|---|
| **Fat vs tumor** | 12 | 5/12 | -0.0085 | 38 | 1.0000 |
| **Combined vs tumor (incl. non-nested)** | 15 | 12/15 | +0.0300 | 90 | 0.0219 |
| **Combined vs fat (incl. non-nested)** | 12 | 9/12 | +0.0315 | 64 | 0.0498 |

Certainty for the incremental outcomes follows the prespecified assessment archived with the analysis record: the stage-family combined-versus-tumor, combined-versus-fat, and fat-versus-tumor comparisons are rated LOW, and the grade-family fat-versus-tumor comparison is rated VERY LOW on three serious domains (risk of bias, inconsistency with I² 80%, and imprecision). The stage-family combined-versus-tumor increment is positive and direction-stable, but its governing mKH interval includes no difference at every correlation value.

### *S10.3f. Logit-scale sensitivity analyses (r = 0.5)*

Each pooled delta-AUC analysis was repeated on the logit-AUC scale at r = 0.5 as a sensitivity check on the raw-scale pooling; values verbatim from the archived analysis output, section 4 (governing mKH intervals):

| Comparison | Family | k | Pooled logit-dAUC (mKH 95% CI) | $I^2$ |
|---|---|---|---|---|
| **Combined vs tumor** | Stage | 5 | +0.214 (-0.291 to +0.719) | 0% |
| **Combined vs fat** | Stage | 5 | +0.196 (-0.290 to +0.682) | 0% |
| **Fat vs tumor** | Stage | 5 | +0.048 (-0.434 to +0.529) | 0% |
| **Fat vs tumor** | Grade | 4 | -0.201 (-0.728 to +0.327) | 62% |

The logit-scale results reproduce the raw-scale conclusions in direction and inference: every interval includes no difference.

## S10.4. ROI-construction sensitivity analysis, APF pool

The APF pool contains three Category A studies and one Category B study. A post hoc sensitivity analysis was restricted to the three Category A studies using explicit adipose segmentation and was run using the primary discrimination pipeline without modification: logit-transformed AUCs, standard errors derived from reported 95% confidence intervals, REML random-effects estimation, and a modified Hartung-Knapp interval governing at k = 3. Category B was represented by one study and was therefore not poolable separately.

| Analysis | Studies | k | Pooled AUC | Governing 95% mKH CI | 95% PI | $I^2$ | $\tau^2$ | Q (p) |
|---|---|---|---|---|---|---|---|---|
| **Full APF pool** | Khene 2018; Ma T 2022; Mei 2025; Ding 2025 | 4 | 0.848 | 0.738–0.917 | 0.738–0.917 | 0% | 0.0000 | 3.15 (0.370) |
| **Category A only** | **Khene 2018; Ma T 2022; Mei 2025** | **3** | **0.863** | **0.643–0.956** | **0.547–0.970** | **22.8%** | **0.0623** | **2.61 (0.271)** |
| **Category B only** | Ding 2025 | 1 | Not poolable | Reported AUC 0.802 (0.672–0.933) | n/a | n/a | n/a | n/a |

This sensitivity analysis was not specified in the registered protocol or analysis-plan amendment and is therefore reported as post hoc. The Category-A-only estimate remained close to the full-pool estimate, but its confidence and prediction intervals were wider. Accordingly, this analysis assesses robustness to exclusion of the Category B study and does not constitute a direct comparison of ROI constructions or support claims of superiority or equivalence.

## Table S11. TRIPOD-SRMA checklist

*Checklist for transparent reporting of systematic reviews and meta-analyses of prediction model studies (TRIPOD-SRMA; Snell KIE, Levis B, Damen JAA, et al. BMJ 2023;381:e073538). Item wording below is abridged; the full item text is given in the source publication. Location pointers refer to the numbered sections of the manuscript (jcm-4512392); the manuscript is paginated at typesetting, so section and table anchors are given instead of page numbers. Status: Reported / Partially reported / Not applicable, judged by the authors against the manuscript and its Supplementary Materials.*

| Section | Item (abridged) | Location in manuscript | Status | Notes |
|---|---|---|---|---|
| **Title** | Identify the report as a systematic review (with/without meta-analysis) of prediction model studies, indicating the models' purpose and target | Title; Abstract heading | **Reported** | Title names the systematic review and meta-analysis, the modality (CT radiomics), the target condition (RCC), and the predicted outcomes (grade, stage, APF) |
| **Abstract** | Structured summary: background, objectives, data sources, eligibility, appraisal and synthesis methods, results, limitations, conclusions, registration | Abstract | **Reported** | Structured (Background/Objectives, Methods, Results, Conclusions); PROSPERO CRD420251150155 stated; certainty ratings and the risk-of-bias burden (72% HIGH) reported in the abstract |
| **Introduction: rationale** | Rationale for the review in the context of existing knowledge, including the models' intended use and healthcare setting | Section 1, paragraphs 1-3 | **Reported** | Clinical context (preoperative risk stratification, surgical planning for APF) and biological rationale given; prior reviews of intratumoral radiomics distinguished |
| **Introduction: objectives** | Explicit statement of the review question(s), with population, index models, outcomes, and intended use | Section 1, final paragraph | **Reported** | Three questions stated (accuracy for grade/stage/APF; incremental value over intratumoral models; radiogenomic support); full PICOTS specification resides in the registered protocol |
| **Methods: registration and protocol** | Registration information and access to the protocol; amendments declared | Sections 2.1 and 2.7 | **Reported** | PROSPERO CRD420251150155; protocol amendments declared in Section 2.7 and prespecification records referenced in Supplementary Table S10 |
| **Methods: eligibility criteria** | Inclusion and exclusion criteria, with the rationale for model-related restrictions | Section 2.3 | **Reported** | Criteria (a)-(d) plus the operational fat-ROI classification rules; the Category A/B classification is declared post hoc (developed during extraction) |
| **Methods: information sources** | All databases, registers, and other sources searched, with coverage dates | Section 2.2; Supplementary Table S1 | **Partially reported** | Eight databases searched with dates; Embase could not be searched (no institutional access; strategy written but never executed). Declared openly and examined empirically (verification searches and relative recall, Supplementary Table S12); the gap remains a genuine limitation |
| **Methods: search strategy** | Full search strategies for each source, including filters and limits | Section 2.2; Supplementary Table S1 | **Reported** | Full strings and per-database yields in Table S1; the 2015-2026 date restriction justified and verified by an inception-2014 re-run (Table S12) |
| **Methods: selection** | Methods for screening and study selection, including reviewer numbers | Section 2.4 | **Reported** | Two independent reviewers for title/abstract and full text; third- |

| Section | Item (abridged) | Location in manuscript | Status | Notes |
|---|---|---|---|---|
| **process** | and automation | | | reviewer arbitration |
| **Methods: data collection process** | Methods for data extraction, including duplication and verification | Section 2.4 | **Reported** | One extractor plus full second-reviewer verification with consensus adjudication; every pooled value verified in two independent passes against the primary full texts (Section 2.4; Supplementary Table S10) |
| **Methods: data items** | Data items sought, including the estimate-selection hierarchy when studies report multiple models/validations | Sections 2.3-2.4; Supplementary Tables S6 and S9 | **Reported** | Prespecified hierarchy (external > internal > CV > apparent; author-designated primary model; ties by first-reported, never largest AUC); within-study pairing rules stated |
| **Methods: risk of bias and applicability** | Tools and process for assessing risk of bias and applicability of included models | Section 2.5 | **Partially reported** | PROBAST applied in duplicate with arbitration, and RQS/METRICS/TRIPOD adherence assessed; PROBAST risk-of-bias domains fully tabulated (Table 3), but applicability judgements are not tabulated separately from risk of bias |
| **Methods: effect measures / summary measures** | Performance measures synthesized and their scales | Section 2.6 | **Reported** | Logit-transformed AUC with CI-derived SEs; delta-AUC on the raw scale for nested pairs; calibration not poolable (none reported in poolable form; graded descriptively in three tiers) |
| **Methods: synthesis methods** | Meta-analysis model, heterogeneity handling, and criteria for pooling | Section 2.6 | **Reported** | REML with Hartung-Knapp intervals (DL and PM sensitivity); $I^2$, $\tau^2$, Q, 95% prediction intervals; subgroups and sensitivity analyses; nesting rules and correlation grid for delta-AUC; stopping rule k >= 3 |
| **Methods: reporting bias assessment** | Methods for assessing risk of bias due to missing results | Section 2.6 | **Reported** | Assessed qualitatively by prespecified policy (all pools k < 10); Egger regression and funnel plots exploratory and confined to the supplement, labeled as such |
| **Methods: certainty assessment** | Methods for assessing certainty in the body of evidence | Section 2.6 | **Reported** | Adapted GRADE for prediction research with the target of certainty stated per outcome; domain judgements archived with the analysis record |
| **Results: study selection** | Numbers at each stage, with exclusions and reasons; flow diagram | Section 3.1; Figure 1; Supplementary Table S2 | **Reported** | Flow derived from the archived search-execution records; the attribution of 162 programmatic records that were not separately archived is footnoted in Table S1 |
| **Results: study characteristics** | Characteristics of included studies and models | Section 3.2; Table 1; Supplementary Tables S6 and S9 | **Reported** | 25 studies, 10,761 patients; per-study design, ROI construct, validation tier, and estimates with CIs |
| **Results: risk of bias** | Risk-of-bias (and applicability) assessments per study | Section 3.7; Table 3; Figure 4 | **Partially reported** | PROBAST domain and overall judgements complete per study; applicability concerns discussed narratively (reference standards, populations) rather than tabulated per domain |
| **Results: individual studies** | Performance estimates with precision for each study/model | Table 1; Figures 2-3 | **Reported** | Per-study AUC with 95% CI (or a no-CI flag with descriptive handling); nested-pair values in Supplementary Table S10 |
| **Results: syntheses** | Pooled results with heterogeneity and prediction intervals; sensitivity/subgroup results | Sections 3.3-3.6; Table 2 | **Reported** | Prediction intervals presented as the primary statement of expected performance in a new setting; all |

| Section | Item (abridged) | Location in manuscript | Status | Notes |
|---|---|---|---|---|
| | | | | sensitivity analyses from the single archived run |
| **Results: reporting biases** | Results of reporting-bias assessment | Sections 3.3-3.4; Supplementary Figure S5 | **Reported** | Exploratory Egger values and funnel plots in the supplement with caution labels; no inferential claim made |
| **Results: certainty of evidence** | Certainty ratings per outcome with domain-level reasons | Sections 3.3-3.6; Table 2; Figure 5 | **Reported** | VERY LOW (grade prediction, and the grade-family fat-versus-tumor increment); LOW (stage prediction, APF prediction, and the stage-family incremental comparisons); downgrade reasons stated per outcome |
| **Discussion: summary and interpretation** | Summarize findings in the context of the evidence and the models' intended use | Section 4, paragraphs 1, 4-6 | **Reported** | Family-specific interpretation; incremental value stated as small, assumption-dependent, and of unestablished clinical utility |
| **Discussion: limitations** | Limitations of the evidence and of the review process | Section 4, limitations paragraph | **Reported** | Retrospective designs, 72% HIGH risk of bias, outcome-construct mixing, cohort overlap (Table S12), post hoc classification, Embase gap, post-search studies disclosed but not pooled |
| **Discussion: implications** | Implications for practice and research | Sections 4 (final paragraphs) and 5 | **Reported** | No model ready to guide treatment; prospective-evaluation pathway alongside the MAP score; methodological agenda for the field |
| **Other: registration and protocol access** | Registration number and where the protocol can be accessed | Section 2.1 | **Reported** | PROSPERO CRD420251150155 |
| **Other: support/fundin g** | Sources of financial and non-financial support and role of funders | Funding; Conflicts of Interest statements | **Reported** | Najran University Growth Funding Program NU/GP/MRC/14/2384-1; funder role disclaimed |
| **Other: competing interests** | Declaration of competing interests | Conflicts of Interest statement | **Reported** | None declared |
| **Other: availability of data and materials** | Where extracted data, analytic code, and other materials can be found | Data Availability Statement; Supplementary Materials | **Partially reported** | Extracted data, the data-verification record, and the meta-analytic datasets in the Supplementary Materials; analysis scripts available from the corresponding author on request rather than in a public repository |

Summary of honestly partial items: information sources (Embase inaccessible; empirically examined, not remedied), risk-of-bias/applicability (PROBAST applicability not separately tabulated, in Methods or Results), and availability of materials (scripts on request, not deposited). All other applicable items are reported in the manuscript at the locations given.

# Table S12. Search verification, relative recall, update sweep, and cohort-overlap audit

*Four verification exercises accompany the revised search reporting: (1) an inception-through-2014 re-run of the original strategies to test the 2015 lower date bound; (2) a relative-recall (indexing) check of every study assessed at full text; (3) a documented update sweep from the search date (2026-03-29) through August 2026; and (4) an audit of shared source cohorts among included studies, with omission sensitivity analyses. Omission sensitivity values derive from the archived analysis output. All verification searches were executed 2026-08-17, and the dated search records, retrieved payloads, and flow re-derivation are archived with the review materials.*

## S12.1. Pre-2015 verification searches (inception through 2014-12-31)

The original Table S1 strategy strings were re-run verbatim (adapted only for date-restriction syntax) on every database with an accessible free API.

| Database (API) | Query | Hits |
|---|---|---|
| **PubMed (E-utilities)** | Search 1 (full protocol string) | 0 |
| **PubMed (E-utilities)** | Search 2 (broader) | 0 |
| **PubMed (E-utilities)** | Search 3 (fat + grade/stage focused) | 49 |
| **Europe PMC (REST)** | Main | 0 |
| **Europe PMC (REST)** | Broader | 1 (an ECR 2012 congress abstract book; screened, excluded) |
| **Europe PMC (REST)** | Outcome-specific | 0 |
| **CrossRef (works)** | Main | top 1,000 relevance-ranked retrieved (bag-of-words total 104,475; not a Boolean yield) |
| **CrossRef (works)** | Alternate | top 1,000 relevance-ranked retrieved (bag-of-words total 65,543) |
| **OpenAlex (works)** | Main | 0 |
| **Semantic Scholar (Graph)** | Main | 3 |

Deduplication and screening: 2,053 raw records; 2,037 unique after DOI and normalized-title deduplication; programmatic triage removed records lacking any renal/RCC term (n = 1,109) or any peritumoral/perirenal/fat term (n = 739); the remaining 189 records were individually reviewed at title/abstract level against the full eligibility criteria. **Eligible pre-2015 studies found: 0.** Four arguable near-misses (2012-2014: a linear perirenal fat-thickness predictor, qualitative “sticky fat” CT features, an attenuation-based dissection-difficulty conference abstract, and the

Mayo Adhesive Probability score derivation) were flagged for author adjudication and judged not eligible: each uses single measurements, qualitative signs, or a semiquantitative visual score, with no radiomics or texture feature extraction. Scopus, Web of Science, and IEEE Xplore could not be re-run (subscription-only exports; no API from this environment); this is documented rather than skipped, and the original strategy strings remain archived in Table S1 for independent re-execution. Conclusion: the 2015 lower date bound excluded no eligible study.

## S12.2. Relative recall (indexing check of all 38 full-text-assessed studies)

Every study assessed at full text (25 included, 13 excluded at full text) was checked for indexing in each of the five API-accessible sources.

| Source | Included studies indexed (n = 25) | Full-text-excluded studies indexed (n = 13) | Total (n = 38) |
|---|---|---|---|
| **PubMed** | 25 | 12 | 37 |
| **Europe PMC** | 25 | 12 | 37 |
| **CrossRef** | 24 | 13 | 37 |
| **OpenAlex** | 25 | 13 | 38 |
| **Semantic Scholar** | 23 | 13 | 36 |

**All 38 studies were indexed in at least three of the accessible sources** (35 in all five, 1 in four, 2 in three; minimum = 3). The least-covered records were Xu 2025 (Chinese-language journal; PubMed, Europe PMC, OpenAlex) and Feng 2024 (excluded at full text; CrossRef, OpenAlex, Semantic Scholar). No study assessed at full text depended on a single source, and none was retrievable only through the subscription databases; this is the empirical substitute for the inaccessible Embase search, not a demonstration that Embase would have added nothing.

## S12.3. Update sweep (2026-01-01 onward; executed 2026-08-17)

The primary strategies were re-run on the five API-accessible databases restricted to 2026 publication/creation dates: 691 rows retrieved; 579 unique after deduplication; 60 recognized as members of the original screening corpus (including 5 of the 6 already-included 2026 studies); **519 new unique records screened** at title/abstract against the protocol criteria. Verdicts: 515 excluded with coded reasons (E1 population n = 359; E10 review/editorial/case report n = 56; E5 no peritumoral/perirenal fat segmentation n = 43; E6 no radiomics exposure n = 43; E11 conference abstract n = 7; E4 non-CT n = 4; E3 animal n = 2; E8 no in-scope outcome n = 1), and 4 flagged as potentially eligible. Under the data freeze, no flagged study was added to the review; all are disclosed here and referred to the editor-disclosure pathway.

| # | Flagged record | DOI | Assessment |
|---|---|---|---|
| **1** | Peritumoral fat radiomics for dual prediction of TNM stage and histological grade in ccRCC (Diagnostics 2026;16(7):1099; 474 patients, three cohorts, concentric perirenal-fat ROIs 1-10 mm) | 10.3390/diagnostics16071099 | Eligible (squarely inside PICO; primary outcomes grade and stage); published after the search date; disclosed, not pooled |
| **2** | CT-based radiomics ensemble model (CRIPEM) for preoperative prediction of pathological upstaging in ccRCC (Cancers 2026;18(10):1558; 309 cT1 patients, three centres, external validation) | 10.3390/cancers18101558 | Eligible (Category B peritumoral-includes-fat; endpoint family matches pT3a upstaging); published after the search date; disclosed, not pooled |
| **3** | Multiscale prediction of tumor micronecrosis and progression in ccRCC (Academic Radiology 2026; n = 468, multicentre) | 10.1016/j.acra.2026.04.051 | Borderline: intratumoral plus peritumoral CT radiomics, but the outcome (micronecrosis/progression) sits at the edge of the prespecified outcome set |
| **4** | Radiomics-based machine learning versus the | 10.29271/jcpsp.2026.06.761 | Borderline: tumour and perirenal fat |

| # | Flagged record | DOI | Assessment |
|---|---|---|---|
| | MAP score for predicting haematocrit decline after laparoscopic partial nephrectomy (JCPSP 2026;36(6):761) | | radiomics in RCC, but the perioperative outcome is outside the prespecified outcome set |

Neither eligible study contradicts the direction of the review's findings; both would be incorporated in any future update (Discussion, limitations paragraph).

## S12.4. Cohort-overlap audit (shared source cohorts among included studies)

Nine of the 25 included studies draw wholly or partly on public imaging cohorts. Roles verified against the two-pass extraction record (cohort and tier fields per model).

| Public source | Study | Role of the shared cohort in that study | Enters a primary pooled estimate? |
|---|---|---|---|
| **TCGA-KIRC (TCIA-hosted)** | Zhou Z 2021 | Entire study cohort (n = 203): development and internal random split | Yes: grade pool (internal estimate 0.848) |
| **TCGA-KIRC (TCIA-hosted)** | Yang 2025 | External validation cohort (TCGA, n = 175) | Yes: grade pool (external estimate 0.92) |
| **TCGA-KIRC (TCIA-hosted)** | Mei 2025 | Training cohort (MAP-scored TCGA-KIRC kidneys); externals are RHWU and KiTS23 | Training only; the pooled APF estimate is the RHWU external cohort (0.890) |
| **TCIA (TCGA multi-scanner collection)** | Huang 2026 | Training (n = 148) and internal test (n = 38) sets; external validation is the authors' hospital | Yes: stage pool (internal estimate 0.79 is evaluated on TCIA-derived patients) |
| **TCIA-KIRC** | Wang 2026 | External test set (n = 50) | Yes: stage pool (external estimate 0.82) |
| **TCIA (CPTAC-CCRCC + TCGA-KIRC/KIRP/KICH)** | Yuan 2025 | Second external set (n = 204) | No: the pooled estimate is the first-reported bi-center external cohort (0.902); the TCIA estimate (0.806) enters sensitivity analysis only |
| **KiTS19** | Li S 2024 | External validation set (n = 143) | Yes: grade pool (external estimate 0.573); no other grade-pool study uses KiTS19 |
| **KiTS23** | Mei 2025 | Second external validation set (n = 400 kidneys per side) | No: sensitivity substitution only (0.677 left / 0.766 right) |
| **KiTS23** | Li S 2026 | External Validation Cohort IV (n = 156) | No: the pooled estimate is Validation Cohort I (0.659); Cohort IV enters sensitivity analysis only |
| **KiTS23** | Triantafyllou 2026 | Entire cohort (n = 354) | No: contributes to no pooled estimate (descriptive subtype endpoint) |
| **None found** | Zhou J 2026 and the remaining 15 included studies | Institutional cohorts only | - |

**Overlap inside primary pools and omission sensitivity.** Two pools contain more than one estimate drawing on the same public source, so patient-level overlap cannot be excluded (patient identifiers are not recoverable from the primary reports):

- **Grade pool (TCGA-KIRC): Zhou Z 2021 and Yang 2025.** Zhou Z's whole cohort and Yang's external validation cohort both derive from TCGA-KIRC. Omission sensitivity (REML, governing mKH intervals for the k = 5 omission pools, from the canonical run): omitting Zhou Z, pooled AUC 0.757 (0.528-0.896; $I^2$ = 94.9%); omitting Yang, 0.718 (0.563-0.835; $I^2$ = 86.1%); the full-pool estimate is 0.772 (0.599-0.884).

- **Stage pool (TCIA-hosted TCGA imaging): Huang 2026 and Wang 2026.** Huang's training/internal-test patients and Wang's external test set both derive from TCIA collections. Omission sensitivity (governing

mKH intervals, k = 5): omitting Huang, 0.819 (0.640-0.920; $I^2$ = 78.9%); omitting Wang, 0.814 (0.629-0.919; $I^2$ = 78.7%); the full-pool estimate is 0.813 (0.681-0.899).

No two members of the APF pool share a source cohort at the tier entering the pooled estimate (Mei's TCGA and KiTS23 exposure sits in training and sensitivity tiers respectively). The same two stage-pool studies (Wang, Huang) also contribute nested delta-AUC pairs; the leave-one-out results for those pools are reported in Supplementary Table S10.3. Because Yuan 2025's TCIA external cohort contains TCGA-KIRC patients, the Yuan-TCIA sensitivity substitution (stage pooled AUC 0.777, 0.676-0.854) is additionally not free of TCGA overlap with Huang and Wang; the primary analysis, which uses Yuan's bi-center cohort, is. Estimates were stable to every single omission, but stability under omission does not rule out residual correlated evidence, and this is carried as a limitation in the Discussion.

## Supplementary Figures

All meta-analytic figures were generated from the single archived analysis run; each value shown is traceable to the archived analysis output. Figures S8-S10 summarize the feature-level synthesis, which is unaffected by the model-composition criteria applied to the pooled analyses.

# Figure S1. Forest plot: grade prediction, fat-only pool (k = 6)

*Pooled AUC 0.772 (95% CI 0.599-0.884; $I^2$ = 93%; $\tau^2$ = 0.55); 95% prediction interval 0.298-0.964. Individual AUCs ranged from 0.573 (Li S 2024, external) to 0.92 (Yang 2025, external). Marker shape encodes fat ROI methodology (Category A, explicit fat segmentation; Category B, peritumoral ring); colour encodes the fat-model validation tier. Pooling used REML with Hartung-Knapp intervals on the logit-AUC scale. Luo 2022 and Chen 2025 do not contribute a fat-only estimate under the model-composition criteria of Section 2.3 and are therefore absent from this pool.*

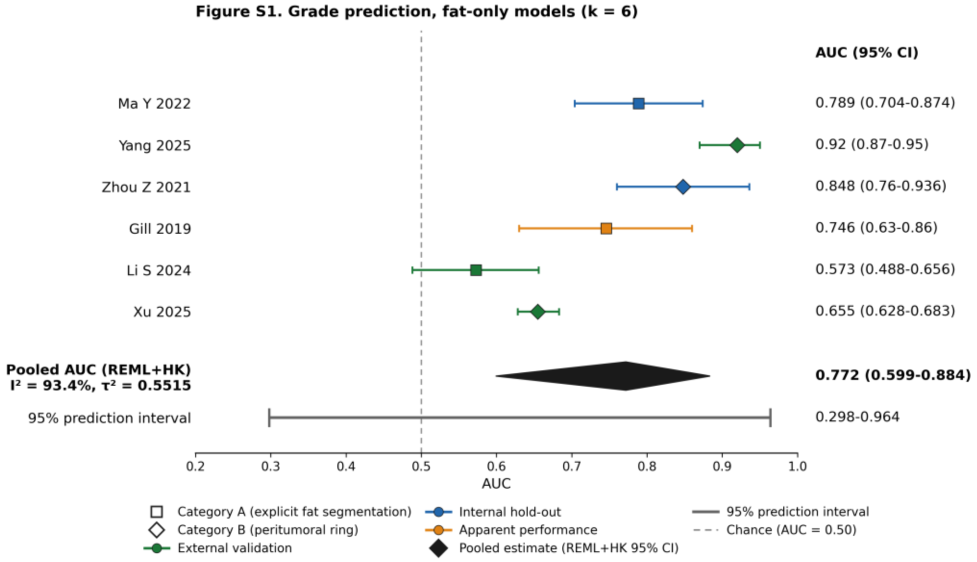

## Figure S2. Forest plot: stage-family prediction, fat-only pool (k = 6)

*Pooled AUC 0.813 (95% CI 0.681-0.899; I² = 72%); 95% prediction interval 0.460-0.957. Wu 2024 (0.762, 0.676-0.849; external) contributes an externally validated peritumoral estimate whose confidence interval is reported in the study's electronic supplementary material. The two largest studies report divergent results (Yuan 2025, n = 1,516, AUC 0.902; Li S 2026, n = 1,661, AUC 0.659), contributing substantially to heterogeneity.*

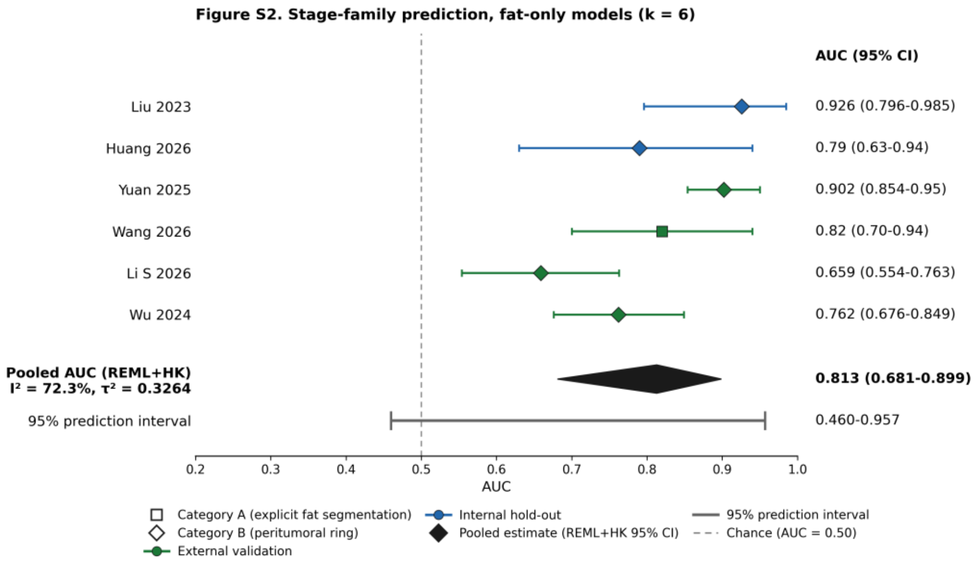

## Figure S3. Forest plot: APF prediction (k = 4)

*Pooled AUC 0.848 (95% CI 0.738-0.917; $I^2$ = 0%; $\tau^2$ = 0). Three studies used explicit adipose segmentation (Category A) and one a geometric perirenal band (Category B; Ding 2025); all four sample the perirenal fat compartment. The most consistent pooled estimate across all outcomes; GRADE certainty LOW, downgraded two levels for risk of bias (4/4 HIGH PROBAST; reference-standard indirectness in Mei 2025 disclosed).*

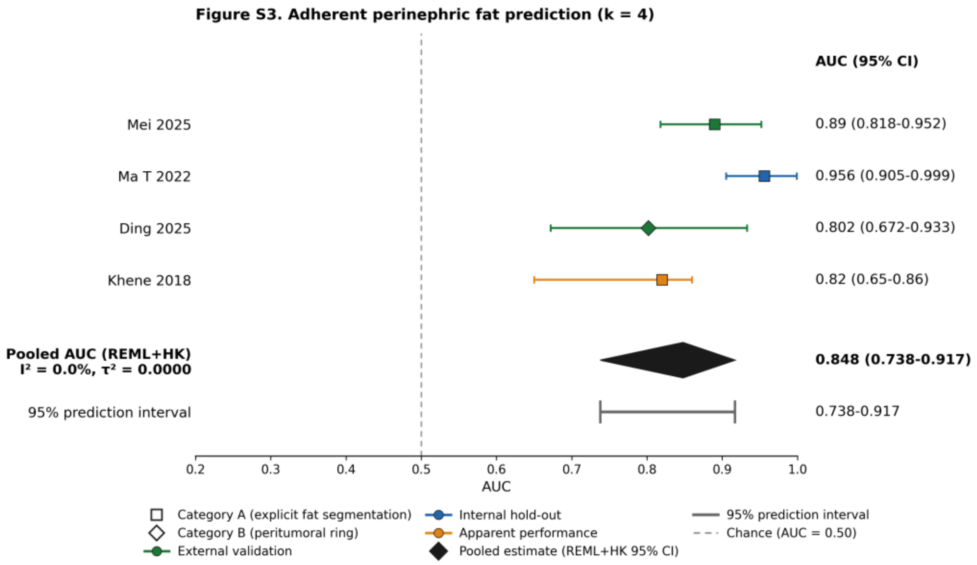

# Figure S4. Exploratory Category A versus B subgroup analysis (grade pool)

*Three studies per arm; the governing modified Hartung-Knapp (mKH) intervals are shown (k = 3 per arm). Category A (explicit fat): pooled AUC 0.703 (0.365-0.907; I² = 80%). Category B (peritumoral ring): 0.828 (0.314-0.981; I² = 94%). Between-subgroup test QM = 1.18, p = 0.34. Exploratory and underpowered: with intervals this wide, no conclusion about equivalence or difference between ROI approaches is warranted.*

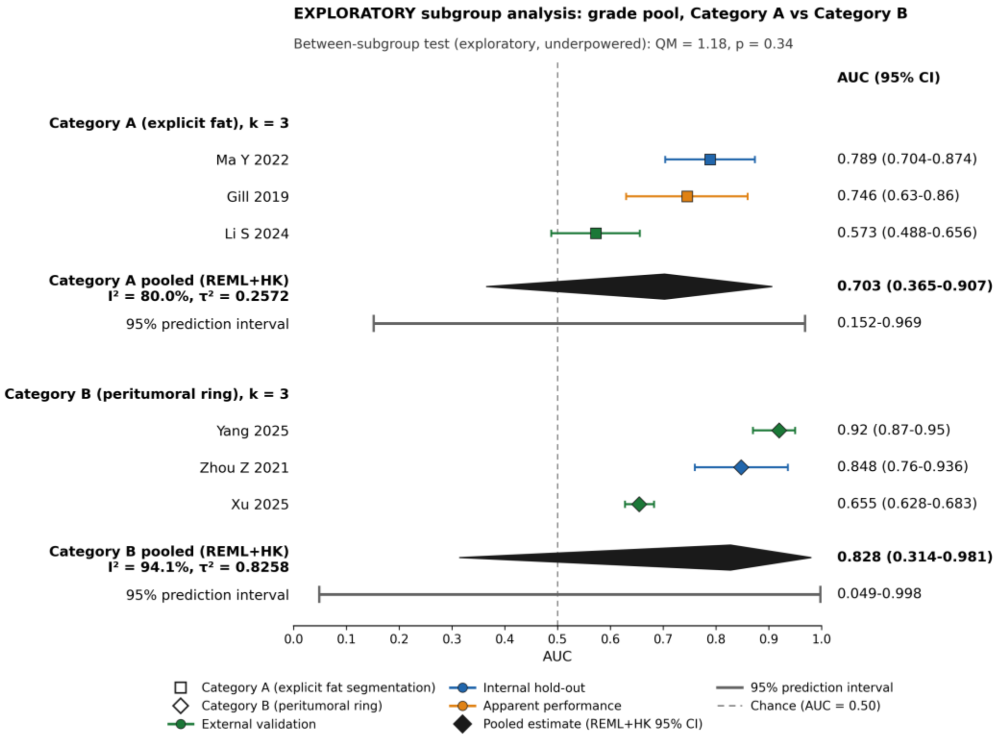

# Figure S5. Exploratory funnel plots, fat-only pools (logit scale)

*Egger regression is exploratory only and carries no inferential claim: every pool contains fewer than ten studies, so no inferential small-study-effect testing was undertaken (prespecified policy, Methods 2.6). Grade: z = 1.678, p = 0.169 (k = 6). Stage: z = 1.103, p = 0.332 (k = 6). APF: z = 1.038, p = 0.409 (k = 4). Any asymmetry may reflect small-study effects, publication bias, or methodological heterogeneity between studies; these cannot be distinguished from the test alone.*

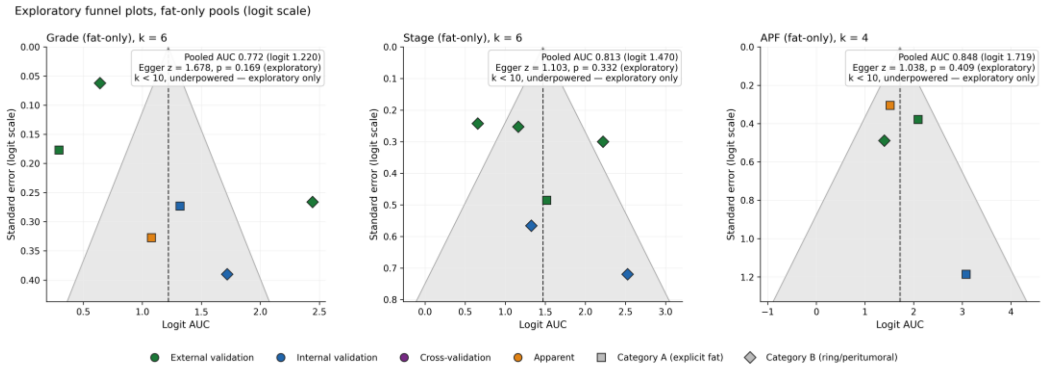

# Figure S6. Leave-one-out sensitivity: most influential omission per fat-only pool

*Grade: omitting Yang 2025 shifted the pooled AUC by 0.053 to 0.718 ($I^2$ = 86%). Stage: omitting Yuan 2025 shifted it by 0.042 to 0.771 ($I^2$ = 51%). APF: omitting Mei 2025 shifted it by 0.022 to 0.826 ($I^2$ = 0%). No omission changed the direction or interpretation of any pool.*

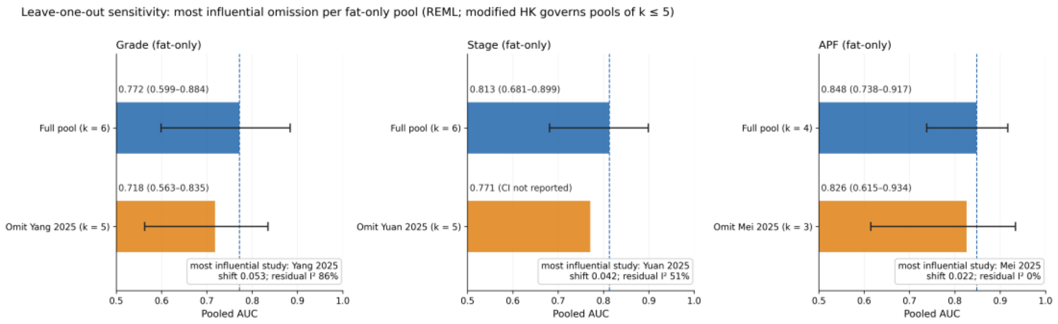

## Figure S7. Sensitivity comparison of pooled AUCs across prespecified subsets

*Grade: full pool (k = 6) 0.772; external-only (k = 3) 0.752 (mKH 0.150-0.981); PROBAST low-risk (k = 3) 0.809 (mKH 0.303-0.976); n >= 200 (k = 5) 0.778 (mKH 0.543-0.911); TCGA-overlap omissions 0.757 (omit Zhou Z 2021) and 0.718 (omit Yang 2025). Stage: full pool (k = 6) 0.813; external-only (k = 4) 0.797 (mKH 0.565-0.922); PROBAST low-risk (k = 3) 0.810 (mKH 0.346-0.972); n >= 200 (k = 5) 0.795 (mKH 0.633-0.897); Yuan TCIA substitution (k = 6) 0.777 (0.676-0.854); TCGA-overlap omissions 0.819 (omit Huang 2026) and 0.814 (omit Wang 2026). APF: full pool (k = 4) 0.848 (mKH 0.738-0.917); surgical-reference-only (k = 3) 0.826 (mKH 0.615-0.934); Mei KiTS23 substitution (k = 4) 0.781 (mKH 0.556-0.910). For every subset of five or fewer studies the modified Hartung-Knapp (mKH) interval governs. APF showed the tightest intervals across subsets.*

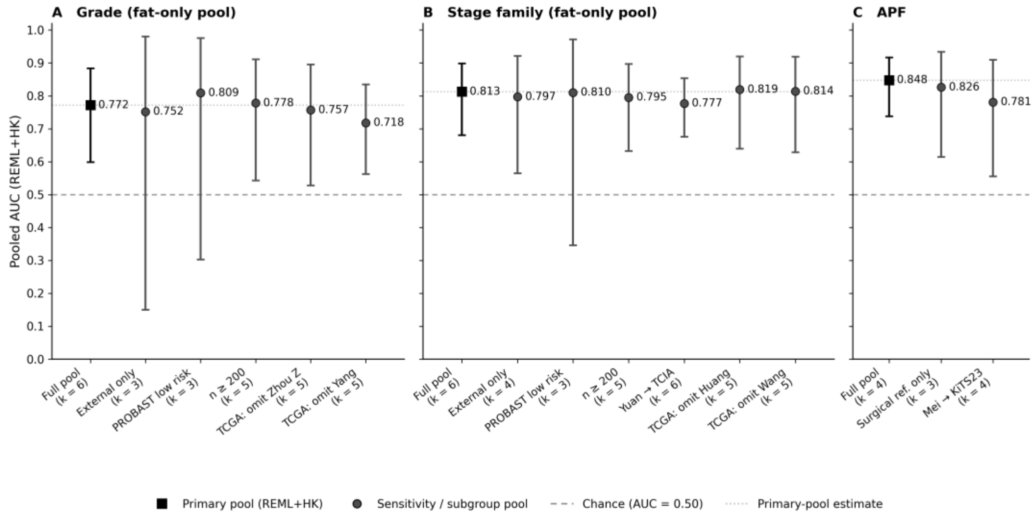

## Figure S8. Feature convergence heatmap

*Feature-level synthesis, unaffected by the model-composition criteria applied to the pooled analyses. Busyness (NGTDM) appeared in 7 of the 18 studies reporting named features (39%), followed by SizeZoneNonUniformity, ZoneEntropy, Kurtosis, and Correlation (each 5/18). Texture features dominated.*

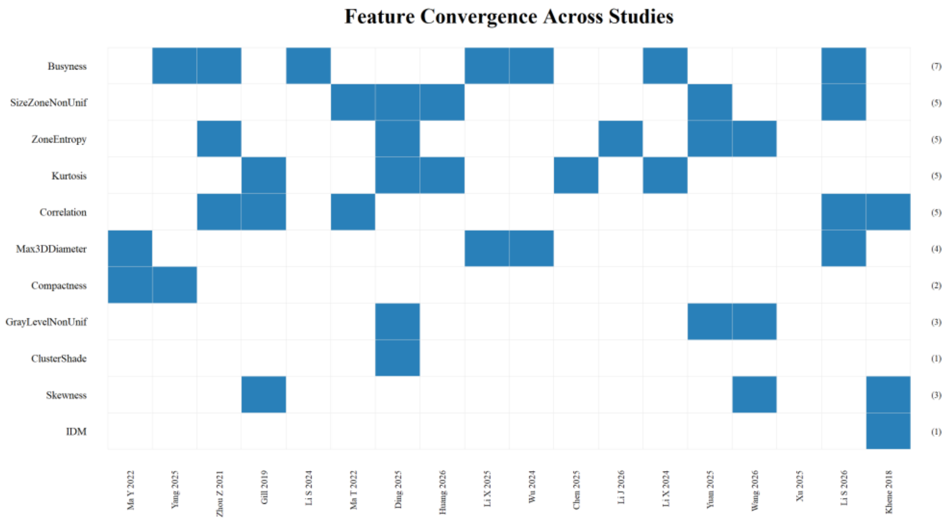

## Figure S9. Feature class distribution

*Texture features (GLCM, GLSZM, NGTDM, GLRLM, GLDM) accounted for 69% of selected features, first-order 19%, and shape 12%.*

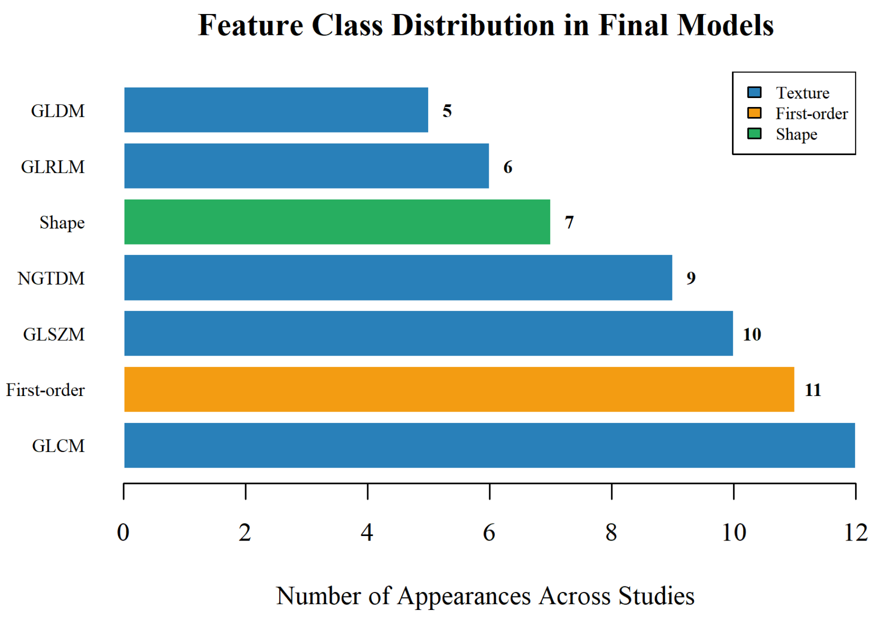

## Figure S10. AUC versus sample size (bubble plot)

*No clear association between sample size and AUC. The largest studies (Yuan 2025, Li S 2026) report divergent stage-family results, consistent with the heterogeneity retained in the stage pool (I² = 72%).*

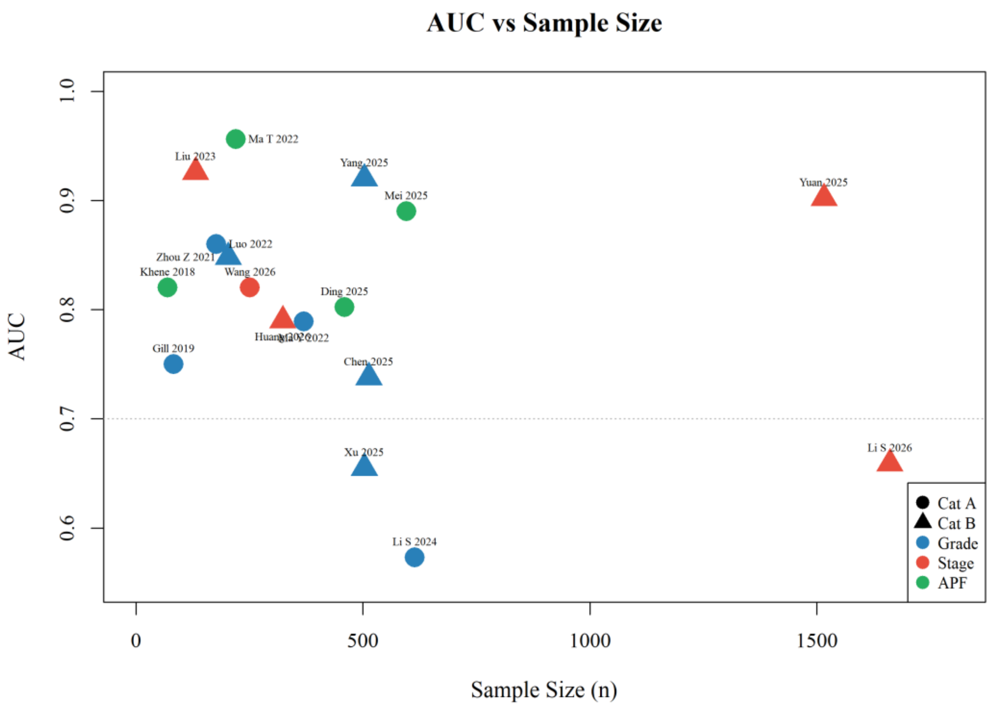

## Figure S11. PROBAST full traffic-light plot

*Domain-level and overall judgements for all 25 studies. 18/25 (72%) rated HIGH overall risk of bias, driven by Domain 4 (Analysis; 18/25 HIGH), predominantly insufficient events-per-variable ratios and absent external validation of the fat-specific model. Domains 1 and 3: 0% HIGH; Domain 2: 16% HIGH.*

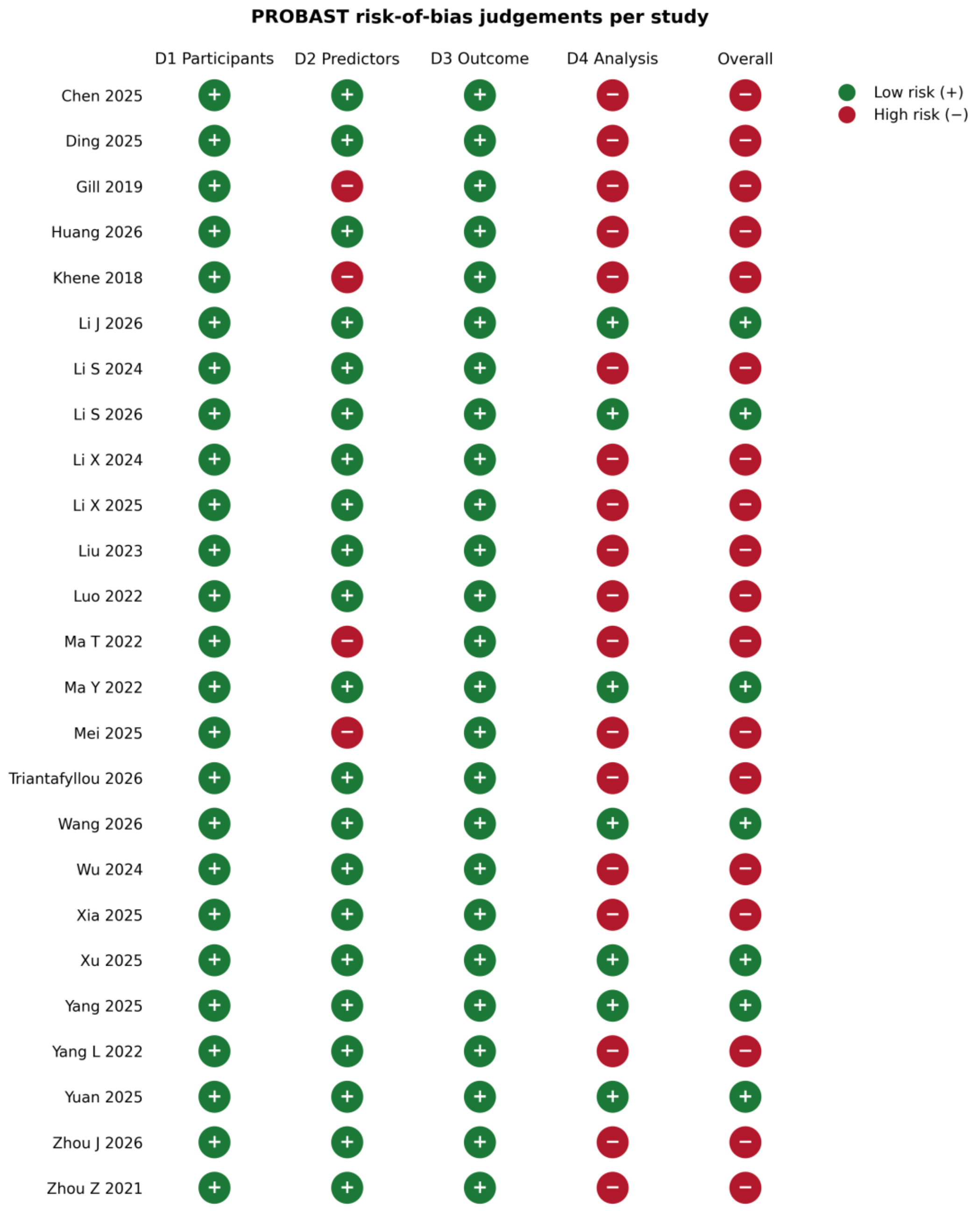

# Figure S12. Per-study RQS totals

*Median 13/36 (36%); range 3-22 (8-61%). Xu 2025 (17/36) and Chen 2025 (15/36) reflect item-level scoring of the calibration item of numeric calibration statistics; the median is unchanged (Table S10, item 17). No study included phantom calibration or a prospective design, and only two acquired imaging at multiple time points.*

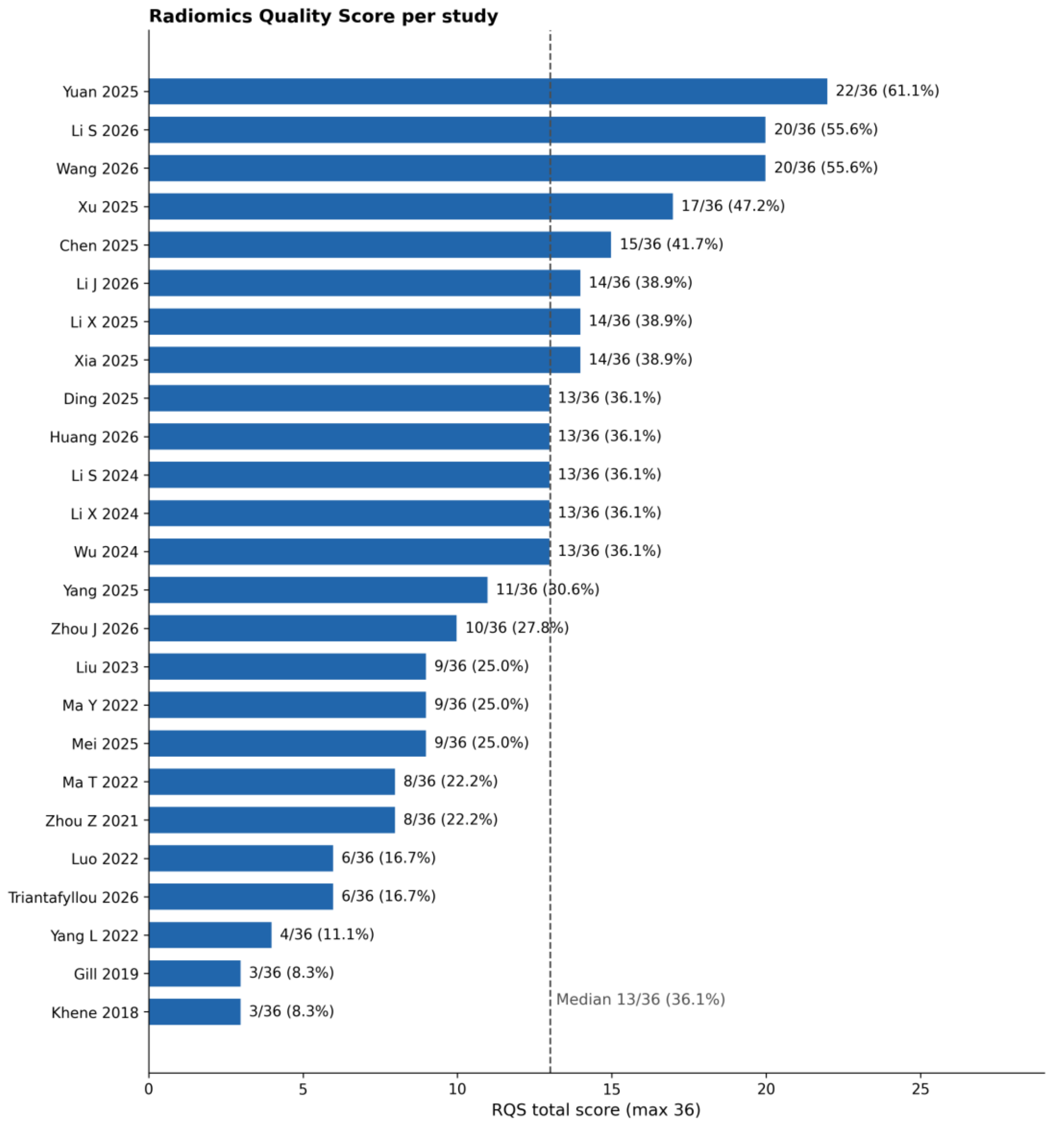

## Figure S13. Per-study METRICS scores

*Median 76.4% (range 38.9-90.4%). Six studies rated Excellent (>= 80%), 15 Good (60-79%), three Moderate (40-59%), and one Low (< 40%).*

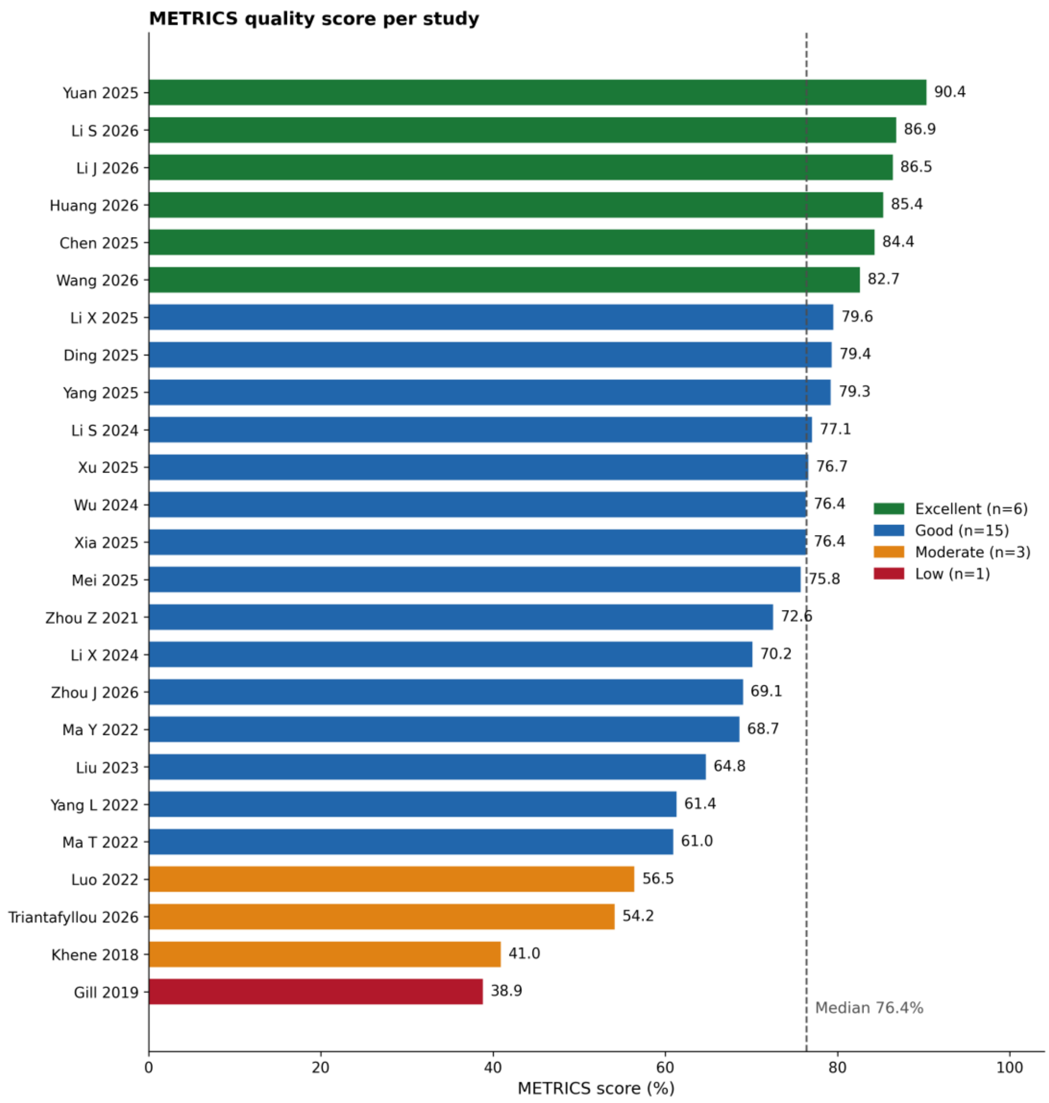

## Figure S14. TRIPOD item adherence

*Median study-level adherence 67% across the 12 key applicable items. Four items were reported by all 25 studies; three items were reported by five or fewer studies, two of them by none (including handling of missing data).*

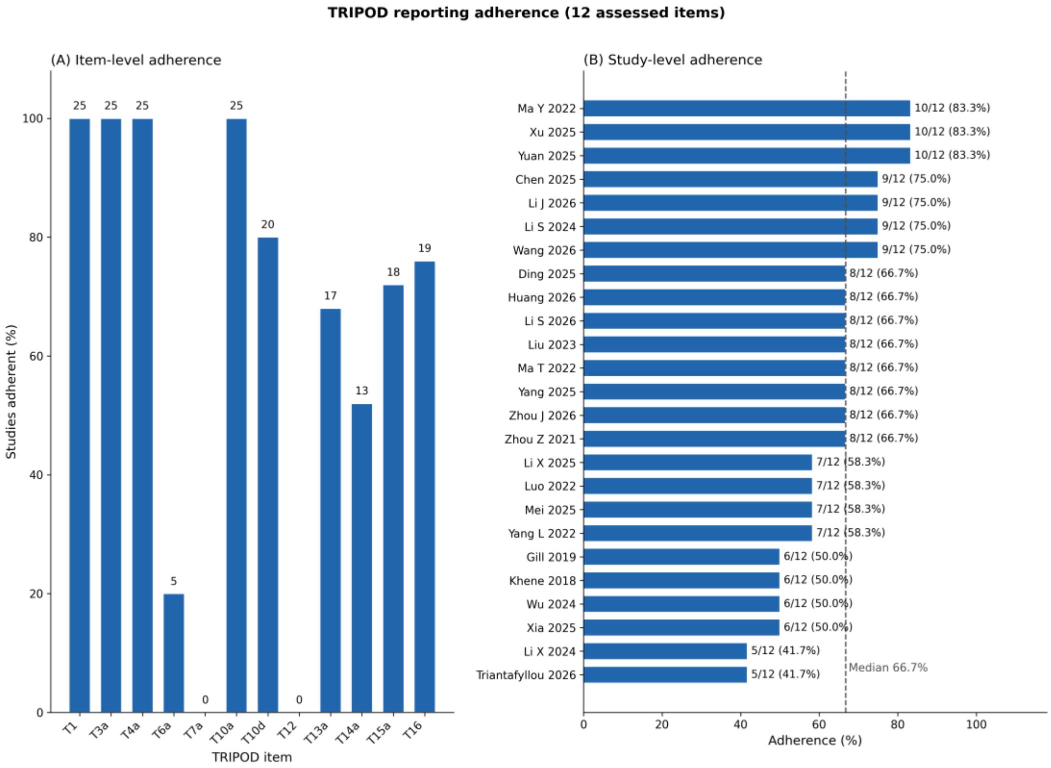

## Figure S15. RQS versus METRICS concordance

*Spearman rho = 0.895 (p < 0.001) across the 25 studies. METRICS scores run systematically higher because the tool emphasizes achievable methodological standards rather than aspirational criteria such as phantom studies.*

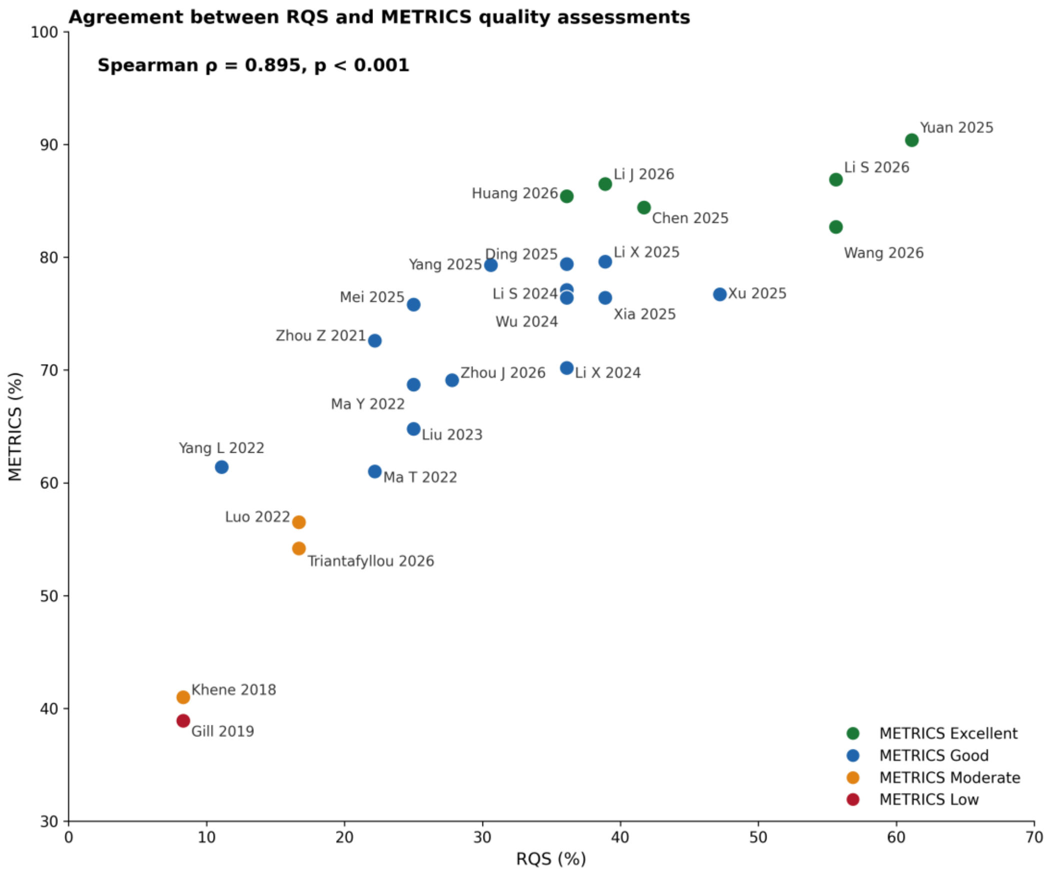

# Figure S16. Study characteristics summary (nine panels)

*(A) Publication year (2018-2026); (B) country (China 22, USA 1, France 1, Greece 1); (C) sample size (range 70-1,661; median 354; total 10,761 patients); (D) fat ROI classification (Category A 9, Category B 16); (E) primary outcome (grade 9, stage family 7, APF 4, other 5); (F) fat-specific model validation tier (external 11, internal hold-out 6, cross-validation 2, apparent 2, no separately validated fat tier 4 (tiers are assigned from the validation design of the study's fat-related model where one exists, and from the study-level design otherwise; studies reporting no separately validated fat-model tier form the final group)); (G) machine-learning algorithm; (H) study design (multicenter 16, single-center 9); (I) feature-extraction software (PyRadiomics 19/25).*

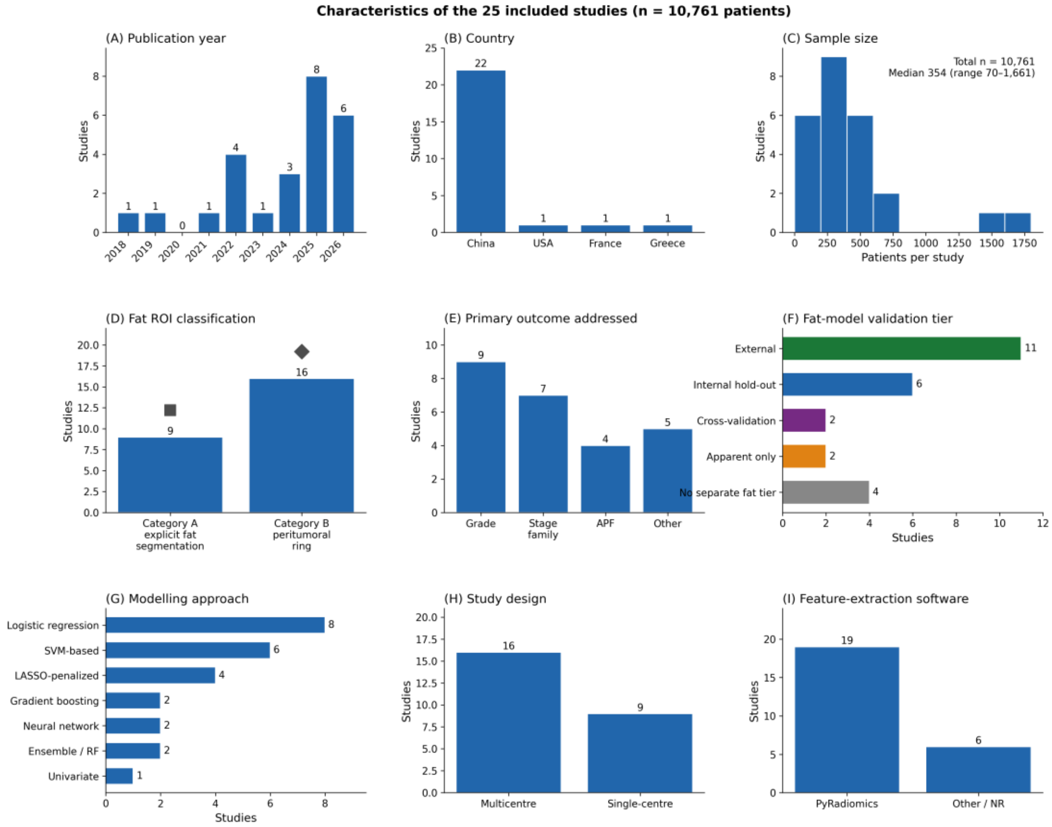

**Figure S17. Category-A-only sensitivity analysis for adherent perinephric fat prediction.** *Forest plot restricted to the three studies using explicit adipose segmentation (Category A). The pooled AUC was 0.863 (governing modified Hartung-Knapp 95% CI 0.643–0.956; I² = 22.8%; τ² = 0.0623; k = 3), with a 95% prediction interval of 0.547–0.970. Category B was represented by one APF study and was therefore not poolable separately.*

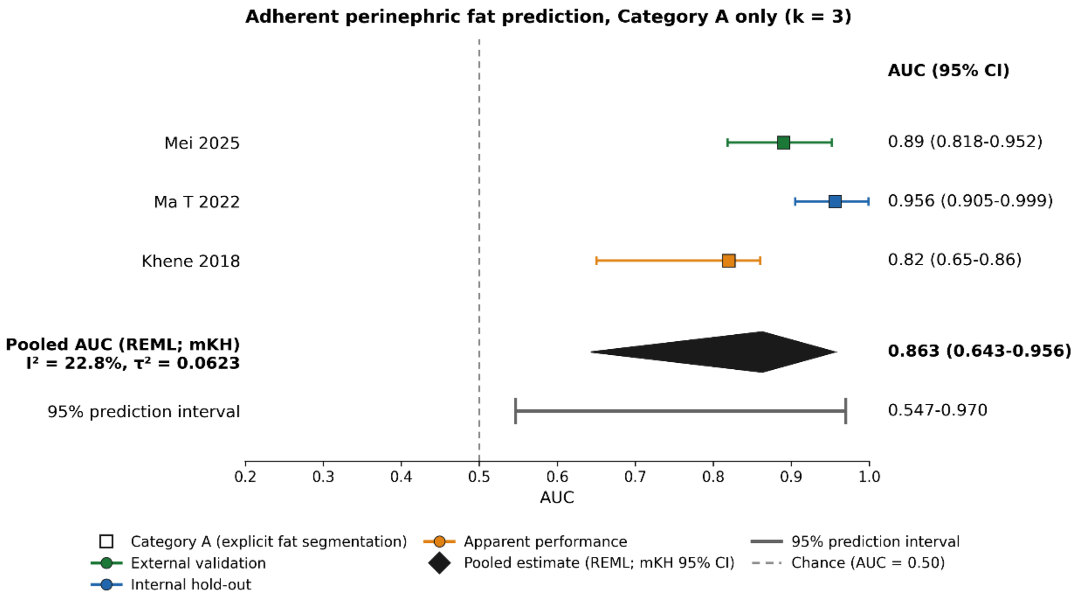

Supplement: Supplementary file 1 [file jcm-15-06720-s001.zip › jcm-4512392-supplementary.pdf]
